# Vegetarian Dietary Patterns and Diet-Related Metabolites Are Associated With Kidney Function in the Adventist Health Study-2 Cohort

**DOI:** 10.1053/j.jrn.2025.11.001

**Published:** 2025-11-25

**Authors:** Fayth M. Butler, Roy O. Mathew, Lars E. Sveen, Gary E. Fraser

**Affiliations:** * Adventist Health Study, Research Affairs, Loma Linda University, Loma Linda, California.; † Center for Nutrition, Healthy Lifestyles and Disease Prevention, School of Public Health, Loma Linda University, Loma Linda, California.; ‡ School of Public Health, Loma Linda University, Loma Linda, California.; § Division of Biochemistry, Department of Basic Sciences, School of Medicine, Loma Linda University, Loma Linda, California.; ¶ Division of Nephrology, Department of Medicine, Loma Linda VA Healthcare System, Loma Linda, California.; ** Division of Nephrology, Department of Medicine, Loma Linda University School of Medicine & Loma Linda University Health, Loma Linda, California.; †† Division of Cardiology, Department of Medicine, Loma Linda University School of Medicine & Loma Linda University Health, Loma Linda, California.

**Keywords:** kidney function, creatinine, vegetarian dietary patterns, metabolomics, cohort

## Abstract

**Objectives:**

Chronic kidney disease is a significant global health burden. Plant-based diets have shown potential benefits for kidney function, but the specific impacts of vegetarian dietary patterns and underlying metabolic mechanisms remain unclear.

**Methods:**

Serum creatinine was measured in 899 Adventist Health Study-2 cohort participants who were classified as vegan, lacto-ovo-vegetarian, pesco-vegetarian, semivegetarian, or nonvegetarian, and estimated glomerular filtration rate (eGFR) was calculated (<60 or >/ = 60 mL/min/1.73 m^2^). Untargeted plasma metabolomics using ultra-performance liquid chromatography–mass spectrometry/mass spectrometry was performed on a subset of participants with serum creatinine measurements. Multivariable linear regression models adjusted for confounders assessed associations between eGFR, dietary patterns, and plasma metabolites. The Storey permutation approach was used to adjust for false discovery.

**Results:**

Vegan and pesco-vegetarian dietary patterns were associated with significantly higher eGFR compared to nonvegetarians, with minimal attenuation after adjustment. Metabolomics analysis revealed distinct profiles in relation to creatinine, comparing the 90th to 10th percentiles. Creatinine was positively associated with several lipid and amino acid subclasses, which were lower in participants following vegetarian dietary patterns. Key subclasses included acetylated peptides, histidine metabolism, lysine metabolism, plasmalogens, and notably sphingomyelins for pesco-vegetarians. Individual metabolites within many of the differential subclasses consistently showed opposite directions of associations with vegetarian dietary patterns and creatinine.

**Conclusions:**

Vegan and pesco-vegetarian dietary patterns, relative to nonvegetarian, were associated with higher eGFR, potentially through diet-driven metabolic pathways. Plant-based diets may offer protection against chronic kidney disease by modulating key metabolic pathways.

## Introduction

CHRONIC KIDNEY DISEASE (CKD) affects over 800 million people globally, posing significant health and economic burdens, including over $85 billion in Medicare expenditures in the US in 2020.^1,2^ Diet significantly impacts kidney health, and plant-based dietary patterns may offer a cost-effective strategy for CKD prevention.

The beneficial effects of plant-based dietary patterns on CKD risk and progression have become increasingly appreciated. Several epidemiological studies have linked healthy dietary patterns—defined overall by higher consumption of healthy plant foods such as fruit, vegetables, legumes, nuts, whole grains, unsaturated fats, and lower consumption of unhealthy or fatty animal products and processed or sugary foods—with lower incidence of CKD.^3–7^ Findings from the Adventist Health Study-2 (AHS-2) cohort show that vegetarian dietary patterns are associated with lower incidence of hypertension^8^ and diabetes,^9^ key risk factors for CKD, consistent with lower prevalence of metabolic syndrome. Notably, a recent AHS-2 study also found that vegetarian dietary patterns were strongly associated with reduced mortality from renal disease.^10^ However, it is not clear how vegetarian dietary patterns—particularly those defined by the types and degree of animal products consumed—are associated with kidney health.

Understanding the role of diet-associated metabolites is essential in understanding how different dietary patterns influence kidney disease. Previous metabolomics analyses in AHS-2 revealed that vegans had higher plasma levels of polyphenol-derived, microbial, and vitamin-related metabolites, while nonvegetarians had higher levels of proinflammatory lipids and amino acids.^11^ These distinct plasma metabolic profiles may contribute to differences in CKD risk and progression across dietary patterns. This study examines associations between vegetarian dietary patterns, kidney function, and diet-related metabolites, using estimated glomerular filtration rate (eGFR) as a measure of kidney health.

## Methods

### Study Design/Population

The AHS-2 cohort consists of ~96000 seventh-day Adventist members enrolled 2002–2006, ~50% of whom follow vegetarian or plant-based dietary patterns.^12^ The cohort’s shared cultural and religious beliefs influence lifestyle practices such as low tobacco and alcohol use, which reduce confounding, though potentially limiting generalizability, as even the nonvegetarians are health conscious and have lower meat consumption relative to the general population.

Participants completed a detailed food frequency questionnaire (FFQ) at baseline, assessing frequency of consumption for over 200 food items, including fruits, vegetables, legumes (lentils, soybeans, and other beans), grains, soy-based foods and drinks, dairy, meats, poultry, fish, and other dietary components or supplements. Dietary intake data were collected and calculated using the Nutrition Data System for Research software versions 4.06 and 5.03.^13^ Validity of dietary intake has been assessed extensively using 24-hour diet recalls and biomarkers,^14^ and methods have been developed to control measurement error.^15,16^ Participants also completed biennial health and hospitalization history forms, which were used to track new diagnoses and hospitalizations, and capture changes in dietary or other exposures.^17^

Dietary patterns were classified a priori based on responses to the FFQ and defined as follows: Vegans never or rarely (less than once per month) consumed eggs, dairy, fish, and other meats; lacto-ovo-vegetarians consumed eggs and dairy more than once a month but fish and other meats less than once a month; pesco-vegetarians consumed fish at least once a month but all other meats less than once a month; semivegetarians consumed nonfish meats at least once a month and any meat, including fish, less than once per week; and nonvegetarians consumed nonfish meats at least once a month and any meat (including fish) more than once per week. These vegetarian dietary groups have been shown to have very different intakes of plant and animal foods.^18^

A Calibration substudy nested within the AHS-2 cohort (n = 1,011) assessed dietary validity and included oversampling of Black Americans.^19^ Study participants completed 2 sets of three 24-hour dietary recalls as well as an additional FFQ. A number of biomarkers of dietary intake were measured in these participants, besides various cardiometabolic or other clinical markers. Serum creatinine was measured in 901 participants. After exclusion of 2 participants not providing complete information on dietary habits necessary for ascertainment of dietary pattern, the analytical cohort included 899 participants from the calibration substudy.

Informed consent was obtained from all participants in this study. The study adhered to the principles outlined in the Declaration of Helsinki. Approval for the project was granted by the university’s institutional review board.

### Laboratory Assays

Serum creatinine was measured using a standard enzymatic assay with standardization to isotope creatinine standards at the institution’s Medical Center Clinical Laboratory.

Serum creatinine was used to calculate eGFR, using the race-free eGFR equation.^20^ eGFR was categorized into accepted stages of CKD according to the Kidney Disease: Improving Global Outcomes CKD staging framework.^21^

A subset of 139 participants was selected from the AHS-2 calibration study based on the availability of both serum creatinine measurements and metabolomics data, which included vegan, pesco-vegetarian, and nonvegetarian dietary groups. Untargeted metabolomics using ultra-performance liquid chromatography–mass spectrometry/mass spectrometry profiling was performed by Metabolon, Inc. (Morrisville, NC) using the Global Assay (DiscoveryHD4), which combines 4 using ultra-performance liquid chromatography–mass spectrometry/mass spectrometry methods in both positive and negative ion modes to optimize detection of chemically diverse metabolites, as described previously.^22,23^ Samples were prepared for metabolomics analysis with standardized protocols and quality controls to monitor performance. Median relative standard deviations for internal standards and endogenous metabolites were calculated to assess instrument (6%) and process (8%) variability. Metabolites were identified by comparison to a reference library of purified standards and recurrent unknown entities. Compounds were classified into various biochemical classes and pathways, including amino acid, nucleotide, carbohydrate, lipid, xenobiotic, and microbial metabolism. All metabolomics preprocessing and statistical analyses were conducted using R version 4.3.2 (R Foundation for Statistical Computing, Vienna, Austria).

### Statistical Analysis

#### Linear Regression

Linear regression models were generated to examine associations of dietary pattern with eGFR or serum creatinine, comparing vegan, lacto-ovo-, pesco-, or semivegetarian with nonvegetarian dietary patterns (in the analytical creatinine cohort of n = 899). Fold change was calculated as the ratio of the estimated marginal (adjusted) arithmetic mean of untransformed eGFR values of each diet group relative to nonvegetarians. Models included adjustment for race (Black vs. non-Black, including White, Hispanic, Asian, and other), sex (defined as male/female on self-report and presumably corresponding to sex assigned at birth), education (high school, some college, college graduate), smoking (ever/never), alcohol drinking (current or former/never), age at creatinine measurement, creatinine batch, body mass index (BMI, kg/m2), and chronic disease indicators, including prevalent diabetes at clinic/blood collection, treatment for hypertension over the last 12 months (where missing variables coded as “no” for both variables), cardiovascular events, including coronary bypass, angioplasty/stent, carotid artery surgery, heart attack, congestive heart failure and stroke, and lastly prevalent cancer excluding nonmelanoma skin cancer.

Linear regression models were also generated to examine associations of individual plasma metabolites with eGFR among a subset of n = 139 participants with overlapping serum creatinine measurements and metabolomics data. Metabolites were excluded if ≥ 80% missing, and minimum values imputed for blanks before median scaling and log transformation of metabolites.

To evaluate associations between dietary pattern and metabolite abundance using linear regression, fold change was calculated for each metabolite from the ratio of the log of the geometric means, then back transformed. To examine associations of creatinine (or eGFR) with metabolite abundance, fold change was calculated from the ratio of adjusted geometric means corresponding to the 90th and 10th percentiles of creatinine. For analyses of metabolite subclasses, individual component metabolite (median-standardized) values within a subclass were averaged to obtain a composite mean for that subclass, and *t*-scores obtained by dividing by the standard deviation of the composite means, accounting for the covariances between the metabolites. The number of significant or differential metabolites at false discovery rate (FDR) <0.05 for each subclass was determined, along with the number of differential metabolites positively or inversely associated with the exposure. Regression models were generated comparing plasma metabolic profiles of vegans or pesco-vegetarians with non-vegetarians, or vegans with pesco-vegetarians, with adjustment for sex, race, age at clinic visit, creatinine batch, metabolomics batch, study/substudy, and BMI.

#### False Discovery

To estimate the FDR, an adapted Storey et al.^24^ permutation approach was used, and residualized variables for diet or creatinine were permuted to obtain the null distribution of the *t* scores for metabolite abundance,^25^ thereby, retaining covariances between residualized metabolite abundances.

## Results

### Descriptive Characteristics

Table 1 shows the baseline characteristics of 899 participants. A higher proportion of Black participants, compared with non-Black participants, were nonvegetarian and pesco-vegetarian (*P* < .0001). Vegans were, on average, the oldest (*P* <.001), while nonvegetarians had the highest BMI and were more likely to report smoking and alcohol use (*P* <.0001). Hypertension was more common among semivegetarians and nonvegetarians, and diabetes prevalence was highest in nonvegetarians (*P* = .02), along with history of smoking and alcohol intake (*P* <.001). Total energy intake was highest in pesco-vegetarians (*P* = .045). There were no statistically significant differences in unadjusted creatinine concentrations or CKD. Given the older age distribution of the cohort (mean age: 66.7 years), the prevalence of CKD stage 2 is not unexpected and may reflect age-related physiological decline in eGFR rather than clinical kidney disease.

### Associations of Dietary Patterns With Estimated Glomerular Filtration Rate

To understand how dietary patterns impact kidney health, we examined associations of 4 vegetarian dietary patterns relative to a nonvegetarian dietary pattern with eGFR (Table 2). The *ß* coefficients represent the mean difference in eGFR compared with nonvegetarians. In the simplest model (model 1), which adjusted for basic demographic and technical variables, significantly higher eGFR was observed among vegans (*ß* = 4.6, *P* =.01). This association persisted across all models. Additional adjustment for education and lifestyle variables (model 2) slightly strengthened associations, with a pesco-vegetarian dietary pattern reaching marginal significance (*ß* = 3.2, *P* = .05). Further adjustment for BMI (model 3) somewhat attenuated the associations, but the fully adjusted model 4 revealed significant associations for both vegan (*ß* = 4.0, *P* = .043) and pesco-vegetarian (*ß* = 3.6, *P* = .038) patterns. No associations were observed for lacto-ovo-vegetarian or semivegetarian patterns in any model. When all vegetarian patterns (excluding semivegetarians) were combined, a significant association with higher eGFR was observed in model 2 (*ß* = 2.1, *P* = .046), with a trend toward significance in the other models.

Given the older age distribution of the cohort (mean age 66.7 years), we examined associations between dietary pattern and eGFR separately for participants aged ≥65 years (Supplementary Table 1) and those <65 years (Supplementary Table 2). Among participants over 65, a pesco-vegetarian dietary pattern was associated with significantly higher eGFR in models 1 and 2 (*P* =.04 and *P* =.03), with associations approaching significance in models 3 and 4. A vegan dietary pattern was also associated with significantly higher eGFR in model 2 in this older group (Supplementary Table 1). Among participants under age 65, a vegan dietary pattern was associated with significantly higher eGFR in the minimally adjusted model (*P* =.04), with attenuated significance in models 2 through 4, although the positive trend persisted (Supplementary Table 2).

We additionally examined associations of dietary pattern with eGFR stratified by race and sex, as overall differences between Black and White participants appeared to be driven by sex-specific patterns. No associations between dietary pattern and eGFR were observed among Black females in any model (Supplementary Table 3). Among Black males, vegan dietary pattern was associated with higher eGFR, with a trend toward significance in model 2 (*ß* = 10.8, *P* =.07), which was attenuated in more fully adjusted models (Supplementary Table 4). Among White females, a vegan dietary pattern was significantly associated with higher eGFR in models 1 and 2 (*P* ≤.01), and a pesco-vegetarian dietary pattern was significantly associated with higher eGFR across all models, including the fully adjusted model (model 4: *ß* = 8.6, *P* = .005) (Supplementary Table 5). Similarly, among White males, vegans had significantly higher eGFR in models 1–3 (*P* < .05), with a positive trend evident in the fully adjusted model (Supplementary Table 6).

### Associations of Individual Metabolites With Creatinine or Dietary Pattern in the Subset of Adventist Health Study-2 Participants With Metabolomic Measurements

#### Associations of Serum Creatinine With Plasma Metabolites

Comparisons of metabolites between participants in the 90th and 10th percentiles of creatinine identified 296 significantly different metabolites (FDR <0.05), all of which positively correlated with creatinine (Supplementary Table 7). Compounds with associations over 2-fold were primarily from food component/plant and benzoate metabolism subclasses. These creatinine-associated metabolites provided a framework for subsequent analyses.

#### Joint Associations of Metabolites With Creatinine and Vegetarian Dietary Patterns

Next, we sought to identify metabolites that help explain associations of vegetarian dietary patterns with eGFR by extracting metabolites associated with both creatinine and dietary pattern to identify overlapping signals.

To explore metabolites that might explain associations between vegetarian diets and eGFR, we identified those linked with both creatinine and dietary patterns. The various comparisons described in these analyses are depicted in Supplementary Figure 1A. First we identified metabolites associated with vegan relative to nonvegetarian dietary patterns (Supplementary Table 8) and high versus low serum creatinine (Supplementary Table 7) and discovered a total of 113 metabolites significantly associated with both (Supplementary Table 9). Of these, all 113 associations with creatinine were positive; however, only 17 of the metabolite associations with vegan dietary pattern were positive, and 96 were inversely associated (Supplementary Table 9). Metabolites showing greatest fold changes comparing highest with lowest serum creatinine, and that were also in lowest abundance in vegans, included piperine metabolites, histidine metabolites, other food component metabolites, and putative uremic toxins such as p-cresol glucuronide and 3-methyl catechol sulfate. Next, we identified creatinine-associated metabolites significantly associated with a pesco-vegetarian relative to nonvegetarian dietary pattern (Supplementary Table 10), and discovered 87 metabolites (Supplementary Table 11). In contrast to the exclusively positive metabolite associations with creatinine, only 12 metabolites associated positively with a pesco-vegetarian diet, whereas the other 75 were inversely associated (Supplementary Table 11). Again, histidine-derived metabolites and food component/plant metabolites showed the largest positive associations with creatinine paralleled by strong inverse associations with a pesco-vegetarian diet. For the vegan versus pesco-vegetarian comparison, there were 107 metabolites associated with both creatinine (Supplementary Table 7) and a vegan versus pesco-vegetarian diet (Supplementary Table 12), where 44 positively associated with a vegan dietary pattern and 63 inversely associated (Supplementary Table 13). Metabolites showing largest positive associations with creatinine and inverse associations with vegan relative to pesco-vegetarian diet again included piperine metabolites, 3-methylhistidine, as well as 3-carboxy-4-methyl-5-propyl-2-furanpropanoate, besides ectoine, and p-cresol glucuronide (Supplementary Table 13).

There were 46 creatinine-associated metabolites in common in analyses comparing vegan and pesco-vegetarian with nonvegetarian dietary patterns (Supplementary Table 14). All metabolites were positively associated with creatinine, and most—but not all—showed inverse associations with vegetarian dietary patterns (Supplementary Fig. 2, Supplementary Table 14). Compounds showing marked differences and in opposite directions included histidine metabolites N-acetyl-1-methylhistidine, 1-methyl-5-imidazoleacetate, and 3-methylhistidine, as well as 3-carboxy-4-methyl-5-propyl-2-furanpropanoate (lower in vegans only), quinate, sulfate of piperine metabolite, homocitrulline, phenylacetylglutamate, hydantoin-5-propionate, and p-cresol sulfate.

### Linear Regression of Metabolite Subclasses

#### Associations with Creatinine

We performed similar analyses as discussed above, identifying differential metabolite subclasses instead of individual metabolites associated with vegetarian dietary patterns and serum creatinine to highlight key metabolic groups (Supplementary Fig. 1B). All metabolite subclasses were positively associated with serum creatinine, consistent with results from analyses of individual metabolites (Table 3, Supplementary Table 7). Metabolite subclasses showing greatest fold changes comparing high versus low creatinine included guanidino and acetamido metabolism, histidine metabolism, and food component/plant metabolism (*P* < 8.6 × 10^−5^). Other subclasses significant at *P* < .0001 included lysine metabolism, aminosugar metabolism, urea cycle, arginine and proline metabolism, phospholipid metabolism, and purine metabolism (Table 3).

#### Joint Associations with Creatinine and Vegan Versus Nonvegetarian Dietary Patterns

**Figure 1.**
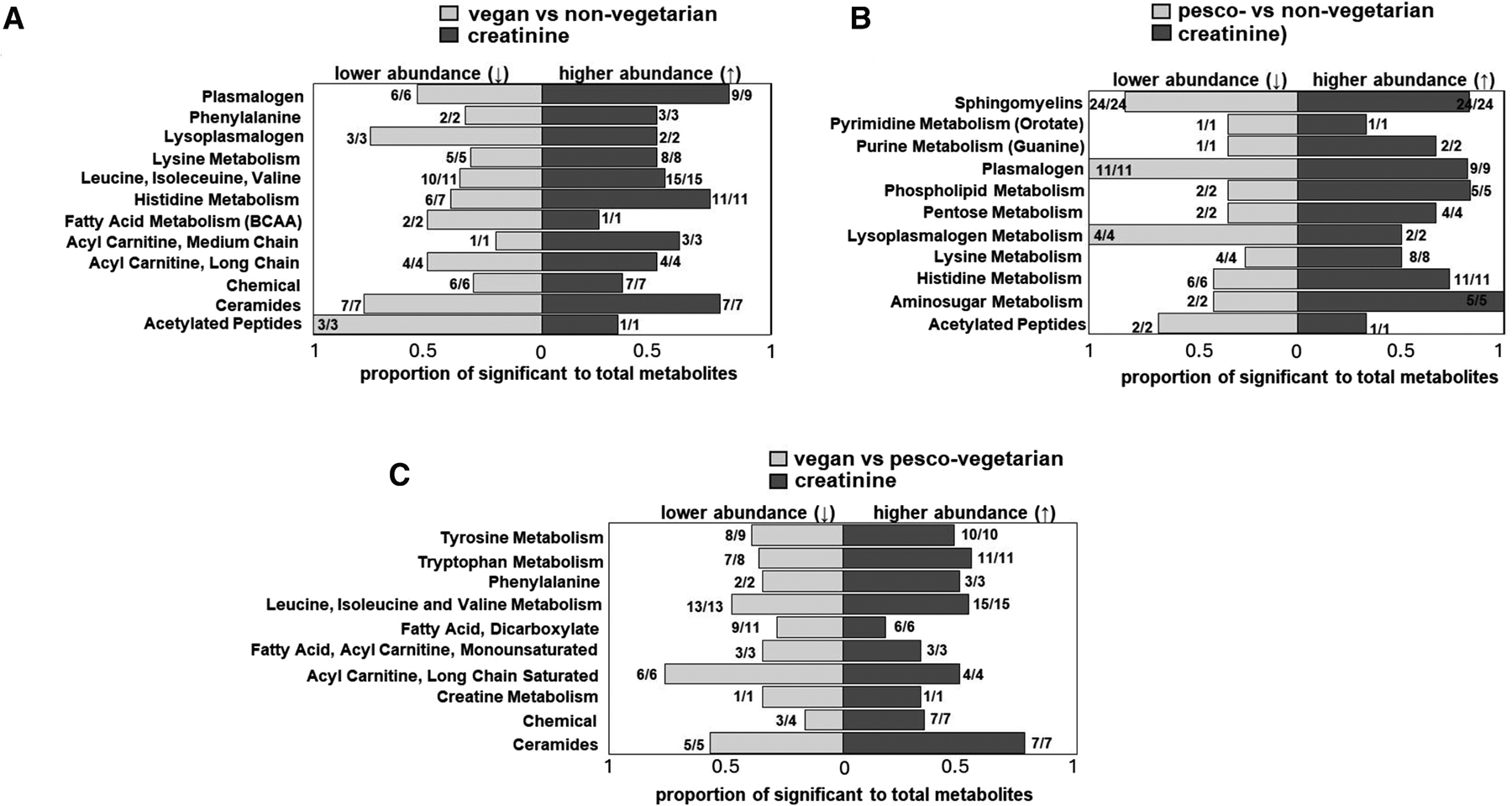
Differential subclasses showing opposite associations of dietary patterns and serum creatinine with plasma metabolites. Differentially abundant subclasses were determined after calculating the composite mean (averaging component metabolites) for each dietary group and subsequently t-scores. Bars reflect proportion of differential metabolites (FDR <0.05) to total metabolites on panel associated positively with creatinine (right of 0) or inversely with the indicated dietary pattern (left of 0). Ratios represent the number of significant metabolites associated in the same direction (inversely or positively) out of the total number of significant metabolites in the subclass. Only contrasting subclasses (comparing dietary pattern and creatinine associations with metabolites) are shown. The predominant subclass direction, reflecting the overall direction of component metabolites, is plotted for contrasts of creatinine with (A) vegan versus nonvegetarian, (B) pesco-vegetarian versus nonvegetarian, or (C) vegan versus pesco-vegetarian. BCAA, branched chain amino acid; FDR, false discovery rate.

In comparing vegan with nonvegetarian dietary patterns (Supplementary Table 15) relative to creatinine, 14 significant subclasses were identified, with all except 2 (vitamin A metabolism and fatty acid acyl glycine) inversely associated with a vegan diet (Table 4). Subclasses positively associated with creatinine but present at lowest abundance (fold change <0.7) in vegans included acetylated peptides, histidine metabolism, ceramides, fatty acid metabolism (including branched chain amino acid and acyl carnitine), and plasmalogens/lysoplasmalogens (Table 4). Notably, most of the component metabolites in all overlapping subclasses showed consistent associations that were positive with creatinine and inverse with a vegan diet (Fig. 1A). Subclasses with the largest proportion of significant metabolites in one or both comparison groups included most notably plasmalogens and lysoplasmalogen, ceramides, and acetylated peptides (Fig. 1A, Table 4).

#### Joint Associations with Creatinine and Pesco-Versus Nonvegetarian Dietary Patterns

Next, we compared subclasses of metabolites found to be significantly different in both the pesco-vegetarian versus nonvegetarian and high versus low creatinine analyses.

Among creatinine-associated subclasses significantly different comparing pesco-vegetarian with nonvegetarian dietary patterns, a pesco-vegetarian dietary pattern was inversely associated with all metabolic subclasses (Supplementary Table 16), in contrast to the consistently positive associations noted for creatinine (Table 5, Fig. 1B). These included several of the same subclasses differentially abundant in the vegan versus nonvegetarian analysis—plasmalogen and lysoplasmalogen, histidine metabolism, acetylated peptides, and lysine metabolism (Table 5)—besides others. The sphingomyelins subclass (which was significant for pesco-vegetarians but not vegans relative to nonvegetarians) showed the most stark contrast, with strong homogeneity, where 24 out of 24 significant metabolites were inversely associated with a pesco-vegetarian dietary pattern, and 24 out of 24 significant metabolites were positively associated with creatinine. The individual component metabolites within all metabolic subclasses consistently showed inverse associations with pesco-vegetarian (vs. nonvegetarian) dietary pattern and positive associations with creatinine (Fig. 1B).

#### Joint Associations With Creatinine and Vegan Versus Pesco-vegetarian Dietary Patterns

We also identified plasma metabolite subclasses associated with serum creatinine and vegan versus pesco-vegetarian dietary patterns (Supplementary Table 17), and discovered 17 subclasses (Supplementary Table 18). Among those subclasses, the lowest in vegans were fatty acids (particularly acyl carnitines) and ceramides, besides various subclasses of amino acids (Supplementary Table 18, Fig. 1C). There were several creatinine-related subclasses that associated positively with a vegan relative to pesco-vegetarian diet (aminosugar metabolism, ascorbate and aldarate metabolism, vitamin A, phosphatidylethanolamine, phospholipid, and plasmalogen subclasses), besides those associated inversely (Supplementary Table 18).

#### Joint Associations With Creatinine, Vegan Versus Nonvegetarian, and Pesco- Versus Nonvegetarian Dietary Patterns

Lastly, we determined metabolic subclasses in common in analyses comparing vegan with nonvegetarian, pesco-vegetarian with nonvegetarian, and high versus low creatinine (90th vs. 10th percentile) (Supplementary Table 19). There were 5 subclasses with overlapping significance. Among these subclasses, histidine and lysine metabolism were most strongly associated with creatinine; acetylated peptides and histidine metabolism were lowest in vegetarians and highest among participants with higher creatinine (Supplementary Table 19).

## Discussion

In light of growing evidence for a preventive role of healthy or plant-based dietary patterns in kidney disease, we investigated how vegetarian dietary patterns influence kidney function and explored potential mechanisms using untargeted metabolomics. This study focused on a population with generally mild reductions in kidney function, largely reflective of age-related decline. Our findings show that vegetarian dietary patterns, particularly vegan and pesco-vegetarian, are associated with higher eGFR and distinct metabolomic signatures, suggesting reduced lipid accumulation and metabolic byproducts that may benefit kidney function.

Our finding of higher eGFR in vegans and pesco-vegetarians likely reflects the reno-protective effects of nutrient-dense plant foods rather than differences in muscle mass, although we acknowledge that direct measures of muscle mass were not available. In the AHS-2 cohort, vegetarians were found to have a significantly lower risk of mortality from renal failure (hazard ratio: 0.52; 95% confidence interval: 0.38, 0.70),^10^ consistent with our findings of better kidney function among vegans and pesco-vegetarians. Prior analyses have shown that AHS-2 vegans and other vegetarians consume more fruits, vegetables, legumes, and soy relative to nonvegetarians, which may account for these benefits.^18^ In contrast, lacto-ovo-vegetarians did not show particular benefit, potentially due to higher dairy intake, which may contribute to greater phosphorus load,^26^ especially from dairy sources where phosphorus is highly bioavailable and co-occurs with calcium. Furthermore, because BMI may also act as a mediator in the relationship between dietary pattern and kidney function, we fit models both with and without BMI. Positive associations of vegetarian diets with eGFR were only mildly attenuated after adjusting for BMI and cardiometabolic conditions, suggesting largely independent effects.

Positive associations of vegetarian dietary patterns with eGFR were evident among both younger (<65 years) and older (≥65 years) participants for vegans, although significance was diminished in stratified analyses due to reduced sample size and power. Interestingly, a pesco-vegetarian dietary pattern was significantly associated with higher eGFR only among older participants, possibly because dietary effects become more apparent in individuals with higher baseline risk or reflect the cumulative impact of long-term dietary habits. While the exact reasons are not clear, this is consistent with a previous observation of reduced mortality from renal failure among older pesco-vegetarians.^10^

We also observed sex-specific differences by race, with stronger associations in White participants overall. The lack of association among Black females, unlike other subgroups that showed positive associations or trends, may reflect differences in dietary exposures, sociocultural factors, or unmeasured biological/genetic, and environmental influences.

Our findings align with studies linking plant-based dietary patterns to lower CKD risk and slowed progression.^5,6,27–29^ For example, adherence to the dietary approaches to stop hypertension^29^ healthy eating index,^5^ Mediterranean,^27^ or alternative Mediterranean diets has been associated with lower CKD risk, with effect sizes of 13–20%, and slowed CKD progression.^28,30–32^ However, our study differs from a younger Italian cohort (mean age 45.9 years), which showed higher eGFR with higher intakes of plant proteins, grains, and eggs, but lower eGFR with higher consumption of animal and fish protein.^33^ This discrepancy may reflect differences in population characteristics, particularly the nearly 20-year age gap between the Italian and AHS-2 cohorts, potentially influencing serum creatinine levels through lean muscle mass differences.^14,34^ In contrast, results from the AHS-2 cohort reveal the potential impact of longer adherence to healthy diets on kidney function.^17^

Plasma metabolites, predominantly lipids and amino acids, were less frequently found in vegetarians but associated positively with serum creatinine levels. This suggests that lower availability of these metabolites may reflect either protective effects of vegetarian diets on kidney function or reduced accumulation due to impaired renal clearance. Given the broad panel of metabolites, both explanations are likely relevant. At any rate, plant-based diets consistently altered lipid metabolism, which is apparently linked to alterations in creatinine. Both vegetarian groups had lower sphingolipids, including ceramides (vegans) or sphingomyelins (pesco-vegetarians), and vegans also had lower plasmalogens. Acylcarnitines, which have essential roles in fatty acid transport, were significantly lower in vegans, consistent with patterns observed in cardiometabolic^35–37^ and kidney diseases.^38–40^ Their lower levels may indicate protection against metabolic dysfunction or serve as markers of disrupted fatty oxidation and mitochondrial dysfunction, often linked to CKD progression and inflammation. However, their precise roles and dietary influences remain unclear.

Histidine-derived metabolites were higher in vegans and pesco-vegetarians, potentially reflecting CKD-related accumulation. Vegetarians had lower levels of lysine metabolites, including trimethyllysine, a precursor of trimethylamine N-oxide linked to cardiovascular disease,^41^ and n,n,n-trimethyl-5-aminovalerate, which may be elevated in CKD patients.^42^ Similarly, branched chain amino acids leucine, isoleucine, valine—precursors for acylcarnitine synthesis—and tryptophan metabolites, which are putative uremic toxins linked to CKD progression, were lower in vegetarians, although reductions in vegans may reflect dietary intake rather than clearance.^43–48^ Other uremic toxins, including compounds from urea, arginine, and proline metabolism, as well as p-cresol sulfate, were lower in vegetarians but positively associated with creatinine, potentially reflecting inflammation-related vascular changes in CKD.^49–54^

Our findings of lower representation of amino acids and lipids, including proinflammatory compounds, in vegetarians are consistent with prior findings in the AHS-2 cohort^11,55^ and reinforce the observed benefits of vegetarian diets on cardiometabolic health.^8,56–58^ Collectively these findings, including higher abundance of amino acids associated with meat intake (nonvegetarian diet)^59,60^ and higher creatinine levels observed in nonvegetarians, support the association between red meat or animal protein and increased CKD risk or reduced renal function.^4,61–63^ In contrast, plant protein has been associated with lower risk for CKD^64–66^ and reduced mortality among CKD patients,^67^ supporting a role for plant-based diets in promoting overall kidney health.

Key strengths of our study are the inclusion of multiple distinct vegetarian diet groups that reflect variations in animal product intake, a relatively large sample size with sufficient power to detect significant associations between dietary patterns and eGFR, and the use of an untargeted, validated metabolite panel to capture a broad spectrum of kidney function–related compounds. However, several limitations should be noted. First, the cross-sectional design limits our ability to determine causality and directionality of metabolite–eGFR associations. Second, although the metabolomics subset was adequately powered to detect key differences, its modest size may have limited our ability to identify more subtle associations. Third, we relied on serum creatinine to estimate eGFR, which can be influenced by muscle mass and dietary protein intake. While we applied the race-free eGFR equation to improve accuracy, future studies should consider incorporating cystatin C or combined equations to better account for non-GFR determinants. In addition, although BMI was included to account for body size, it is not a direct measure of body composition, and bioelectrical impedance data were not available for this subset. Lastly, although the AHS-2 cohort’s shared and unique lifestyle practices help reduce confounding, they may limit the generalizability of our findings.

## Conclusions

In summary, vegetarian dietary patterns (vegan and pesco-vegetarian) were associated with lower serum creatinine compared to nonvegetarian dietary patterns, suggestive of better kidney function, potentially through reduced accumulation of metabolites linked to CKD risk, including lipids and amino acids involved in metabolic dysregulation. These metabolic profiles, particularly in vegans, suggest that plant-based diets may mitigate pathways contributing to kidney dysfunction and hold promise for promoting kidney health. These findings are especially relevant to older individuals with stage 2 or higher CKD, a group at increased risk for disease progression, for whom dietary strategies may have the greatest preventive potential.

## Supplementary Material

1

2

Supplementary Data

Supplementary data related to this article can be found at https://doi.org/10.1053/j.jrn.2025.11.001.

## Figures and Tables

**Table 1.** Demographic and Lifestyle Characteristics of AHS-2 Participants With Serum Creatinine Measurements*

	Nonvegetarian	Semivegetarian	Pesco-Vegetarian	Lacto-Ovo-Vegetarian	Vegan	*P* Value
Participants	429	41	99	251	79	
Sex						.19
Male	141 (33)	14 (34)	30 (30)	99 (39)	21 (27)	
Female	288 (67)	27 (66)	69 (70)	152 (61)	58 (73)	
Race						<.001
Black	231 (54)	13 (32)	61 (62)	65 (26)	33 (42)	
Non-Black	198 (46)	28 (68)	38 (38)	186 (74)	46 (58)	
Age (years)	64.7 (13.1)	65.4 (13.5)	65.4 (13.1)	68.9 (14.4)	72.7 (12.4)	<.001
BMI (kg/m^2^)	30.1 (6.8)	27.0 (4.6)	27.4 (5.9)	26.0 (5.7)	25.1 (5.7)	<.001
Education						.83
High school	95 (22)	11 (27)	19 (19)	51 (20)	18 (24)	
Some college	181 (42)	13 (32)	42 (42)	92 (37)	30 (38)	
College graduate	146 (34)	16 (39)	37 (37)	106 (42)	30 (38)	
Smoking (ever)	105 (24)	5 (12)	14 (14)	30 (12)	8 (10)	<.001
Alcohol (current/former)	223 (52)	16 (39)	34 (34)	66 (26)	22 (28)	<.001
Prevalent disease						
Hypertension	103 (24)	12 (29)	20 (20)	51 (20)	8 (10)	.05
Diabetes	61 (14)	2 (5)	8 (8)	25 (10)	3 (4)	.02
Cardiovascular	28 (7)	2 (5)	2 (2)	8 (3)	4 (5)	.2
Cancer	26 (6)	2 (5)	6 (6)	16 (6)	6 (8)	.99
CKD stage ≥2	242 (53.9)	23 (58)	43 (46)	132 (55)	49 (62)	.43
CKD stage ≥3	36 (8.4)	5 (12.2)	4 (4.0)	22 (8.8)	5 (6.3)	.45
Creatinine (mg/dL)	0.86 (0.27)	0.88 (0.27)	0.81 (0.18)	0.87 (0.47)	0.79 (0.22)	.23
eGFR (mL/min/1.73 m^2^)	85.7 (17.3)	83.4 (20.6)	87.6 (14.7)	84.8 (18.3)	84.7 (16.6)	.62
Total energy (kcal/d)	1,936.7 (816.7)	1,575.8 (650.2)	2,012.9 (847.8)	1,961.8 (757.2)	1,853.5 (709.8)	.045

AHS, Adventist Health Study; BMI, body mass index; CKD, chronic kidney disease; eGFR, estimated glomerular filtration rate; SD, standard deviation.

* Values presented as n (%) or mean (SD); missing values: BMI n = 24, alcohol n = 13, education n = 12, cancer n = 104, CVD = 109.

**Table 2.** Associations of Dietary Pattern With eGFR in AHS-2 Participants

	Dietary Pattern	Mean (95% CI)	*β* (SE)	*P* Value
Model 1*	Vegan	89.5 (86.3, 92.7)	4.6 (1.8)	.01
	Lacto-ovo-vegetarian	85.4 (83.6, 87.3)	0.5 (1.2)	.64
	Pesco-vegetarian	87.8 (85.0, 90.6)	2.9 (1.6)	.067
	Semivegetarian	81.8 (77.5, 86.2)	−3.0 (2.3)	.19
	Nonvegetarian	84.9 (83.5, 86.3)	(Reference)	
	All vegetarian†	86.6 (85.2, 88.0)	1.9 (1.0)	.059
	Nonvegetarian	84.8 (83.4, 86.2)		
Model 2‡	Vegan	90.6 (87.2, 94.0)	4.8 (1.8)	.008
	Lacto-ovo-vegetarian	86.4 (84.3, 88.5)	0.6 (1.2)	.64
	Pesco-vegetarian	89.0 (86.0, 92.1)	3.2 (1.6)	.05
	Semivegetarian	83.0 (78.5, 87.6)	−2.8 (2.4)	.24
	Nonvegetarian	85.8 (84.2, 87.4)	(Reference)	
	All vegetarian†	87.6 (85.8, 89.3)	2.1 (1.0)	.046
	Nonvegetarian	85.5 (83.9, 87.1)	(Reference)	
Model 3§	Vegan	90.2 (86.7, 93.6)	3.7 (1.9)	.047
	Lacto-ovo-vegetarian	86.5 (84.3, 88.7)	0.0 (1.3)	.99
	Pesco-vegetarian	89.2 (86.1, 92.3)	2.7 (1.7)	.1
	Semivegetarian	83.6 (79.0, 88.2)	−2.9 (2.4)	.24
	Nonvegetarian	86.5 (84.8, 88.1)	(Reference)	
	All vegetarian†	87.6 (85.9, 89.4)	1.4 (1.1)	.18
	Nonvegetarian	86.2 (84.5, 87.8)	(Reference)	
Model 4¶	Vegan	91.0 (86.1, 96.0)	4.0 (2.0)	.043
	Lacto-ovo-vegetarian	87.8 (83.9, 91.7)	0.8 (1.4)	.53
	Pesco-vegetarian	90.6 (86.1, 95.1)	3.6 (1.8)	.038
	Semivegetarian	85.5 (79.7, 91.3)	−1.4 (2.6)	.58
	Nonvegetarian	87.0 (83.6, 90.3)	(Reference)	
	All vegetarian†	88.1 (84.4, 91.9)	2.1 (1.2)	.07
	Nonvegetarian	86.0 (82.6, 89.4)	(Reference)	

AHS, Adventist Health Study; CI, confidence interval; eGFR, estimated glomerular filtration rate; SE, standard error.

* Model 1 adjusted for race, gender, and age at creatinine measurement, creatinine batch.

† All vegetarian category excludes semivegetarians.

‡ Model 2 includes adjustment for variables in model 1, with additional adjustment for education level, smoking status, and alcohol intake.

§ Model 3 includes variables in model 2, in addition to body mass index (BMI).

¶ Model 4 includes variables in model 3, with further adjustment for diabetes, hypertension, cardiovascular disease (CVD), and cancer history.

**Table 3.** Metabolite Subclasses Associated With Serum Creatinine at FDR < 0.05*

Subclass Labels	Fold Change (95% CI)	n Total Metabolites	n Significant Metabolites	n Significant >1	n Significant <1	FDR
Guanidino and acetamido metabolism	1.91 (1.59, 2.29)	2	2	2	0	<8.6 × 10^−5^
Histidine metabolism	1.55 (1.37, 1.75)	15	11	11	0	<8.6 × 10^−5^
Food component/plant	1.45 (1.27, 1.66)	52	22	22	0	<8.6 × 10^−5^
Acetylated peptides	1.42 (1.02, 1.98)	3	1	1	0	1.9 × 10^−2^
Vitamin B6 metabolism	1.40 (1.07, 1.84)	2	1	1	0	1.1 × 10^−2^
Fatty acid metabolism (acyl carnitine, hydroxy)	1.38 (1.10, 1.72)	5	3	3	0	5.6 × 10^−3^
Pantothenate and coenzyme A metabolism	1.38 (1.09, 1.74)	2	1	1	0	6.5 × 10^−3^
Benzoate metabolism	1.36 (1.10, 1.68)	24	9	9	0	5.0 × 10^−3^
Lysine metabolism	1.35 (1.22, 1.50)	16	8	8	0	<8.6 × 10^−5^
Chemical	1.33 (1.16, 1.51)	20	7	7	0	4.0 × 10^−4^
Aminosugar metabolism	1.32 (1.20, 1.44)	5	5	5	0	<8.6 × 10^−5^
Fatty acid metabolism (acyl carnitine, dicarboxylate)	1.31 (1.10, 1.58)	4	2	2	0	4.6 × 10^−3^
Fatty acid metabolism (acyl glycine)	1.30 (1.09, 1.56)	7	4	4	0	5.1 × 10^−3^
Nicotinate and nicotinamide metabolism	1.29 (1.12, 1.49)	5	3	3	0	1.3 × 10^−3^
Drug - topical agents	1.29 (0.98, 1.71)	3	0	0	0	3.0 × 10^−2^
Urea cycle; arginine and proline metabolism	1.27 (1.17, 1.38)	20	11	11	0	<8.6 × 10^−5^
Phospholipid metabolism	1.26 (1.15, 1.37)	6	5	5	0	9.8 × 10^−5^
Pentose metabolism	1.25 (1.10, 1.43)	6	4	4	0	1.5 × 10^−3^
Ceramides	1.25 (1.06, 1.47)	9	7	7	0	6.4 × 10^−3^
Phenylalanine metabolism	1.24 (1.11, 1.39)	6	3	3	0	8.0 × 10^−4^
Ascorbate and aldarate metabolism	1.24 (1.10, 1.40)	6	3	3	0	1.6 × 10^−3^
Fatty acid metabolism (also BCAA metabolism)	1.24 (1.04, 1.48)	4	1	1	0	1.2 × 10^−2^
Pyrimidine metabolism, orotate containing	1.23 (1.06, 1.43)	3	1	1	0	5.8 × 10^−3^
Fructose, mannose and galactose metabolism	1.23 (1.04, 1.47)	4	1	1	0	1.2 × 10^−2^
Tryptophan metabolism	1.22 (1.11, 1.34)	20	11	11	0	4.7 × 10^−4^
Partially characterized molecules	1.22 (1.02, 1.46)	13	6	6	0	1.7 × 10^−2^
Tyrosine metabolism	1.21 (1.11, 1.33)	21	10	10	0	4.4 × 10^−4^
Polyamine metabolism	1.21 (1.11, 1.31)	7	5	5	0	2.4 × 10^−4^
Leucine, isoleucine and valine metabolism	1.21 (1.11, 1.31)	28	15	15	0	3.7 × 10^−4^
Plasmalogen	1.21 (1.09, 1.34)	11	9	9	0	1.1 × 10^−3^
Methionine, cysteine, SAM, and taurine metabolism	1.20 (1.11, 1.30)	24	12	12	0	2.7 × 10^−4^
Fatty acid metabolism (acyl carnitine, long chain Saturated)	1.20 (1.04, 1.38)	8	4	4	0	1.1 × 10^−2^
Phosphatidylethanolamine (PE)	1.20 (1.01, 1.41)	12	3	3	0	1.8 × 10^−2^
Fatty acid, amino	1.19 (1.01, 1.41)	3	1	1	0	2.0 × 10^−2^
Purine metabolism, adenine containing	1.18 (1.10, 1.26)	5	3	3	0	8.6 × 10^−5^
Lysoplasmalogen	1.18 (1.05, 1.33)	4	2	2	0	5.8 × 10^−3^
Fatty acid metabolism (acyl carnitine, medium chain)	1.18 (0.97, 1.44)	5	3	3	0	4.1 × 10^−2^
Fatty acid metabolism (acyl carnitine, monounsaturated)	1.17 (1.02, 1.34)	9	3	3	0	1.5 × 10^−2^
Sphingomyelins	1.16 (1.07, 1.27)	29	24	24	0	1.6 × 10^−3^
Pyrimidine metabolism, cytidine containing	1.16 (1.03, 1.31)	5	1	1	0	1.1 × 10^−2^
Pyrimidine metabolism, thymine containing	1.16 (1.00, 1.36)	2	1	1	0	2.6 × 10^−2^
Alanine and aspartate metabolism	1.15 (1.08, 1.24)	9	5	5	0	5.0 × 10^−4^
Fatty acid, dihydroxy	1.15 (1.03, 1.29)	5	3	3	0	1.2 × 10^−2^
Purine metabolism, guanine containing	1.15 (1.02, 1.29)	3	2	2	0	1.5 × 10^−2^
Fatty acid, dicarboxylate	1.14 (1.01, 1.29)	33	6	6	0	1.9 × 10^−2^
Vitamin A metabolism	1.14 (0.96, 1.35)	5	1	1	0	4.7 × 10^−2^
Pyrimidine metabolism, uracil containing	1.12 (1.03, 1.22)	12	4	4	0	8.7 × 10^−3^
Lysophospholipid	1.12 (1.01, 1.25)	29	11	11	0	1.8 × 10^−2^
Sterol	1.12 (0.99, 1.27)	6	3	3	0	3.3 × 10^−2^
Glycerolipid metabolism	1.12 (0.99, 1.27)	2	1	1	0	3.5 × 10^−2^
Glutamate metabolism	1.06 (0.98, 1.15)	10	2	2	0	4.9 × 10^−2^
Creatine metabolism	1.06 (0.98, 1.14)	3	1	1	0	4.8 × 10^−2^
Pregnenolone steroids	0.67 (0.54, 0.84)	7	0	0	0	4.1 × 10^−2^

BCAA, branched chain amino acid; BMI, body mass index; CI, confidence interval; FDR, false discovery rate; SAM, S-adenosylmethionine.

* Adjustment for race, BMI, sex, age, batch, study, and batch*study.

**Table 4.** Metabolite Subclasses Associated With Both Creatinine and Vegan (Relative to Nonvegetarian) Dietary Pattern at FDR < 0.05*

Subclass	Vegan, Fold Change (95% CI)	FDR	Creatinine, Fold Change (95% CI)	FDR
Acetylated peptides	0.33 (0.21, 0.52)	9.9 × 10^−5^	1.42 (1.02, 1.98)	1.9 × 10^−2^
Histidine metabolism	0.49 (0.41, 0.58)	5.0 × 10^−4^	1.55 (1.37, 1.75)	<3.7 × 10^−4^
Ceramides	0.53 (0.42, 0.68)	1.2 × 10^−4^	1.25 (1.06, 1.47)	6.4 × 10^−3^
Fatty acid metabolism (also BCAA metabolism)	0.67 (0.52, 0.86)	4.0 × 10^−3^	1.24 (1.04, 1.48)	1.2 × 10^−2^
Fatty acid metabolism (acyl carnitine, long chain saturated)	0.67 (0.54, 0.83)	1.1 × 10^−3^	1.20 (1.04, 1.38)	1.1 × 10^−2^
Lysoplasmalogen	0.68 (0.56, 0.82)	2.5 × 10^−4^	1.18 (1.05, 1.33)	5.8 × 10^−3^
Plasmalogen	0.68 (0.57, 0.82)	2.8 × 10^−4^	1.21 (1.09, 1.34)	1.1 × 10^−3^
Fatty acid metabolism (acyl carnitine, medium chain)	0.70 (0.52, 0.94)	3.1 × 10^−2^	1.18 (0.97, 1.44)	4.1 × 10^−2^
Phenylalanine metabolism	0.71 (0.59, 0.86)	1.8 × 10^−3^	1.24 (1.11, 1.39)	8.0 × 10^−4^
Chemical	0.73 (0.58, 0.93)	1.9 × 10^−2^	1.33 (1.16, 1.51)	4.0 × 10^−4^
Lysine metabolism	0.73 (0.63, 0.85)	2.8 × 10^−4^	1.35 (1.22, 1.50)	<3.7 × 10^−4^
Leucine, isoleucine, and valine metabolism	0.77 (0.66, 0.90)	3.3 × 10^−3^	1.21 (1.11, 1.31)	3.7 × 10^−4^
Fatty acid metabolism (acyl glycine)	1.63 (1.26, 2.11)	4.2 × 10^−3^	1.30 (1.09, 1.56)	5.1 × 10^−3^
Vitamin A metabolism	1.76 (1.37, 2.27)	<1.0 × 10^−4^	1.14 (0.96, 1.35)	4.7 × 10^−2^

BCAA, branched chain amino acid; BMI, body mass index; CI, confidence interval; FDR, false discovery rate.

* Adjustment for race, BMI, sex, age, batch, study, and batch*study.

**Table 5.** Metabolite Subclasses Associated With Both Creatinine and Pesco-vegetarian (Relative to Nonvegetarian) Dietary Pattern at FDR < 0.05*

Subclass Labels	Pesco-Vegetarian, Fold Change (95% CI)	FDR	Creatinine, Fold Change (95% CI)	FDR
Plasmalogen	0.53 (0.43, 0.64)	2.7 × 10^−4^	1.21 (1.09, 1.34)	1.1 × 10^−3^
Histidine metabolism	0.54 (0.46, 0.65)	5.4 × 10^−4^	1.55 (1.37, 1.75)	<1.0 × 10^−4^
Acetylated peptides	0.56 (0.34, 0.90)	4.3 × 10^−2^	1.42 (1.02, 1.98)	1.9 × 10^−2^
Pyrimidine metabolism, orotate containing	0.58 (0.36, 0.92)	4.8 × 10^−2^	1.23 (1.06, 1.43)	5.8 × 10^−3^
Lysoplasmalogen	0.60 (0.50, 0.74)	1.8 × 10^−4^	1.18 (1.05, 1.33)	5.8 × 10^−3^
Sphingomyelins	0.65 (0.53, 0.80)	5.4 × 10^−4^	1.16 (1.07, 1.27)	1.6 × 10^−3^
Pentose metabolism	0.67 (0.49, 0.93)	4.4 × 10^−2^	1.25 (1.10, 1.43)	1.5 × 10^−3^
Phospholipid metabolism	0.67 (0.54, 0.83)	1.6 × 10^−3^	1.26 (1.15, 1.37)	9.8 × 10^−5^
Purine metabolism, guanine containing	0.74 (0.62, 0.89)	7.1 × 10^−3^	1.15 (1.02, 1.29)	1.5 × 10^−2^
Lysine metabolism	0.78 (0.67, 0.92)	1.3 × 10^−2^	1.35 (1.22, 1.50)	<1.0 × 10^−4^
Aminosugar metabolism	0.80 (0.66, 0.96)	4.5 × 10^−2^	1.32 (1.20, 1.44)	<1.0 × 10^−4^

BMI, body mass index; CI, confidence interval; FDR, false discovery rate.

* Adjustment for race, BMI, sex, age, batch, study, and batch*study.

## Practical Application

This research highlights the potential of plant-based diets, particularly vegan and pesco-vegetarian patterns, to promote kidney function through favorable metabolic effects. These findings have meaningful practical implications supporting the integration of plant-based dietary approaches into CKD prevention and management strategies, offering cost-effective and accessible interventions to modulate key metabolic pathways and enhance kidney health.
