# Supplementary material for: Vegetarian Dietary Patterns and Diet-Related Metabolites Are Associated With Kidney Function in the Adventist Health Study-2 Cohort
Source: J Ren Nutr. Author manuscript; Available in PMC 2026 Jul 17. (PMC13379062; doi:10.1053/j.jrn.2025.11.001)
Supplement: 1 [file NIHMS2188991-supplement-1.docx]

| **Supplementary Table 1**. Linear regression associations of dietary pattern, race, sex, and age with eGFR in participants aged > 65 years | | | | | | | | |
| --- | --- | --- | --- | --- | --- | --- | --- | --- |
|  | **Model 1 β (SE)^1^** | **P** | **Model 2 β (SE)^2^** | **P** | **Model 3 β (SE)^3^** | **P** | **Model 4 β (SE)^4^** | **P** |
| **Dietary pattern** |  |  |  |  |  |  |  |  |
| Semi-vegetarian | -4.3 (3.2) | 0.18 | -3.7 (3.3) | 0.26 | -3.2 (3.4) | 0.35 | -1.2 (3.6) | 0.74 |
| Pesco-vegetarian | 4.5 (2.2) | 0.043 | 5.1 (2.3) | 0.026 | 4.4 (2.3) | 0.056 | 4.3 (2.5) | 0.08 |
| Lacto-ovo-vegetarian | 0.6 (1.6) | 0.72 | 1.2 (1.7) | 0.49 | 0.5 (1.7) | 0.76 | 1.1 (1.9) | 0.57 |
| Vegan | 3.9 (2.2) | 0.075 | 4.5 (2.3) | 0.047 | 3.1 (2.3) | 0.18 | 2.6 (2.5) | 0.3 |
| **Black vs non-Black** | -5.9 (1.4) | 3.7e-05 | -6.0 (1.5) | 5.6e-05 | -5.4 (1.5) | 3.0e-04 | -4.8 (1.6) | 0.004 |
| **Female vs male** | -3.1 (1.4) | 0.024 | -2.9 (1.5) | 0.048 | -2.9 (1.5) | 0.048 | -1.9 (1.6) | 0.24 |
| **Age** | -0.8 (0.1) | 2.0e-16 | -0.8 (0.1) | 2.0e-16 | -0.9 (0.1) | 2.0e-16 | -0.9 (0.1) | 2.0e-16 |
| ^1^Model 1 adjusted for race, gender, education, smoking, alcohol drinking, age at creatinine measurement, creatinine batch | | | | | | | | |
| ^2^Model 2 includes variables in Model 1, with additional adjustment for education level, smoking status, and alcohol intake. | | | | | | | | |
| ^3^Model 3 includes variables in Model 2, in addition to body mass index (BMI). | | | | |  |  |  |  |
| ^4^Model 4 includes variables in Model 3, with further adjustment for diabetes, hypertension, cardiovascular disease (CVD), and cancer history. | | | | | | | | |

| **Supplementary Table 2**. Linear regression associations of dietary pattern, race, sex, and age with eGFR in participants aged < 65 years | | | | | | | | |
| --- | --- | --- | --- | --- | --- | --- | --- | --- |
|  | **Model 1 β (SE)^1^** | **P** | **Model 2 β (SE)^2^** | **P** | **Model 3 β (SE)^3^** | **P** | **Model 4 β (SE)^4^** | **P** |
| **Dietary pattern** |  |  |  |  |  |  |  |  |
| Semi-vegetarian | -1.5 (3.5) | 0.67 | -1.6 (3.5) | 0.64 | -2.1 (3.5) | 0.55 | -1.6 (3.8) | 0.67 |
| Pesco-vegetarian | 1.2 (2.3) | 0.6 | 1.2 (2.4) | 0.61 | 1.1 (2.5) | 0.66 | 3.2 (2.5) | 0.21 |
| Lacto-ovo-vegetarian | 0.6 (1.7) | 0.71 | 0.0 (1.8) | 0.98 | -0.6 (1.9) | 0.73 | 1.0 (2.0) | 0.62 |
| Vegan | 6.3 (3.1) | 0.041 | 5.6 (3.1) | 0.069 | 4.8 (3.2) | 0.14 | 5.9 (3.4) | 0.08 |
| **Black vs non-Black** | -5.7 (1.5) | 0.0001 | -5.5 (1.5) | 0.0003 | -5.4 (1.5) | 0.0005 | -5.6 (1.6) | 0.0006 |
| **Female vs male** | -2.0 (1.5) | 0.2 | -2.2 (1.6) | 0.17 | -2.3 (1.6) | 0.14 | -2.9 (1.6) | 0.08 |
| **Age** | -0.7 (0.1) | 4.7E-11 | -0.7 (0.1) | 6.0E-11 | -0.7 (0.1) | 1.1E-09 | -0.7 (0.1) | 1.6E-08 |
| ^1^Model 1 adjusted for race, gender, education, smoking, alcohol drinking, age at creatinine measurement, creatinine batch | | | | | | | | |
| ^2^Model 2 includes variables in Model 1, with additional adjustment for education level, smoking status, and alcohol intake. | | | | | | | | |
| ^3^Model 3 includes variables in Model 2, in addition to body mass index (BMI). | | | | |  |  |  |  |
| ^4^Model 4 includes variables in Model 3, with further adjustment for diabetes, hypertension, cardiovascular disease (CVD), and cancer history. | | | | | | | | |

| **Supplementary Table 3**. Linear regression associations between dietary pattern and eGFR in Black female participants | | | | | | | |  |
| --- | --- | --- | --- | --- | --- | --- | --- | --- |
| Dietary pattern | **Model 1 β (SE)¹** | **P** | **Model 2 β (SE)²** | **P** | **Model 3 β (SE)³** | **P** | **Model 4 β (SE)⁴** | **P** |
| Semi-vegetarian | 0.9 (5.5) | 0.87 | -2.0 (5.6) | 0.72 | -1.5 (5.6) | 0.79 | 0.1 (6.0) | 0.98 |
| Pesco-vegetarian | -1.3 (2.7) | 0.63 | -2.5 (2.8) | 0.37 | -2.0 (2.9) | 0.49 | -2.0 (3.1) | 0.52 |
| Lacto-ovo-vegetarian | 1.1 (2.7) | 0.69 | 1.3 (2.9) | 0.65 | 1.9 (2.9) | 0.53 | 1.1 (3.1) | 0.71 |
| Vegan | -0.7 (3.3) | 0.83 | -1.6 (3.5) | 0.65 | -1.4 (3.6) | 0.69 | -0.5 (4.0) | 0.9 |
| ^1^Model 1 adjusted for gender, education, smoking, alcohol drinking, age at creatinine measurement, creatinine batch | | | | | | | | |
| ^2^Model 2 includes variables in Model 1, with additional adjustment for education level, smoking status, and alcohol intake. | | | | | | | | |
| ^3^Model 3 includes variables in Model 2, in addition to body mass index (BMI). | | | | |  |  |  |  |
| ^4^Model 4 includes variables in Model 3, with further adjustment for diabetes, hypertension, cardiovascular disease (CVD), and cancer history. | | | | | | | | |

| **Supplementary Table 4**. Linear regression associations between dietary pattern and eGFR in Black male participants | | | | | | | |  |
| --- | --- | --- | --- | --- | --- | --- | --- | --- |
| **Dietary pattern** | **Model 1 β (SE)¹** | **P** | **Model 2 β (SE)²** | **P** | **Model 3 β (SE)³** | **P** | **Model 4 β (SE)⁴** | **P** |
| Semi-vegetarian | -10.8 (6.5) | 0.1 | -11.4 (6.7) | 0.092 | -10.7 (7.3) | 0.15 | -9.3 (8.0) | 0.25 |
| Pesco-vegetarian | 2.9 (3.6) | 0.41 | 4.0 (3.9) | 0.31 | 1.4 (3.9) | 0.72 | 4.2 (4.1) | 0.31 |
| Lacto-ovo-vegetarian | -0.8 (3.2) | 0.8 | -1.3 (3.5) | 0.7 | -3.0 (3.4) | 0.38 | -1.7 (3.6) | 0.64 |
| Vegan | 9.4 (5.6) | 0.096 | 10.8 (5.9) | 0.071 | 7.4 (5.8) | 0.21 | 9.0 (5.8) | 0.12 |
| ^1^Model 1 adjusted for gender, education, smoking, alcohol drinking, age at creatinine measurement, creatinine batch | | | | | | | | |
| ^2^Model 2 includes variables in Model 1, with additional adjustment for education level, smoking status, and alcohol intake. | | | | | | | | |
| ^3^Model 3 includes variables in Model 2, in addition to body mass index (BMI). | | | | |  |  |  |  |
| ^4^Model 4 includes variables in Model 3, with further adjustment for diabetes, hypertension, cardiovascular disease (CVD), and cancer history. | | | | | | | | |

| **Supplementary Table 5.** Linear regression associations between dietary pattern and eGFR in White female participants | | | | | | | |  |
| --- | --- | --- | --- | --- | --- | --- | --- | --- |
| Dietary pattern | **Model 1 β (SE)¹** | **P** | **Model 2 β (SE)²** | **P** | **Model 3 β (SE)³** | **P** | **Model 4 β (SE)⁴** | **P** |
| Semi-vegetarian | -5.6 (3.4) | 0.1 | -5.0 (3.6) | 0.17 | -6.8 (3.5) | 0.052 | -4.7 (3.7) | 0.2 |
| Pesco-vegetarian | 8.2 (2.9) | 0.005 | 8.3 (2.9) | 0.005 | 6.3 (3.0) | 0.033 | 8.6 (3.1) | 0.005 |
| Lacto-ovo-vegetarian | 1.4 (1.8) | 0.44 | 1.6 (1.9) | 0.4 | -1.2 (1.9) | 0.53 | 0.7 (2.0) | 0.73 |
| Vegan | 7.3 (2.8) | 0.01 | 7.6 (2.8) | 0.008 | 4.4 (2.9) | 0.12 | 4.8 (3.1) | 0.12 |
| ^1^Model 1 adjusted for gender, education, smoking, alcohol drinking, age at creatinine measurement, creatinine batch | | | | | | | | |
| ^2^Model 2 includes variables in Model 1, with additional adjustment for education level, smoking status, and alcohol intake. | | | | | | | | |
| ^3^Model 3 includes variables in Model 2, in addition to body mass index (BMI). | | | | |  |  |  |  |
| ^4^Model 4 includes variables in Model 3, with further adjustment for diabetes, hypertension, cardiovascular disease (CVD), and cancer history. | | | | | | | | |

| **Supplementary Table 6**. Linear regression associations between dietary pattern and eGFR in White male participants | | | | | | | |  |
| --- | --- | --- | --- | --- | --- | --- | --- | --- |
| **Dietary Pattern** | **Model 1 β (SE)¹** | **P** | **Model 2 β (SE)²** | **P** | **Model 3 β (SE)³** | **P** | **Model 4 β (SE)⁴** | **P** |
| Semi-vegetarian | 6.42 (4.71) | 0.175 | 6.17 (4.84) | 0.204 | 5.87 (4.83) | 0.226 | 4.74 (5.43) | 0.384 |
| Pesco-vegetarian | 6.62 (4.47) | 0.14 | 6.79 (4.60) | 0.142 | 6.37 (4.56) | 0.164 | 5.50 (5.24) | 0.295 |
| Lacto-ovo-vegetarian | 1.96 (2.20) | 0.374 | 2.27 (2.36) | 0.337 | 3.16 (2.47) | 0.202 | 4.32 (2.86) | 0.133 |
| Vegan | 9.86 (3.92) | 0.013 | 9.56 (4.00) | 0.018 | 8.97 (4.12) | 0.031 | 8.47 (4.62) | 0.069 |
| ^1^Model 1 adjusted for gender, education, smoking, alcohol drinking, age at creatinine measurement, creatinine batch | | | | | | | | |
| ^2^Model 2 includes variables in Model 1, with additional adjustment for education level, smoking status, and alcohol intake. | | | | | | | | |
| ^3^Model 3 includes variables in Model 2, in addition to body mass index (BMI). | | | | |  |  |  |  |
| ^4^Model 4 includes variables in Model 3, with further adjustment for diabetes, hypertension, cardiovascular disease (CVD), and cancer history. | | | | | | | | |

| **Supplementary Table 7.** Associations of plasma metabolites with plasma creatinine at FDR < 0.05 in AHS-2 participants^1,2^ | | | |
| --- | --- | --- | --- |
| **Metabolite** | **Subclass** | **Fold Change** | **FDR** |
| piperine | Food Component/Plant | 5.10 | 8.4E-03 |
| 1-methyl-5-imidazoleacetate | Histidine Metabolism | 4.29 | 7.9E-04 |
| glucuronide of piperine metabolite c17h21no3 (4) | Food Component/Plant | 4.02 | 2.1E-03 |
| 3-methylhistidine | Histidine Metabolism | 3.96 | 3.2E-03 |
| acesulfame | Food Component/Plant | 3.87 | 3.9E-02 |
| n-acetylalliin | Food Component/Plant | 3.86 | 4.1E-03 |
| 3-carboxy-4-methyl-5-propyl-2-furanpropanoate (cmpf) | Fatty Acid, Dicarboxylate | 3.72 | 2.8E-02 |
| eugenol sulfate | Food Component/Plant | 3.55 | 1.7E-03 |
| glucuronide of piperine metabolite c17h21no3 (3) | Food Component/Plant | 3.48 | 2.2E-03 |
| glucuronide of piperine metabolite c17h21no3 (5) | Food Component/Plant | 3.39 | 2.0E-03 |
| n-acetyl-1-methylhistidine | Histidine Metabolism | 3.35 | 6.0E-04 |
| sulfate of piperine metabolite c16h19no3 (3) | Food Component/Plant | 3.30 | 3.8E-03 |
| quinate | Food Component/Plant | 3.18 | 4.9E-02 |
| guanidinosuccinate | Guanidino and Acetamido Metabolism | 3.12 | <3.4E-4 |
| sulfate of piperine metabolite c18h21no3 (1) | Food Component/Plant | 3.08 | 8.6E-03 |
| 3-methoxycatechol sulfate (1) | Benzoate Metabolism | 3.07 | 2.6E-02 |
| sulfate of piperine metabolite c16h19no3 (2) | Food Component/Plant | 3.04 | 2.7E-03 |
| 3-methyl catechol sulfate (1) | Benzoate Metabolism | 2.87 | 2.6E-03 |
| ectoine | Chemical | 2.79 | 9.8E-04 |
| p-cresol glucuronide | Tyrosine Metabolism | 2.74 | 4.2E-02 |
| sulfate of piperine metabolite c18h21no3 (3) | Food Component/Plant | 2.72 | 9.2E-03 |
| glutamine conjugate of c7h12o2 | Partially Characterized Molecules | 2.71 | 8.9E-03 |
| 4-methylguaiacol sulfate | Benzoate Metabolism | 2.70 | 3.3E-03 |
| methyl-4-hydroxybenzoate sulfate | Benzoate Metabolism | 2.70 | 2.4E-02 |
| salicyluric glucuronide | Drug - Analgesics, Anesthetics | 2.66 | 3.9E-02 |
| (2,4 or 2,5)-dimethylphenol sulfate | Food Component/Plant | 2.42 | 4.2E-03 |
| o-cresol sulfate | Benzoate Metabolism | 2.42 | 1.6E-03 |
| 5-hydroxy-2-methylpyridine sulfate | Chemical | 2.35 | 6.9E-03 |
| indoleacetylglutamine | Tryptophan Metabolism | 2.33 | 1.5E-03 |
| tyramine o-sulfate | Tyrosine Metabolism | 2.28 | 4.5E-03 |
| 3-methylglutarylcarnitine (2) | Leucine, Isoleucine and Valine Metabolism | 2.26 | 3.7E-04 |
| homocitrulline | Urea cycle; Arginine and Proline Metabolism | 2.23 | <3.4E-04 |
| heptenedioate (c7:1-dc) | Fatty Acid, Dicarboxylate | 2.22 | 1.3E-03 |
| 4-allylcatechol sulfate | Benzoate Metabolism | 2.19 | 1.8E-02 |
| phenylacetylglutamate | Acetylated Peptides | 2.18 | 4.6E-03 |
| 2,3-dihydroxyisovalerate | Food Component/Plant | 2.08 | 2.5E-03 |
| methyl indole-3-acetate | Food Component/Plant | 2.04 | 6.3E-04 |
| levulinoylcarnitine | Food Component/Plant | 2.03 | 1.2E-02 |
| lanthionine | Methionine, Cysteine, SAM and Taurine Metabolism | 2.02 | <3.4E-04 |
| ethyl alpha-glucopyranoside | Food Component/Plant | 1.98 | 2.5E-02 |
| n-acetyltaurine | Methionine, Cysteine, SAM and Taurine Metabolism | 1.97 | <3.4E-04 |
| 2-hydroxyphenylacetate | Phenylalanine Metabolism | 1.96 | 5.6E-04 |
| n-methylpipecolate | Bacterial/Fungal | 1.95 | 1.7E-03 |
| tridecenedioate (c13:1-dc) | Fatty Acid, Dicarboxylate | 1.95 | 6.8E-03 |
| n2-acetyl,n6,n6-dimethyllysine | Lysine Metabolism | 1.95 | 3.9E-03 |
| hydantoin-5-propionate | Histidine Metabolism | 1.94 | 1.4E-03 |
| n-acetylcitrulline | Urea cycle; Arginine and Proline Metabolism | 1.90 | 2.1E-03 |
| n,n-dimethyl-5-aminovalerate | Lysine Metabolism | 1.90 | 2.7E-02 |
| glycine conjugate of c10h14o2 (1) | Partially Characterized Molecules | 1.86 | 1.5E-03 |
| glycine conjugate of c10h12o2 | Partially Characterized Molecules | 1.85 | 1.4E-03 |
| pyridoxate | Vitamin B6 Metabolism | 1.85 | 8.4E-03 |
| p-cresol sulfate | Benzoate Metabolism | 1.84 | 1.1E-02 |
| 4-methylcatechol sulfate | Benzoate Metabolism | 1.84 | 4.3E-03 |
| 1-methylhistidine | Histidine Metabolism | 1.83 | <3.4E-04 |
| pantoate | Pantothenate and CoA Metabolism | 1.83 | 6.1E-03 |
| 5alpha-androstan-3beta,17alpha-diol disulfate | Androgenic Steroids | 1.82 | 4.6E-02 |
| 3-hydroxy-2-methylpyridine sulfate | Chemical | 1.81 | 5.0E-03 |
| n6,n6,n6-trimethyllysine | Lysine Metabolism | 1.81 | <3.4E-04 |
| trigonelline (n'-methylnicotinate) | Nicotinate and Nicotinamide Metabolism | 1.80 | 7.0E-03 |
| 3-hydroxypyridine sulfate | Chemical | 1.80 | 3.3E-02 |
| 2-butenoylglycine | Fatty Acid Metabolism (Acyl Glycine) | 1.80 | 4.0E-02 |
| cytosine | Pyrimidine Metabolism, Cytidine containing | 1.79 | 1.6E-02 |
| urea | Urea cycle; Arginine and Proline Metabolism | 1.77 | <3.4E-04 |
| n2,n5-diacetylornithine | Urea cycle; Arginine and Proline Metabolism | 1.77 | 4.1E-04 |
| n,n,n-trimethyl-5-aminovalerate | Lysine Metabolism | 1.75 | 6.1E-03 |
| 8-methoxykynurenate | Tryptophan Metabolism | 1.75 | 1.4E-03 |
| delta-cehc glucuronide | Tocopherol Metabolism | 1.72 | 5.0E-03 |
| cystathionine | Methionine, Cysteine, SAM and Taurine Metabolism | 1.71 | 4.9E-02 |
| xanthurenate | Tryptophan Metabolism | 1.70 | 2.0E-03 |
| gulonate | Ascorbate and Aldarate Metabolism | 1.70 | <3.4E-04 |
| 6-hydroxyindole sulfate | Chemical | 1.69 | 5.0E-03 |
| undecenoylcarnitine (c11:1) | Fatty Acid Metabolism (Acyl Carnitine, Monounsaturated) | 1.68 | 1.4E-03 |
| etiocholanolone glucuronide | Androgenic Steroids | 1.67 | 4.6E-03 |
| pimeloylcarnitine/3-methyladipoylcarnitine (c7-dc) | Fatty Acid Metabolism (Acyl Carnitine, Dicarboxylate) | 1.67 | 3.9E-03 |
| tiglylcarnitine (c5:1-dc) | Leucine, Isoleucine and Valine Metabolism | 1.66 | 6.2E-04 |
| trimethylamine n-oxide | Phospholipid Metabolism | 1.66 | 6.5E-03 |
| beta-sitosterol | Sterol | 1.65 | 1.9E-02 |
| guaiacol sulfate | Benzoate Metabolism | 1.65 | 7.0E-03 |
| 2s,3r-dihydroxybutyrate | Fatty Acid, Dihydroxy | 1.64 | 3.9E-04 |
| 3-methylglutaconate | Leucine, Isoleucine and Valine Metabolism | 1.63 | 1.3E-03 |
| glutamine conjugate of c6h10o2 (1) | Partially Characterized Molecules | 1.63 | 1.0E-02 |
| beta-hydroxyisovaleroylcarnitine | Leucine, Isoleucine and Valine Metabolism | 1.62 | 4.2E-04 |
| glycerophosphoinositol | Phospholipid Metabolism | 1.62 | 1.2E-02 |
| picolinoylglycine | Fatty Acid Metabolism (Acyl Glycine) | 1.61 | 9.4E-04 |
| n1-methyl-2-pyridone-5-carboxamide | Nicotinate and Nicotinamide Metabolism | 1.60 | 4.2E-03 |
| isobutyrylcarnitine (c4) | Leucine, Isoleucine and Valine Metabolism | 1.60 | 5.2E-03 |
| (s)-3-hydroxybutyrylcarnitine | Fatty Acid Metabolism (Acyl Carnitine, Hydroxy) | 1.58 | 4.6E-03 |
| glutarylcarnitine (c5-dc) | Lysine Metabolism | 1.57 | 4.2E-03 |
| trans-2-hexenoylglycine | Fatty Acid Metabolism (Acyl Glycine) | 1.57 | 2.4E-02 |
| n,n,n-trimethyl-alanylproline betaine (tmap) | Urea cycle; Arginine and Proline Metabolism | 1.57 | <3.4E-04 |
| vanillactate | Tyrosine Metabolism | 1.57 | 4.0E-04 |
| decadienedioic acid (c10:2-dc) | Fatty Acid, Dicarboxylate | 1.57 | 3.0E-02 |
| succinoyltaurine | Methionine, Cysteine, SAM and Taurine Metabolism | 1.56 | 6.7E-03 |
| 3-hydroxyhexanoylcarnitine (1) | Fatty Acid Metabolism (Acyl Carnitine, Hydroxy) | 1.55 | 1.9E-03 |
| n-acetylglucosaminylasparagine | Aminosugar Metabolism | 1.55 | 8.9E-03 |
| 2-methylbutyrylcarnitine (c5) | Leucine, Isoleucine and Valine Metabolism | 1.55 | 1.7E-03 |
| kynurenate | Tryptophan Metabolism | 1.55 | <3.4E-04 |
| 3-carboxy-4-methyl-5-pentyl-2-furanpropionate (3-cmpfp) | Fatty Acid, Dicarboxylate | 1.55 | 1.6E-03 |
| 4-acetamidobutanoate | Polyamine Metabolism | 1.54 | <3.4E-04 |
| 5-(galactosylhydroxy)-l-lysine | Lysine Metabolism | 1.54 | 1.0E-02 |
| n-acetyl-2-aminooctanoate | Fatty Acid, Amino | 1.54 | 3.5E-03 |
| 2,3-dihydroxy-2-methylbutyrate | Leucine, Isoleucine and Valine Metabolism | 1.54 | 4.8E-03 |
| gamma-cehc glucuronide | Tocopherol Metabolism | 1.54 | 8.7E-03 |
| quinolinate | Nicotinate and Nicotinamide Metabolism | 1.53 | 1.3E-03 |
| methionine sulfone | Methionine, Cysteine, SAM and Taurine Metabolism | 1.52 | 1.2E-03 |
| adipoylcarnitine (c6-dc) | Fatty Acid Metabolism (Acyl Carnitine, Dicarboxylate) | 1.52 | 6.5E-03 |
| argininate | Urea cycle; Arginine and Proline Metabolism | 1.52 | 8.1E-03 |
| dopamine 4-sulfate | Tyrosine Metabolism | 1.52 | 5.9E-03 |
| mannitol/sorbitol | Fructose, Mannose and Galactose Metabolism | 1.51 | 1.6E-02 |
| 4-hydroxyphenylacetoylcarnitine | Tyrosine Metabolism | 1.51 | 2.5E-02 |
| lyxonate | Pentose Metabolism | 1.51 | 1.7E-03 |
| 2r,3r-dihydroxybutyrate | Fatty Acid, Dihydroxy | 1.50 | 5.8E-04 |
| n-acetylhistidine | Histidine Metabolism | 1.49 | 4.5E-04 |
| n-acetylcarnosine | Histidine Metabolism | 1.49 | 3.5E-03 |
| retinol (vitamin a) | Vitamin A Metabolism | 1.48 | <3.4E-04 |
| androsterone glucuronide | Androgenic Steroids | 1.48 | 4.3E-02 |
| isovalerylglycine | Leucine, Isoleucine and Valine Metabolism | 1.48 | 5.0E-03 |
| n-carbamoylalanine | Alanine and Aspartate Metabolism | 1.48 | 2.0E-02 |
| orotidine | Pyrimidine Metabolism, Orotate containing | 1.48 | 4.1E-04 |
| 3-aminoisobutyrate | Pyrimidine Metabolism, Thymine containing | 1.47 | 1.2E-02 |
| 2-methylmalonylcarnitine (c4-dc) | Fatty Acid Metabolism (also BCAA Metabolism) | 1.47 | 1.5E-03 |
| campesterol | Sterol | 1.47 | 4.4E-02 |
| indoleacetate | Tryptophan Metabolism | 1.47 | 7.6E-03 |
| citraconate/glutaconate | TCA Cycle | 1.47 | 5.8E-03 |
| pentose acid | Partially Characterized Molecules | 1.47 | 4.9E-02 |
| 1-stearoyl-gpg (18:0) | Lysophospholipid | 1.46 | 2.0E-02 |
| n1-methylinosine | Purine Metabolism, (Hypo)Xanthine/Inosine containing | 1.46 | <3.4E-04 |
| indolelactate | Tryptophan Metabolism | 1.46 | 4.0E-04 |
| 2,3-dihydroxy-5-methylthio-4-pentenoate (dmtpa) | Methionine, Cysteine, SAM and Taurine Metabolism | 1.46 | <3.4E-04 |
| 5-dodecenoylcarnitine (c12:1) | Fatty Acid Metabolism (Acyl Carnitine, Monounsaturated) | 1.46 | 2.8E-02 |
| 3-indoxyl sulfate | Tryptophan Metabolism | 1.45 | 1.1E-02 |
| erythritol | Food Component/Plant | 1.45 | 1.3E-03 |
| 3-(3-amino-3-carboxypropyl)uridine | Pyrimidine Metabolism, Uracil containing | 1.45 | <3.4E-04 |
| 3-hydroxydecanoylcarnitine | Fatty Acid Metabolism (Acyl Carnitine, Hydroxy) | 1.45 | 1.4E-02 |
| o-sulfo-l-tyrosine | Chemical | 1.45 | <3.4E-04 |
| ceramide (d18:1/17:0, d17:1/18:0) | Ceramides | 1.45 | 1.3E-02 |
| 1-(1-enyl-stearoyl)-2-linoleoyl-gpe (p-18:0/18:2) | Plasmalogen | 1.44 | 1.4E-03 |
| arabitol/xylitol | Pentose Metabolism | 1.44 | <3.4E-04 |
| n-acetylphenylalanine | Phenylalanine Metabolism | 1.44 | 6.6E-04 |
| n-delta-acetylornithine | Urea cycle; Arginine and Proline Metabolism | 1.44 | 3.3E-02 |
| 3-hydroxybutyroylglycine | Fatty Acid Metabolism (Acyl Glycine) | 1.43 | 1.5E-02 |
| 1-stearoyl-2-oleoyl-gpe (18:0/18:1) | Phosphatidylethanolamine (PE) | 1.43 | 1.3E-02 |
| margaroylcarnitine (c17) | Fatty Acid Metabolism (Acyl Carnitine, Long Chain Saturated) | 1.43 | 6.5E-03 |
| alpha-ketoglutaramate | Glutamate Metabolism | 1.43 | 6.4E-04 |
| 5-methylthioribose | Methionine, Cysteine, SAM and Taurine Metabolism | 1.42 | <3.4E-04 |
| hydroxyasparagine | Alanine and Aspartate Metabolism | 1.42 | <3.4E-04 |
| myo-inositol | Inositol Metabolism | 1.42 | 2.1E-03 |
| 3-formylindole | Food Component/Plant | 1.41 | 3.2E-03 |
| arabonate/xylonate | Pentose Metabolism | 1.41 | 3.1E-03 |
| hwesasxx | Polypeptide | 1.41 | 1.8E-02 |
| c-glycosyltryptophan | Tryptophan Metabolism | 1.40 | <3.4E-04 |
| sphingomyelin (d17:2/16:0, d18:2/15:0) | Sphingomyelins | 1.40 | 4.1E-03 |
| n6,n6-dimethyllysine | Lysine Metabolism | 1.40 | 2.6E-03 |
| n6-carbamoylthreonyladenosine | Purine Metabolism, Adenine containing | 1.40 | <3.4E-04 |
| 2-o-methylascorbic acid | Ascorbate and Aldarate Metabolism | 1.40 | 1.4E-03 |
| 1-(1-enyl-stearoyl)-2-oleoyl-gpe (p-18:0/18:1) | Plasmalogen | 1.40 | 2.9E-03 |
| glucuronate | Aminosugar Metabolism | 1.40 | 4.6E-03 |
| n-acetylarginine | Urea cycle; Arginine and Proline Metabolism | 1.40 | 1.6E-03 |
| n,n-dimethylalanine | Alanine and Aspartate Metabolism | 1.40 | 1.9E-02 |
| n-acetylneuraminate | Aminosugar Metabolism | 1.39 | 4.6E-04 |
| s-adenosylhomocysteine (sah) | Methionine, Cysteine, SAM and Taurine Metabolism | 1.39 | 4.9E-04 |
| creatinine | Creatine Metabolism | 1.39 | <3.4E-04 |
| beta-hydroxyisovalerate | Leucine, Isoleucine and Valine Metabolism | 1.39 | 6.1E-03 |
| 5,6-dihydrouridine | Pyrimidine Metabolism, Uracil containing | 1.39 | 3.4E-04 |
| 1-palmitoyl-2-linoleoyl-gpe (16:0/18:2) | Phosphatidylethanolamine (PE) | 1.38 | 1.1E-02 |
| taurocholenate sulfate | Secondary Bile Acid Metabolism | 1.38 | 4.1E-02 |
| n6-succinyladenosine | Purine Metabolism, Adenine containing | 1.38 | 3.8E-04 |
| vanillylmandelate (vma) | Tyrosine Metabolism | 1.38 | 4.9E-04 |
| n-behenoyl-sphingadienine (d18:2/22:0) | Ceramides | 1.37 | 2.8E-03 |
| pseudouridine | Pyrimidine Metabolism, Uracil containing | 1.37 | <3.4E-04 |
| imidazole lactate | Histidine Metabolism | 1.37 | 1.3E-03 |
| sphingomyelin (d18:1/25:0, d19:0/24:1, d20:1/23:0, d19:1/24:0) | Sphingomyelins | 1.36 | 4.9E-02 |
| 3-hydroxy-2-ethylpropionate | Leucine, Isoleucine and Valine Metabolism | 1.36 | 1.0E-02 |
| kynurenine | Tryptophan Metabolism | 1.36 | 3.7E-04 |
| n-formylanthranilic acid | Tryptophan Metabolism | 1.36 | 2.6E-02 |
| 2-stearoyl-gpe (18:0) | Lysophospholipid | 1.36 | 8.4E-03 |
| mannonate | Food Component/Plant | 1.36 | 9.6E-04 |
| 1-(1-enyl-palmitoyl)-2-linoleoyl-gpe (p-16:0/18:2) | Plasmalogen | 1.35 | 4.6E-03 |
| ascorbic acid 3-sulfate | Ascorbate and Aldarate Metabolism | 1.35 | 1.4E-03 |
| sphingomyelin (d17:1/14:0, d16:1/15:0) | Sphingomyelins | 1.35 | 1.7E-02 |
| n-acetylglutamine | Glutamate Metabolism | 1.35 | 2.6E-03 |
| homovanillate (hva) | Tyrosine Metabolism | 1.35 | 5.0E-03 |
| n-palmitoyl-heptadecasphingosine (d17:1/16:0) | Ceramides | 1.35 | 8.3E-03 |
| glutamine_degradant | Partially Characterized Molecules | 1.35 | 3.2E-02 |
| 1-(1-enyl-stearoyl)-2-arachidonoyl-gpe (p-18:0/20:4) | Plasmalogen | 1.34 | 1.2E-02 |
| 2-methylcitrate/homocitrate | TCA Cycle | 1.34 | 5.7E-04 |
| 1-oleoyl-2-linoleoyl-gpe (18:1/18:2) | Phosphatidylethanolamine (PE) | 1.34 | 2.3E-02 |
| n-acetyltryptophan | Tryptophan Metabolism | 1.34 | 2.1E-02 |
| erythronate | Aminosugar Metabolism | 1.33 | <3.4E-04 |
| dopamine 3-o-sulfate | Tyrosine Metabolism | 1.33 | 2.2E-02 |
| decanoylcarnitine (c10) | Fatty Acid Metabolism (Acyl Carnitine, Medium Chain) | 1.33 | 4.4E-02 |
| 1-(1-enyl-stearoyl)-gpe (p-18:0) | Lysoplasmalogen | 1.33 | 5.1E-03 |
| cis-4-decenoylcarnitine (c10:1) | Fatty Acid Metabolism (Acyl Carnitine, Monounsaturated) | 1.33 | 1.7E-02 |
| n-acetylthreonine | Glycine, Serine and Threonine Metabolism | 1.32 | 1.2E-03 |
| formiminoglutamate | Histidine Metabolism | 1.32 | 1.6E-02 |
| n-stearoyl-sphingadienine (d18:2/18:0) | Ceramides | 1.32 | 1.3E-02 |
| 1-oleoyl-gpi (18:1) | Lysophospholipid | 1.31 | 1.6E-02 |
| maleate | Fatty Acid, Dicarboxylate | 1.31 | 4.1E-02 |
| 1-ribosyl-imidazoleacetate | Histidine Metabolism | 1.31 | 4.6E-03 |
| octanoylcarnitine (c8) | Fatty Acid Metabolism (Acyl Carnitine, Medium Chain) | 1.31 | 4.6E-02 |
| laurylcarnitine (c12) | Fatty Acid Metabolism (Acyl Carnitine, Medium Chain) | 1.31 | 3.9E-02 |
| 3-indoleglyoxylic acid | Food Component/Plant | 1.31 | 1.1E-02 |
| gamma-glutamylisoleucine | Gamma-glutamyl Amino Acid | 1.31 | 4.2E-03 |
| 4-guanidinobutanoate | Guanidino and Acetamido Metabolism | 1.30 | 5.0E-02 |
| glycerol 3-phosphate | Glycerolipid Metabolism | 1.30 | 7.2E-03 |
| trans-4-hydroxyproline | Urea cycle; Arginine and Proline Metabolism | 1.30 | 7.0E-03 |
| n-stearoyltaurine | Endocannabinoid | 1.29 | 3.8E-02 |
| choline phosphate | Phospholipid Metabolism | 1.29 | 1.5E-02 |
| ceramide (d18:2/24:1, d18:1/24:2) | Ceramides | 1.29 | 4.7E-03 |
| acetylcarnitine (c2) | Fatty Acid Metabolism (Acyl Carnitine, Short Chain) | 1.28 | 1.5E-02 |
| 1-oleoyl-gpe (18:1) | Lysophospholipid | 1.28 | 1.7E-02 |
| cystine | Methionine, Cysteine, SAM and Taurine Metabolism | 1.28 | 4.7E-04 |
| arachidoylcarnitine (c20) | Fatty Acid Metabolism (Acyl Carnitine, Long Chain Saturated) | 1.27 | 1.7E-02 |
| 5-methylthioadenosine (mta) | Polyamine Metabolism | 1.27 | 1.5E-03 |
| citrulline | Urea cycle; Arginine and Proline Metabolism | 1.27 | 3.8E-04 |
| succinylcarnitine (c4-dc) | TCA Cycle | 1.27 | 2.8E-02 |
| 1-(1-enyl-palmitoyl)-2-arachidonoyl-gpe (p-16:0/20:4) | Plasmalogen | 1.27 | 2.4E-02 |
| n-acetylserine | Glycine, Serine and Threonine Metabolism | 1.27 | 7.1E-04 |
| 2-hydroxyoctanoate | Fatty Acid, Monohydroxy | 1.27 | 2.6E-02 |
| dimethylglycine | Glycine, Serine and Threonine Metabolism | 1.26 | 4.2E-03 |
| n-acetyltyrosine | Tyrosine Metabolism | 1.26 | 6.6E-03 |
| 1-(1-enyl-palmitoyl)-2-linoleoyl-gpc (p-16:0/18:2) | Plasmalogen | 1.26 | 1.6E-03 |
| 1-methyl-4-imidazoleacetate | Histidine Metabolism | 1.26 | 1.6E-02 |
| gluconate | Food Component/Plant | 1.26 | 4.8E-03 |
| 1-stearoyl-2-oleoyl-gpi (18:0/18:1) | Phosphatidylinositol (PI) | 1.26 | 3.6E-02 |
| sphingomyelin (d17:1/16:0, d18:1/15:0, d16:1/17:0) | Sphingomyelins | 1.25 | 4.6E-03 |
| 2-ketocaprylate | Leucine, Isoleucine and Valine Metabolism | 1.25 | 4.7E-02 |
| n2,n2-dimethylguanosine | Purine Metabolism, Guanine containing | 1.25 | 4.3E-04 |
| n6-acetyllysine | Lysine Metabolism | 1.25 | 3.6E-04 |
| n-acetylalanine | Alanine and Aspartate Metabolism | 1.25 | 3.5E-04 |
| sphingomyelin (d18:2/23:0, d18:1/23:1, d17:1/24:1) | Sphingomyelins | 1.25 | 1.5E-03 |
| phenyllactate (pla) | Phenylalanine Metabolism | 1.25 | 7.8E-03 |
| n-acetylputrescine | Polyamine Metabolism | 1.24 | 2.6E-03 |
| n-acetyl-isoputreanine | Polyamine Metabolism | 1.24 | 8.4E-03 |
| dimethylarginine (sdma + adma) | Urea cycle; Arginine and Proline Metabolism | 1.24 | <3.4E-04 |
| ribonate | Pentose Metabolism | 1.24 | 1.9E-02 |
| sphingomyelin (d18:2/14:0, d18:1/14:1) | Sphingomyelins | 1.24 | 2.5E-02 |
| 3,4-dihydroxybutyrate | Fatty Acid, Dihydroxy | 1.24 | 2.0E-03 |
| sphingomyelin (d18:1/21:0, d17:1/22:0, d16:1/23:0) | Sphingomyelins | 1.24 | 9.2E-03 |
| sphingomyelin (d18:2/18:1) | Sphingomyelins | 1.24 | 1.2E-02 |
| sphingomyelin (d18:2/21:0, d16:2/23:0) | Sphingomyelins | 1.24 | 4.9E-03 |
| 1-(1-enyl-palmitoyl)-gpc (p-16:0) | Lysoplasmalogen | 1.23 | 5.7E-03 |
| 1-linoleoyl-gpi (18:2) | Lysophospholipid | 1.23 | 3.2E-02 |
| n-acetylleucine | Leucine, Isoleucine and Valine Metabolism | 1.23 | 1.4E-02 |
| myristoylcarnitine (c14) | Fatty Acid Metabolism (Acyl Carnitine, Long Chain Saturated) | 1.22 | 4.3E-02 |
| 3-methoxytyramine sulfate | Tyrosine Metabolism | 1.22 | 2.4E-02 |
| n-formylmethionine | Methionine, Cysteine, SAM and Taurine Metabolism | 1.22 | 1.5E-03 |
| 1-palmitoyl-gpe (16:0) | Lysophospholipid | 1.22 | 1.5E-02 |
| urate | Purine Metabolism, (Hypo)Xanthine/Inosine containing | 1.22 | 2.4E-03 |
| ceramide (d18:1/20:0, d16:1/22:0, d20:1/18:0) | Ceramides | 1.21 | 4.9E-02 |
| n-acetyl-beta-alanine | Pyrimidine Metabolism, Uracil containing | 1.21 | 1.1E-02 |
| sphingomyelin (d18:2/23:1) | Sphingomyelins | 1.21 | 1.7E-02 |
| methylsuccinate | Leucine, Isoleucine and Valine Metabolism | 1.21 | 2.8E-02 |
| n-acetylvaline | Leucine, Isoleucine and Valine Metabolism | 1.21 | 2.6E-03 |
| stearoylcarnitine (c18) | Fatty Acid Metabolism (Acyl Carnitine, Long Chain Saturated) | 1.20 | 4.0E-02 |
| 1-linoleoyl-gpe (18:2) | Lysophospholipid | 1.20 | 3.5E-02 |
| allantoin | Purine Metabolism, (Hypo)Xanthine/Inosine containing | 1.20 | 2.0E-02 |
| sphingomyelin (d18:1/17:0, d17:1/18:0, d19:1/16:0) | Sphingomyelins | 1.20 | 6.8E-03 |
| gamma-glutamylphenylalanine | Gamma-glutamyl Amino Acid | 1.20 | 4.0E-03 |
| 2-palmitoyl-gpc (16:0) | Lysophospholipid | 1.19 | 4.1E-02 |
| sulfate | Chemical | 1.19 | 1.4E-03 |
| (n(1) + n(8))-acetylspermidine | Polyamine Metabolism | 1.19 | 2.5E-02 |
| n1-methyladenosine | Purine Metabolism, Adenine containing | 1.19 | 1.4E-03 |
| cholesterol | Sterol | 1.19 | 6.9E-03 |
| n-acetylisoleucine | Leucine, Isoleucine and Valine Metabolism | 1.19 | 3.3E-02 |
| 1-linoleoyl-gpa (18:2) | Lysophospholipid | 1.19 | 3.3E-02 |
| n-acetylasparagine | Alanine and Aspartate Metabolism | 1.19 | 3.6E-02 |
| sphingomyelin (d18:1/14:0, d16:1/16:0) | Sphingomyelins | 1.19 | 1.6E-02 |
| sphingomyelin (d18:1/19:0, d19:1/18:0) | Sphingomyelins | 1.18 | 4.1E-02 |
| 3-hydroxy-3-methylglutarate | Mevalonate Metabolism | 1.18 | 2.0E-02 |
| 1-(1-enyl-palmitoyl)-2-arachidonoyl-gpc (p-16:0/20:4) | Plasmalogen | 1.18 | 3.8E-02 |
| sphingomyelin (d18:2/16:0, d18:1/16:1) | Sphingomyelins | 1.18 | 1.3E-03 |
| n-palmitoyl-sphingosine (d18:1/16:0) | Ceramides | 1.18 | 1.6E-02 |
| tricosanoyl sphingomyelin (d18:1/23:0) | Sphingomyelins | 1.18 | 1.5E-02 |
| 1-(1-enyl-palmitoyl)-2-oleoyl-gpc (p-16:0/18:1) | Plasmalogen | 1.16 | 1.1E-02 |
| lignoceroyl sphingomyelin (d18:1/24:0) | Sphingomyelins | 1.16 | 2.6E-02 |
| 1-stearoyl-gpe (18:0) | Lysophospholipid | 1.16 | 3.4E-02 |
| hydroxypalmitoyl sphingomyelin (d18:1/16:0(oh)) | Sphingomyelins | 1.16 | 1.5E-02 |
| sphingomyelin (d18:1/22:1, d18:2/22:0, d16:1/24:1) | Sphingomyelins | 1.16 | 1.7E-03 |
| 1-(1-enyl-palmitoyl)-2-oleoyl-gpe (p-16:0/18:1) | Plasmalogen | 1.16 | 3.7E-02 |
| gamma-glutamylvaline | Gamma-glutamyl Amino Acid | 1.16 | 1.3E-02 |
| sphingomyelin (d18:1/18:1, d18:2/18:0) | Sphingomyelins | 1.14 | 1.3E-02 |
| glycerophosphoethanolamine | Phospholipid Metabolism | 1.14 | 1.8E-02 |
| 1-linoleoyl-gpc (18:2) | Lysophospholipid | 1.13 | 2.2E-02 |
| methionine sulfoxide | Methionine, Cysteine, SAM and Taurine Metabolism | 1.13 | 1.8E-02 |
| 7-methylguanine | Purine Metabolism, Guanine containing | 1.13 | 5.7E-03 |
| behenoyl sphingomyelin (d18:1/22:0) | Sphingomyelins | 1.12 | 2.4E-02 |
| palmitoyl-sphingosine-phosphoethanolamine (d18:1/16:0) | Ceramide PEs | 1.12 | 1.8E-02 |
| choline | Phospholipid Metabolism | 1.11 | 1.1E-02 |
| sphingomyelin (d18:1/20:1, d18:2/20:0) | Sphingomyelins | 1.10 | 3.7E-02 |
| palmitoyl sphingomyelin (d18:1/16:0) | Sphingomyelins | 1.10 | 2.4E-03 |
| sphingomyelin (d18:1/24:1, d18:2/24:0) | Sphingomyelins | 1.09 | 3.5E-02 |
| n-acetylglucosamine/n-acetylgalactosamine | Aminosugar Metabolism | 1.08 | 4.1E-02 |
| cysteine sulfinic acid | Methionine, Cysteine, SAM and Taurine Metabolism | 1.08 | 4.7E-02 |
| sphingomyelin (d18:2/24:1, d18:1/24:2) | Sphingomyelins | 1.08 | 4.7E-02 |
| 1-palmitoyl-2-linoleoyl-gpc (16:0/18:2) | Phosphatidylcholine (PC) | 1.07 | 1.6E-02 |
| ^1^Fold change represents ratio of geometric means of 90th relative to 10th percentiles of creatinine | |  |  |
| ^2^Adjustment for race, BMI, sex, age, batch, study, batch*study interaction | |  |  |

| **Supplementary Table 8**. Metabolites associated with vegan relative to non-vegetarian dietary patterns at FDR < 0.05 in n=139 AHS-2 participants^1^ | | | |
| --- | --- | --- | --- |
| **Metabolite** | **Subclass** | **Fold Change** | **FDR** |
| s-methylmethionine | Methionine, Cysteine, SAM and Taurine Metabolism | 7.86 | <9.0E-05 |
| 4-ethylphenylsulfate | Benzoate Metabolism | 7.34 | 5.9E-03 |
| 2-acetamidophenol sulfate | Food Component/Plant | 5.58 | 4.9E-04 |
| branched chain 14:0 dicarboxylic acid | Fatty Acid, Dicarboxylate | 4.99 | <9.0E-05 |
| indolepropionate | Tryptophan Metabolism | 4.95 | <9.0E-05 |
| genistein sulfate | Food Component/Plant | 4.60 | 1.9E-02 |
| 2-aminophenol sulfate | Food Component/Plant | 4.49 | <9.0E-05 |
| methyl glucopyranoside (alpha + beta) | Food Component/Plant | 4.49 | <9.0E-05 |
| daidzein sulfate (2) | Food Component/Plant | 4.48 | 2.2E-02 |
| stachydrine | Food Component/Plant | 4.26 | 2.7E-03 |
| 4-acetylphenol sulfate | Benzoate Metabolism | 4.16 | 7.2E-04 |
| 12,13-dihome | Fatty Acid, Dihydroxy | 3.81 | <9.0E-05 |
| gentisate | Tyrosine Metabolism | 3.81 | <9.0E-05 |
| ethyl beta-glucopyranoside | Food Component/Plant | 3.64 | 1.1E-03 |
| beta-cryptoxanthin | Vitamin A Metabolism | 3.55 | <9.0E-05 |
| octadecadienedioate (c18:2-dc) | Fatty Acid, Dicarboxylate | 3.42 | <9.0E-05 |
| 2-butenoylglycine | Fatty Acid Metabolism (Acyl Glycine) | 3.41 | 6.9E-04 |
| linoleoyl-linolenoyl-glycerol (18:2/18:3) [2] | Diacylglycerol | 3.22 | 5.9E-03 |
| 9,10-dihome | Fatty Acid, Dihydroxy | 3.19 | <9.0E-05 |
| 1-linoleoyl-2-linolenoyl-gpc (18:2/18:3) | Phosphatidylcholine (PC) | 3.18 | <9.0E-05 |
| 4-methoxyphenol sulfate | Tyrosine Metabolism | 3.13 | 4.8E-02 |
| 4-allylphenol sulfate | Food Component/Plant | 3.03 | 9.2E-04 |
| n-methylproline | Urea cycle; Arginine and Proline Metabolism | 2.90 | 3.7E-02 |
| glycochenodeoxycholate 3-sulfate | Primary Bile Acid Metabolism | 2.75 | 2.4E-02 |
| glutamine conjugate of c6h10o2 (2) | Partially Characterized Molecules | 2.75 | 1.4E-02 |
| 2,6-dihydroxybenzoic acid | Drug - Topical Agents | 2.74 | <9.0E-05 |
| pentose acid | Partially Characterized Molecules | 2.74 | 1.2E-03 |
| s-methylcysteine sulfoxide | Methionine, Cysteine, SAM and Taurine Metabolism | 2.57 | <9.0E-05 |
| 1,2-dilinoleoyl-gpe (18:2/18:2) | Phosphatidylethanolamine (PE) | 2.54 | 7.4E-04 |
| n-linoleoylglycine | Fatty Acid Metabolism (Acyl Glycine) | 2.52 | <9.0E-05 |
| linolenate [alpha or gamma; (18:3n3 or 6)] | Long Chain Polyunsaturated Fatty Acid (n3 and n6) | 2.50 | 4.1E-04 |
| n-delta-acetylornithine | Urea cycle; Arginine and Proline Metabolism | 2.50 | <9.0E-05 |
| 1-palmitoleoyl-2-linolenoyl-gpc (16:1/18:3) | Phosphatidylcholine (PC) | 2.42 | 4.0E-04 |
| 4-acetylcatechol sulfate (1) | Food Component/Plant | 2.41 | 1.4E-02 |
| tryptophan betaine | Tryptophan Metabolism | 2.40 | 3.8E-02 |
| 4-allylcatechol sulfate | Benzoate Metabolism | 2.37 | 4.5E-02 |
| stearidonate (18:4n3) | Long Chain Polyunsaturated Fatty Acid (n3 and n6) | 2.35 | 6.7E-04 |
| 1-lignoceroyl-gpc (24:0) | Lysophospholipid | 2.33 | <9.0E-05 |
| trans-2-hexenoylglycine | Fatty Acid Metabolism (Acyl Glycine) | 2.31 | 1.7E-03 |
| docosatrienoate (22:3n3) | Long Chain Polyunsaturated Fatty Acid (n3 and n6) | 2.24 | 1.1E-02 |
| carboxyethyl-gaba | Glutamate Metabolism | 2.20 | 6.8E-03 |
| 3-hydroxybutyroylglycine | Fatty Acid Metabolism (Acyl Glycine) | 2.11 | <9.0E-05 |
| isocitrate | TCA Cycle | 2.09 | <9.0E-05 |
| 13-hode + 9-hode | Fatty Acid, Monohydroxy | 2.09 | 4.3E-04 |
| histidine betaine (hercynine) | Food Component/Plant | 2.06 | 1.1E-02 |
| catechol sulfate | Benzoate Metabolism | 2.05 | 1.4E-02 |
| cis-4-decenoate (10:1n6) | Medium Chain Fatty Acid | 2.03 | <9.0E-05 |
| octadecenedioate (c18:1-dc) | Fatty Acid, Dicarboxylate | 2.01 | <9.0E-05 |
| tetradecadienoate (14:2) | Long Chain Polyunsaturated Fatty Acid (n3 and n6) | 1.98 | 1.0E-03 |
| guaiacol sulfate | Benzoate Metabolism | 1.95 | 1.4E-02 |
| ergothioneine | Food Component/Plant | 1.95 | 8.9E-04 |
| pyridoxate | Vitamin B6 Metabolism | 1.93 | 3.7E-02 |
| s-methylcysteine | Methionine, Cysteine, SAM and Taurine Metabolism | 1.92 | 1.1E-02 |
| 2,3-dihydroxy-2-methylbutyrate | Leucine, Isoleucine and Valine Metabolism | 1.90 | 3.3E-03 |
| hypotaurine | Methionine, Cysteine, SAM and Taurine Metabolism | 1.86 | 9.9E-04 |
| linoleoyl-arachidonoyl-glycerol (18:2/20:4) [1] | Diacylglycerol | 1.84 | 5.7E-03 |
| 1-stearoyl-2-oleoyl-gps (18:0/18:1) | Phosphatidylserine (PS) | 1.83 | 6.7E-03 |
| carotene diol (1) | Vitamin A Metabolism | 1.83 | 1.7E-03 |
| dodecadienoate (12:2) | Fatty Acid, Dicarboxylate | 1.83 | 4.7E-04 |
| carotene diol (2) | Vitamin A Metabolism | 1.81 | 8.1E-04 |
| linolenoylcarnitine (c18:3) | Fatty Acid Metabolism (Acyl Carnitine, Polyunsaturated) | 1.77 | 2.1E-03 |
| hexadecadienoate (16:2n6) | Long Chain Polyunsaturated Fatty Acid (n3 and n6) | 1.76 | 1.3E-02 |
| (2 or 3)-decenoate (10:1n7 or n8) | Medium Chain Fatty Acid | 1.75 | 4.4E-03 |
| 1-oleoyl-2-arachidonoyl-gpe (18:1/20:4) | Phosphatidylethanolamine (PE) | 1.73 | 1.4E-02 |
| n-linoleoyltaurine | Endocannabinoid | 1.72 | 1.6E-02 |
| n2,n5-diacetylornithine | Urea cycle; Arginine and Proline Metabolism | 1.69 | 4.7E-02 |
| 1,2-dilinoleoyl-gpc (18:2/18:2) | Phosphatidylcholine (PC) | 1.68 | 4.5E-04 |
| dodecanedioate (c12-dc) | Fatty Acid, Dicarboxylate | 1.61 | 4.8E-02 |
| 1-oleoyl-2-linoleoyl-gpe (18:1/18:2) | Phosphatidylethanolamine (PE) | 1.58 | 3.8E-02 |
| oxalate (ethanedioate) | Ascorbate and Aldarate Metabolism | 1.57 | <9.0E-05 |
| 2-aminoheptanoate | Fatty Acid, Amino | 1.55 | 1.9E-02 |
| sphingomyelin (d18:2/24:2) | Sphingomyelins | 1.53 | 7.9E-04 |
| oleoyl-linoleoyl-glycerol (18:1/18:2) [1] | Diacylglycerol | 1.53 | 3.3E-02 |
| bilirubin (e,e) | Hemoglobin and Porphyrin Metabolism | 1.50 | 3.3E-02 |
| 1-ribosyl-imidazoleacetate | Histidine Metabolism | 1.49 | 2.0E-03 |
| gamma-glutamylglycine | Gamma-glutamyl Amino Acid | 1.49 | 5.7E-04 |
| 1-stearoyl-2-linoleoyl-gpe (18:0/18:2) | Phosphatidylethanolamine (PE) | 1.47 | 3.7E-02 |
| 1-stearoyl-2-linoleoyl-gpi (18:0/18:2) | Phosphatidylinositol (PI) | 1.46 | 3.7E-03 |
| 1-palmitoyl-2-linoleoyl-gpi (16:0/18:2) | Phosphatidylinositol (PI) | 1.44 | 4.8E-02 |
| lactosyl-n-nervonoyl-sphingosine (d18:1/24:1) | Lactosylceramides (LCER) | 1.43 | 1.3E-02 |
| 7-alpha-hydroxy-3-oxo-4-cholestenoate (7-hoca) | Sterol | 1.42 | 4.1E-03 |
| glycine | Glycine, Serine and Threonine Metabolism | 1.42 | 1.7E-03 |
| 1-linoleoyl-gpe (18:2) | Lysophospholipid | 1.38 | 2.5E-02 |
| glycosyl ceramide (d18:2/24:1, d18:1/24:2) | Hexosylceramides (HCER) | 1.36 | 1.5E-02 |
| 1-linoleoyl-2-arachidonoyl-gpc (18:2/20:4n6) | Phosphatidylcholine (PC) | 1.35 | 2.1E-02 |
| gamma-glutamylglutamine | Gamma-glutamyl Amino Acid | 1.34 | 1.1E-02 |
| isoleucylglycine | Dipeptide | 1.34 | 2.4E-02 |
| n-acetyl-isoputreanine | Polyamine Metabolism | 1.34 | 1.9E-02 |
| sphingomyelin (d18:2/24:1, d18:1/24:2) | Sphingomyelins | 1.32 | 1.6E-02 |
| palmitoyl-sphingosine-phosphoethanolamine (d18:1/16:0) | Ceramide PEs | 1.31 | 3.6E-02 |
| palmitoyl dihydrosphingomyelin (d18:0/16:0) | Dihydrosphingomyelins | 1.28 | 4.1E-02 |
| citrate | TCA Cycle | 1.23 | 2.4E-02 |
| betaine | Glycine, Serine and Threonine Metabolism | 1.23 | 3.7E-02 |
| lactosyl-n-palmitoyl-sphingosine (d18:1/16:0) | Lactosylceramides (LCER) | 1.23 | 3.8E-02 |
| alpha-tocopherol | Tocopherol Metabolism | 1.21 | 3.8E-02 |
| 1-stearoyl-2-arachidonoyl-gpi (18:0/20:4) | Phosphatidylinositol (PI) | 1.21 | 4.6E-02 |
| citrulline | Urea cycle; Arginine and Proline Metabolism | 1.20 | 1.5E-02 |
| cystine | Methionine, Cysteine, SAM and Taurine Metabolism | 0.85 | 4.9E-02 |
| urate | Purine Metabolism, (Hypo)Xanthine/Inosine containing | 0.85 | 4.6E-02 |
| 2-methylcitrate/homocitrate | TCA Cycle | 0.82 | 4.7E-02 |
| gamma-glutamylvaline | Gamma-glutamyl Amino Acid | 0.82 | 3.4E-02 |
| threonine | Glycine, Serine and Threonine Metabolism | 0.81 | 7.7E-03 |
| gamma-glutamyl-alpha-lysine | Gamma-glutamyl Amino Acid | 0.81 | 2.6E-02 |
| 5-hydroxylysine | Lysine Metabolism | 0.80 | 2.0E-02 |
| kynurenine | Tryptophan Metabolism | 0.79 | 2.6E-02 |
| stearoyl sphingomyelin (d18:1/18:0) | Sphingomyelins | 0.79 | 4.8E-02 |
| lysine | Lysine Metabolism | 0.79 | 2.0E-03 |
| n-palmitoyl-sphingosine (d18:1/16:0) | Ceramides | 0.79 | 4.0E-02 |
| valine | Leucine, Isoleucine and Valine Metabolism | 0.79 | 2.3E-02 |
| n-acetyl-beta-alanine | Pyrimidine Metabolism, Uracil containing | 0.77 | 8.7E-03 |
| sphingomyelin (d18:2/18:1) | Sphingomyelins | 0.76 | 4.9E-02 |
| 2-hydroxy-3-methylvalerate | Leucine, Isoleucine and Valine Metabolism | 0.74 | 3.3E-02 |
| 1-(1-enyl-palmitoyl)-gpc (p-16:0) | Lysoplasmalogen | 0.74 | 6.0E-03 |
| n-acetyltyrosine | Tyrosine Metabolism | 0.74 | 2.0E-02 |
| sarcosine | Glycine, Serine and Threonine Metabolism | 0.72 | 1.9E-02 |
| urea | Urea cycle; Arginine and Proline Metabolism | 0.72 | 1.5E-02 |
| gamma-glutamylthreonine | Gamma-glutamyl Amino Acid | 0.72 | 8.2E-03 |
| quinolinate | Nicotinate and Nicotinamide Metabolism | 0.71 | 2.9E-02 |
| sphingomyelin (d18:0/20:0, d16:0/22:0) | Dihydrosphingomyelins | 0.70 | 3.9E-02 |
| sphingomyelin (d18:2/23:1) | Sphingomyelins | 0.69 | 6.6E-03 |
| palmitoylcarnitine (c16) | Fatty Acid Metabolism (Acyl Carnitine, Long Chain Saturated) | 0.69 | 1.3E-02 |
| 1-(1-enyl-palmitoyl)-gpe (p-16:0) | Lysoplasmalogen | 0.69 | 1.3E-03 |
| 1-palmitoyl-gpg (16:0) | Lysophospholipid | 0.69 | 1.8E-02 |
| 2-oleoylglycerol (18:1) | Monoacylglycerol | 0.68 | 3.8E-02 |
| homocitrulline | Urea cycle; Arginine and Proline Metabolism | 0.68 | 4.0E-02 |
| 1-myristoyl-2-palmitoyl-gpc (14:0/16:0) | Phosphatidylcholine (PC) | 0.68 | 5.0E-02 |
| 3,4-dihydroxybutyrate | Fatty Acid, Dihydroxy | 0.68 | 1.5E-02 |
| adipoylcarnitine (c6-dc) | Fatty Acid Metabolism (Acyl Carnitine, Dicarboxylate) | 0.67 | 3.4E-02 |
| 2-aminobutyrate | Glutathione Metabolism | 0.67 | 1.6E-04 |
| stearoylcarnitine (c18) | Fatty Acid Metabolism (Acyl Carnitine, Long Chain Saturated) | 0.67 | 1.6E-03 |
| n,n-dimethylalanine | Alanine and Aspartate Metabolism | 0.66 | 2.6E-02 |
| 2s,3r-dihydroxybutyrate | Fatty Acid, Dihydroxy | 0.66 | 2.9E-02 |
| pentadecanoate (15:0) | Long Chain Saturated Fatty Acid | 0.65 | 6.6E-03 |
| butyrylcarnitine (c4) | Fatty Acid Metabolism (also BCAA Metabolism) | 0.65 | 1.4E-02 |
| sphingomyelin (d18:1/14:0, d16:1/16:0) | Sphingomyelins | 0.65 | 3.7E-04 |
| isovalerylglycine | Leucine, Isoleucine and Valine Metabolism | 0.65 | 4.0E-02 |
| phenylpyruvate | Phenylalanine Metabolism | 0.64 | 2.0E-02 |
| creatine | Creatine Metabolism | 0.64 | 1.7E-03 |
| ceramide (d18:1/20:0, d16:1/22:0, d20:1/18:0) | Ceramides | 0.64 | 1.2E-03 |
| xanthurenate | Tryptophan Metabolism | 0.63 | 2.5E-02 |
| 1-palmitoyl-gpi (16:0) | Lysophospholipid | 0.63 | 3.3E-02 |
| 1-palmitoylglycerol (16:0) | Monoacylglycerol | 0.63 | 4.6E-02 |
| mannose | Fructose, Mannose and Galactose Metabolism | 0.62 | 2.1E-02 |
| carnitine | Carnitine Metabolism | 0.62 | 1.4E-03 |
| n-acetylcitrulline | Urea cycle; Arginine and Proline Metabolism | 0.62 | 3.8E-02 |
| glutarylcarnitine (c5-dc) | Lysine Metabolism | 0.62 | 1.0E-02 |
| sphingomyelin (d18:1/21:0, d17:1/22:0, d16:1/23:0) | Sphingomyelins | 0.62 | 9.3E-05 |
| perfluorooctanoate (pfoa) | Chemical | 0.61 | 1.1E-02 |
| myristoylcarnitine (c14) | Fatty Acid Metabolism (Acyl Carnitine, Long Chain Saturated) | 0.61 | 1.1E-03 |
| 5alpha-androstan-3alpha,17alpha-diol monosulfate | Androgenic Steroids | 0.60 | 3.9E-02 |
| sphingomyelin (d18:1/17:0, d17:1/18:0, d19:1/16:0) | Sphingomyelins | 0.60 | 1.3E-04 |
| 10-undecenoate (11:1n1) | Medium Chain Fatty Acid | 0.60 | 1.9E-03 |
| cortolone glucuronide (1) | Corticosteroids | 0.60 | 1.5E-02 |
| 2-hydroxy-4-(methylthio)butanoic acid | Methionine, Cysteine, SAM and Taurine Metabolism | 0.60 | 1.1E-02 |
| docosahexaenoate (dha; 22:6n3) | Long Chain Polyunsaturated Fatty Acid (n3 and n6) | 0.60 | 1.1E-02 |
| 1-(1-enyl-palmitoyl)-2-arachidonoyl-gpc (p-16:0/20:4) | Plasmalogen | 0.59 | 8.8E-05 |
| 4-hydroxyphenylpyruvate | Tyrosine Metabolism | 0.59 | 1.8E-02 |
| guanosine | Purine Metabolism, Guanine containing | 0.59 | 4.0E-02 |
| anthranilate | Tryptophan Metabolism | 0.59 | 7.5E-03 |
| decadienedioic acid (c10:2-dc) | Fatty Acid, Dicarboxylate | 0.58 | 3.1E-02 |
| n-stearoyltaurine | Endocannabinoid | 0.58 | 1.0E-03 |
| sphingomyelin (d18:1/19:0, d19:1/18:0) | Sphingomyelins | 0.58 | 1.1E-04 |
| n-formylanthranilic acid | Tryptophan Metabolism | 0.58 | 2.6E-03 |
| gamma-tocopherol/beta-tocopherol | Tocopherol Metabolism | 0.58 | 7.4E-03 |
| sphingomyelin (d17:1/16:0, d18:1/15:0, d16:1/17:0) | Sphingomyelins | 0.57 | 1.5E-04 |
| margarate (17:0) | Long Chain Saturated Fatty Acid | 0.57 | 2.6E-03 |
| isovalerylcarnitine (c5) | Leucine, Isoleucine and Valine Metabolism | 0.57 | 2.6E-03 |
| 1-methylhistidine | Histidine Metabolism | 0.57 | 2.3E-04 |
| indoleacetate | Tryptophan Metabolism | 0.57 | 2.2E-03 |
| 1-(1-enyl-stearoyl)-2-linoleoyl-gpe (p-18:0/18:2) | Plasmalogen | 0.56 | 1.2E-04 |
| 10-nonadecenoate (19:1n9) | Long Chain Monounsaturated Fatty Acid | 0.56 | 7.1E-03 |
| formiminoglutamate | Histidine Metabolism | 0.56 | 1.7E-04 |
| 2-hydroxybutyrate/2-hydroxyisobutyrate | Glutathione Metabolism | 0.56 | 2.1E-02 |
| sphingomyelin (d18:0/18:0, d19:0/17:0) | Dihydrosphingomyelins | 0.55 | 1.0E-03 |
| taurocholenate sulfate | Secondary Bile Acid Metabolism | 0.55 | 6.8E-03 |
| beta-hydroxyisovalerate | Leucine, Isoleucine and Valine Metabolism | 0.53 | 2.3E-04 |
| 6-oxopiperidine-2-carboxylate | Lysine Metabolism | 0.53 | 1.6E-04 |
| picolinoylglycine | Fatty Acid Metabolism (Acyl Glycine) | 0.53 | 7.1E-04 |
| 1-(1-enyl-palmitoyl)-2-linoleoyl-gpe (p-16:0/18:2) | Plasmalogen | 0.53 | 1.1E-04 |
| beta-hydroxyisovaleroylcarnitine | Leucine, Isoleucine and Valine Metabolism | 0.53 | 1.5E-04 |
| trimethylamine n-oxide | Phospholipid Metabolism | 0.53 | 6.4E-03 |
| 3-hydroxy-2-ethylpropionate | Leucine, Isoleucine and Valine Metabolism | 0.52 | 8.9E-05 |
| hydantoin-5-propionate | Histidine Metabolism | 0.52 | 1.9E-02 |
| 3-indoxyl sulfate | Tryptophan Metabolism | 0.52 | 1.4E-03 |
| 3-carboxy-4-methyl-5-pentyl-2-furanpropionate (3-cmpfp) | Fatty Acid, Dicarboxylate | 0.51 | 9.1E-05 |
| glycochenodeoxycholate glucuronide (1) | Primary Bile Acid Metabolism | 0.51 | 4.9E-02 |
| 2r,3r-dihydroxybutyrate | Fatty Acid, Dihydroxy | 0.51 | 9.4E-05 |
| sphingomyelin (d17:2/16:0, d18:2/15:0) | Sphingomyelins | 0.51 | 9.9E-05 |
| 1-arachidonylglycerol (20:4) | Monoacylglycerol | 0.51 | 4.9E-02 |
| 5-hydroxy-2-methylpyridine sulfate | Chemical | 0.50 | 4.8E-02 |
| androsterone glucuronide | Androgenic Steroids | 0.50 | 1.8E-02 |
| phenylacetylglutamate | Acetylated Peptides | 0.50 | 2.9E-02 |
| n-stearoyl-sphingadienine (d18:2/18:0) | Ceramides | 0.50 | 1.1E-04 |
| 1-stearoyl-gpg (18:0) | Lysophospholipid | 0.50 | 9.5E-04 |
| pregnanediol-3-glucuronide | Progestin Steroids | 0.49 | 2.0E-02 |
| 10-heptadecenoate (17:1n7) | Long Chain Monounsaturated Fatty Acid | 0.49 | 3.1E-03 |
| 1-myristoylglycerol (14:0) | Monoacylglycerol | 0.49 | 3.6E-03 |
| taurodeoxycholic acid 3-sulfate | Secondary Bile Acid Metabolism | 0.49 | 4.3E-02 |
| 1-dihomo-linolenylglycerol (20:3) | Monoacylglycerol | 0.49 | 3.9E-02 |
| 6-hydroxyindole sulfate | Chemical | 0.49 | 1.8E-03 |
| (2,4 or 2,5)-dimethylphenol sulfate | Food Component/Plant | 0.48 | 2.9E-02 |
| isobutyrylcarnitine (c4) | Leucine, Isoleucine and Valine Metabolism | 0.48 | 2.3E-04 |
| fructose | Fructose, Mannose and Galactose Metabolism | 0.48 | 7.4E-03 |
| tiglylcarnitine (c5:1-dc) | Leucine, Isoleucine and Valine Metabolism | 0.47 | 1.2E-04 |
| nonanoylcarnitine (c9) | Fatty Acid Metabolism (Acyl Carnitine, Medium Chain) | 0.47 | 1.7E-04 |
| indolin-2-one | Food Component/Plant | 0.47 | 2.9E-03 |
| sphingomyelin (d17:1/14:0, d16:1/15:0) | Sphingomyelins | 0.47 | 1.0E-04 |
| 1-(1-enyl-stearoyl)-2-oleoyl-gpe (p-18:0/18:1) | Plasmalogen | 0.47 | 2.8E-04 |
| phenylacetylglutamine | Acetylated Peptides | 0.47 | 1.4E-03 |
| 1-(1-enyl-stearoyl)-gpe (p-18:0) | Lysoplasmalogen | 0.46 | 3.0E-04 |
| indole-3-carboxylate | Tryptophan Metabolism | 0.46 | 1.6E-04 |
| ceramide (d16:1/24:1, d18:1/22:1) | Ceramides | 0.45 | 1.4E-03 |
| trans-4-hydroxyproline | Urea cycle; Arginine and Proline Metabolism | 0.45 | 6.7E-04 |
| 1-docosahexaenoylglycerol (22:6) | Monoacylglycerol | 0.44 | 3.5E-03 |
| isoursodeoxycholate | Secondary Bile Acid Metabolism | 0.44 | 3.2E-02 |
| taurochenodeoxycholate | Primary Bile Acid Metabolism | 0.44 | 2.0E-02 |
| 3-methyl catechol sulfate (1) | Benzoate Metabolism | 0.44 | 2.9E-02 |
| 2-methylbutyrylcarnitine (c5) | Leucine, Isoleucine and Valine Metabolism | 0.44 | 1.6E-04 |
| 1-pentadecanoylglycerol (15:0) | Monoacylglycerol | 0.44 | 7.5E-03 |
| methyl indole-3-acetate | Food Component/Plant | 0.43 | 1.7E-04 |
| glycoursodeoxycholic acid sulfate (1) | Secondary Bile Acid Metabolism | 0.43 | 4.8E-02 |
| 4-methylguaiacol sulfate | Benzoate Metabolism | 0.43 | 2.1E-02 |
| n-stearoyl-sphingosine (d18:1/18:0) | Ceramides | 0.42 | 1.2E-04 |
| docosahexaenoylcarnitine (c22:6) | Fatty Acid Metabolism (Acyl Carnitine, Polyunsaturated) | 0.42 | 8.9E-04 |
| 1-margaroylglycerol (17:0) | Monoacylglycerol | 0.42 | 1.9E-02 |
| homostachydrine | Food Component/Plant | 0.42 | 7.0E-03 |
| 1-(1-enyl-palmitoyl)-2-arachidonoyl-gpe (p-16:0/20:4) | Plasmalogen | 0.42 | 3.6E-04 |
| propionylcarnitine (c3) | Fatty Acid Metabolism (also BCAA Metabolism) | 0.41 | 1.4E-04 |
| 1-methylurate | Xanthine Metabolism | 0.40 | 2.0E-02 |
| docosapentaenoate (n6 dpa; 22:5n6) | Long Chain Polyunsaturated Fatty Acid (n3 and n6) | 0.40 | 1.1E-04 |
| 1-palmitoleoylglycerol (16:1) | Monoacylglycerol | 0.39 | 2.2E-03 |
| n-palmitoyl-heptadecasphingosine (d17:1/16:0) | Ceramides | 0.39 | 4.1E-04 |
| 2-palmitoleoylglycerol (16:1) | Monoacylglycerol | 0.38 | 2.3E-03 |
| undecenoylcarnitine (c11:1) | Fatty Acid Metabolism (Acyl Carnitine, Monounsaturated) | 0.38 | 9.6E-05 |
| p-cresol sulfate | Benzoate Metabolism | 0.38 | 4.4E-04 |
| 1-(1-enyl-stearoyl)-2-arachidonoyl-gpe (p-18:0/20:4) | Plasmalogen | 0.37 | 6.0E-04 |
| indoleacetylglutamine | Tryptophan Metabolism | 0.37 | 1.3E-03 |
| 4-hydroxyglutamate | Glutamate Metabolism | 0.35 | 1.7E-04 |
| 3,5-dichloro-2,6-dihydroxybenzoic acid | Chemical | 0.35 | 1.7E-04 |
| n-stearoyl-sphinganine (d18:0/18:0) | Dihydroceramides | 0.35 | 1.3E-04 |
| docosatrienoate (22:3n6) | Long Chain Polyunsaturated Fatty Acid (n3 and n6) | 0.34 | 2.2E-03 |
| glutamine conjugate of c7h12o2 | Partially Characterized Molecules | 0.33 | 6.8E-03 |
| saccharin | Food Component/Plant | 0.32 | 1.0E-02 |
| acesulfame | Food Component/Plant | 0.31 | 4.6E-02 |
| n-acetyl-1-methylhistidine | Histidine Metabolism | 0.31 | 9.9E-04 |
| phenylacetate | Phenylalanine Metabolism | 0.30 | 5.1E-04 |
| tyramine o-sulfate | Tyrosine Metabolism | 0.29 | 9.1E-04 |
| tridecenedioate (c13:1-dc) | Fatty Acid, Dicarboxylate | 0.29 | 1.2E-04 |
| 3-methylxanthine | Xanthine Metabolism | 0.28 | 4.1E-03 |
| quinate | Food Component/Plant | 0.28 | 2.1E-02 |
| ceramide (d18:1/17:0, d17:1/18:0) | Ceramides | 0.28 | 4.9E-04 |
| margaroylcarnitine (c17) | Fatty Acid Metabolism (Acyl Carnitine, Long Chain Saturated) | 0.28 | 1.3E-03 |
| (16 or 17)-methylstearate (a19:0 or i19:0) | Fatty Acid, Branched | 0.27 | 5.4E-04 |
| (12 or 13)-methylmyristate (a15:0 or i15:0) | Fatty Acid, Branched | 0.26 | 1.4E-04 |
| 4-acetamidophenol | Drug - Analgesics, Anesthetics | 0.24 | 2.5E-02 |
| sphingomyelin (d18:1/25:0, d19:0/24:1, d20:1/23:0, d19:1/24:0) | Sphingomyelins | 0.23 | 8.9E-04 |
| heptenedioate (c7:1-dc) | Fatty Acid, Dicarboxylate | 0.23 | 3.2E-04 |
| 3-(methylthio)acetaminophen sulfate | Drug - Analgesics, Anesthetics | 0.22 | 5.0E-03 |
| 1,3,7-trimethylurate | Xanthine Metabolism | 0.21 | 9.8E-05 |
| 7-methylxanthine | Xanthine Metabolism | 0.21 | 6.4E-04 |
| 1,3-dimethylurate | Xanthine Metabolism | 0.21 | 1.0E-04 |
| perfluorooctanesulfonate (pfos) | Chemical | 0.19 | 2.7E-04 |
| 4-acetamidophenylglucuronide | Drug - Analgesics, Anesthetics | 0.19 | 2.0E-02 |
| (14 or 15)-methylpalmitate (a17:0 or i17:0) | Fatty Acid, Branched | 0.18 | 7.7E-04 |
| glucuronide of piperine metabolite c17h21no3 (5) | Food Component/Plant | 0.17 | 1.3E-04 |
| phenylacetylcarnitine | Acetylated Peptides | 0.16 | 2.1E-04 |
| n,n,n-trimethyl-5-aminovalerate | Lysine Metabolism | 0.16 | 1.8E-03 |
| sulfate of piperine metabolite c16h19no3 (2) | Food Component/Plant | 0.15 | 2.1E-04 |
| glucuronide of piperine metabolite c17h21no3 (3) | Food Component/Plant | 0.14 | 1.5E-04 |
| 3-bromo-5-chloro-2,6-dihydroxybenzoic acid | Chemical | 0.14 | 1.1E-03 |
| p-cresol glucuronide | Tyrosine Metabolism | 0.14 | 8.7E-05 |
| sulfate of piperine metabolite c18h21no3 (3) | Food Component/Plant | 0.14 | 1.8E-04 |
| 4-acetaminophen sulfate | Drug - Analgesics, Anesthetics | 0.13 | 1.2E-02 |
| sulfate of piperine metabolite c18h21no3 (1) | Food Component/Plant | 0.12 | 1.9E-04 |
| sulfate of piperine metabolite c16h19no3 (3) | Food Component/Plant | 0.11 | 2.4E-04 |
| glucuronide of piperine metabolite c17h21no3 (4) | Food Component/Plant | 0.11 | 1.9E-04 |
| theobromine | Xanthine Metabolism | 0.10 | 1.4E-03 |
| paraxanthine | Xanthine Metabolism | 0.08 | 2.0E-04 |
| 1-methylxanthine | Xanthine Metabolism | 0.08 | 2.6E-04 |
| 1,7-dimethylurate | Xanthine Metabolism | 0.08 | 1.6E-04 |
| hydroxy-cmpf | Fatty Acid, Dicarboxylate | 0.06 | 3.4E-04 |
| theophylline | Xanthine Metabolism | 0.06 | 2.2E-04 |
| piperine | Food Component/Plant | 0.05 | 2.3E-04 |
| 1-methyl-5-imidazoleacetate | Histidine Metabolism | 0.04 | 2.7E-03 |
| 3-carboxy-4-methyl-5-propyl-2-furanpropanoate (cmpf) | Fatty Acid, Dicarboxylate | 0.04 | 4.5E-04 |
| caffeine | Xanthine Metabolism | 0.03 | 1.1E-04 |
| 5-acetylamino-6-amino-3-methyluracil | Xanthine Metabolism | 0.03 | 3.8E-04 |
| 3-methylhistidine | Histidine Metabolism | 0.02 | 5.4E-03 |

^1^Adjustment for race, BMI, sex, age, batch, study, and batch*study

| **Supplementary Table 9**. Plasma metabolites associated with both plasma creatinine and vegan relative to nonvegetarian dietary pattern at FDR < 0.05^1,2^ | | | | | |
| --- | --- | --- | --- | --- | --- |
| **Metabolite** | **Subclass** | **Creatinine** | | **Vegan vs non-vegetarian** | |
|  |  | **Fold Change** | **FDR** | **Fold Change** | **FDR** |
| piperine | Food Component/Plant | 5.10 | 8.4E-03 | 0.05 | 2.3E-04 |
| 1-methyl-5-imidazoleacetate | Histidine Metabolism | 4.29 | 7.9E-04 | 0.04 | 2.7E-03 |
| glucuronide of piperine metabolite c17h21no3 (4) | Food Component/Plant | 4.02 | 2.1E-03 | 0.11 | 1.9E-04 |
| 3-methylhistidine | Histidine Metabolism | 3.96 | 3.2E-03 | 0.02 | 5.4E-03 |
| acesulfame | Food Component/Plant | 3.87 | 3.9E-02 | 0.31 | 4.6E-02 |
| 3-carboxy-4-methyl-5-propyl-2-furanpropanoate (cmpf) | Fatty Acid, Dicarboxylate | 3.72 | 2.8E-02 | 0.04 | 4.5E-04 |
| glucuronide of piperine metabolite c17h21no3 (3) | Food Component/Plant | 3.48 | 2.2E-03 | 0.14 | 1.5E-04 |
| glucuronide of piperine metabolite c17h21no3 (5) | Food Component/Plant | 3.39 | 2.0E-03 | 0.17 | 1.3E-04 |
| n-acetyl-1-methylhistidine | Histidine Metabolism | 3.35 | 6.0E-04 | 0.31 | 9.9E-04 |
| sulfate of piperine metabolite c16h19no3 (3) | Food Component/Plant | 3.30 | 3.8E-03 | 0.11 | 2.4E-04 |
| quinate | Food Component/Plant | 3.18 | 4.9E-02 | 0.28 | 2.1E-02 |
| sulfate of piperine metabolite c18h21no3 (1) | Food Component/Plant | 3.08 | 8.6E-03 | 0.12 | 1.9E-04 |
| sulfate of piperine metabolite c16h19no3 (2) | Food Component/Plant | 3.04 | 2.7E-03 | 0.15 | 2.1E-04 |
| 3-methyl catechol sulfate (1) | Benzoate Metabolism | 2.87 | 2.6E-03 | 0.44 | 2.9E-02 |
| p-cresol glucuronide | Tyrosine Metabolism | 2.74 | 4.2E-02 | 0.14 | 8.6E-05 |
| sulfate of piperine metabolite c18h21no3 (3) | Food Component/Plant | 2.72 | 9.2E-03 | 0.14 | 1.8E-04 |
| glutamine conjugate of c7h12o2 | Partially Characterized Molecules | 2.71 | 8.9E-03 | 0.33 | 6.8E-03 |
| 4-methylguaiacol sulfate | Benzoate Metabolism | 2.70 | 3.3E-03 | 0.43 | 2.1E-02 |
| (2,4 or 2,5)-dimethylphenol sulfate | Food Component/Plant | 2.42 | 4.2E-03 | 0.48 | 2.9E-02 |
| 5-hydroxy-2-methylpyridine sulfate | Chemical | 2.35 | 6.9E-03 | 0.50 | 4.8E-02 |
| indoleacetylglutamine | Tryptophan Metabolism | 2.33 | 1.5E-03 | 0.37 | 1.3E-03 |
| tyramine o-sulfate | Tyrosine Metabolism | 2.28 | 4.5E-03 | 0.29 | 9.1E-04 |
| homocitrulline | Urea cycle; Arginine and Proline Metabolism | 2.23 | 0.0E+00 | 0.68 | 4.0E-02 |
| heptenedioate (c7:1-dc) | Fatty Acid, Dicarboxylate | 2.22 | 1.3E-03 | 0.23 | 3.2E-04 |
| 4-allylcatechol sulfate | Benzoate Metabolism | 2.19 | 1.8E-02 | 2.37 | 4.5E-02 |
| phenylacetylglutamate | Acetylated Peptides | 2.18 | 4.6E-03 | 0.50 | 2.9E-02 |
| methyl indole-3-acetate | Food Component/Plant | 2.04 | 6.3E-04 | 0.43 | 1.7E-04 |
| tridecenedioate (c13:1-dc) | Fatty Acid, Dicarboxylate | 1.95 | 6.8E-03 | 0.29 | 1.2E-04 |
| hydantoin-5-propionate | Histidine Metabolism | 1.94 | 1.4E-03 | 0.52 | 1.9E-02 |
| n-acetylcitrulline | Urea cycle; Arginine and Proline Metabolism | 1.90 | 2.1E-03 | 0.62 | 3.8E-02 |
| pyridoxate | Vitamin B6 Metabolism | 1.85 | 8.4E-03 | 1.93 | 3.7E-02 |
| p-cresol sulfate | Benzoate Metabolism | 1.84 | 1.1E-02 | 0.38 | 4.4E-04 |
| 1-methylhistidine | Histidine Metabolism | 1.83 | <3.7E-04 | 0.57 | 2.3E-04 |
| 2-butenoylglycine | Fatty Acid Metabolism (Acyl Glycine) | 1.80 | 4.0E-02 | 3.41 | 6.9E-04 |
| urea | Urea cycle; Arginine and Proline Metabolism | 1.77 | <3.7E-04 | 0.72 | 1.5E-02 |
| n2,n5-diacetylornithine | Urea cycle; Arginine and Proline Metabolism | 1.77 | 4.1E-04 | 1.69 | 4.7E-02 |
| n,n,n-trimethyl-5-aminovalerate | Lysine Metabolism | 1.75 | 6.1E-03 | 0.16 | 1.8E-03 |
| xanthurenate | Tryptophan Metabolism | 1.70 | 2.0E-03 | 0.63 | 2.5E-02 |
| 6-hydroxyindole sulfate | Chemical | 1.69 | 5.0E-03 | 0.49 | 1.8E-03 |
| undecenoylcarnitine (c11:1) | Fatty Acid Metabolism (Acyl Carnitine, Monounsaturated) | 1.68 | 1.4E-03 | 0.38 | 9.6E-05 |
| tiglylcarnitine (c5:1-dc) | Leucine, Isoleucine and Valine Metabolism | 1.66 | 6.2E-04 | 0.47 | 1.2E-04 |
| trimethylamine n-oxide | Phospholipid Metabolism | 1.66 | 6.5E-03 | 0.53 | 6.4E-03 |
| guaiacol sulfate | Benzoate Metabolism | 1.65 | 7.0E-03 | 1.95 | 1.4E-02 |
| 2s,3r-dihydroxybutyrate | Fatty Acid, Dihydroxy | 1.64 | 3.9E-04 | 0.66 | 2.9E-02 |
| beta-hydroxyisovaleroylcarnitine | Leucine, Isoleucine and Valine Metabolism | 1.62 | 4.2E-04 | 0.53 | 1.5E-04 |
| picolinoylglycine | Fatty Acid Metabolism (Acyl Glycine) | 1.61 | 9.4E-04 | 0.53 | 7.1E-04 |
| isobutyrylcarnitine (c4) | Leucine, Isoleucine and Valine Metabolism | 1.60 | 5.2E-03 | 0.48 | 2.3E-04 |
| glutarylcarnitine (c5-dc) | Lysine Metabolism | 1.57 | 4.2E-03 | 0.62 | 1.0E-02 |
| trans-2-hexenoylglycine | Fatty Acid Metabolism (Acyl Glycine) | 1.57 | 2.4E-02 | 2.31 | 1.7E-03 |
| decadienedioic acid (c10:2-dc) | Fatty Acid, Dicarboxylate | 1.57 | 3.0E-02 | 0.58 | 3.1E-02 |
| 2-methylbutyrylcarnitine (c5) | Leucine, Isoleucine and Valine Metabolism | 1.55 | 1.7E-03 | 0.44 | 1.6E-04 |
| 3-carboxy-4-methyl-5-pentyl-2-furanpropionate (3-cmpfp) | Fatty Acid, Dicarboxylate | 1.55 | 1.6E-03 | 0.51 | 9.1E-05 |
| 2,3-dihydroxy-2-methylbutyrate | Leucine, Isoleucine and Valine Metabolism | 1.54 | 4.8E-03 | 1.90 | 3.3E-03 |
| quinolinate | Nicotinate and Nicotinamide Metabolism | 1.53 | 1.3E-03 | 0.71 | 2.9E-02 |
| adipoylcarnitine (c6-dc) | Fatty Acid Metabolism (Acyl Carnitine, Dicarboxylate) | 1.52 | 6.5E-03 | 0.67 | 3.4E-02 |
| 2r,3r-dihydroxybutyrate | Fatty Acid, Dihydroxy | 1.50 | 5.8E-04 | 0.51 | 9.4E-05 |
| androsterone glucuronide | Androgenic Steroids | 1.48 | 4.3E-02 | 0.50 | 1.8E-02 |
| isovalerylglycine | Leucine, Isoleucine and Valine Metabolism | 1.48 | 5.0E-03 | 0.65 | 4.0E-02 |
| indoleacetate | Tryptophan Metabolism | 1.47 | 7.6E-03 | 0.57 | 2.2E-03 |
| pentose acid | Partially Characterized Molecules | 1.47 | 4.9E-02 | 2.74 | 1.2E-03 |
| 1-stearoyl-gpg (18:0) | Lysophospholipid | 1.46 | 2.0E-02 | 0.50 | 9.5E-04 |
| 3-indoxyl sulfate | Tryptophan Metabolism | 1.45 | 1.1E-02 | 0.52 | 1.4E-03 |
| ceramide (d18:1/17:0, d17:1/18:0) | Ceramides | 1.45 | 1.3E-02 | 0.28 | 4.9E-04 |
| 1-(1-enyl-stearoyl)-2-linoleoyl-gpe (p-18:0/18:2) | Plasmalogen | 1.44 | 1.4E-03 | 0.56 | 1.2E-04 |
| n-delta-acetylornithine | Urea cycle; Arginine and Proline Metabolism | 1.44 | 3.3E-02 | 2.50 | <8.8E-05 |
| 3-hydroxybutyroylglycine | Fatty Acid Metabolism (Acyl Glycine) | 1.43 | 1.5E-02 | 2.11 | <8.8E-05 |
| margaroylcarnitine (c17) | Fatty Acid Metabolism (Acyl Carnitine, Long Chain Saturated) | 1.43 | 6.5E-03 | 0.28 | 1.3E-03 |
| sphingomyelin (d17:2/16:0, d18:2/15:0) | Sphingomyelins | 1.40 | 4.1E-03 | 0.51 | 9.9E-05 |
| 1-(1-enyl-stearoyl)-2-oleoyl-gpe (p-18:0/18:1) | Plasmalogen | 1.40 | 2.9E-03 | 0.47 | 2.8E-04 |
| n,n-dimethylalanine | Alanine and Aspartate Metabolism | 1.40 | 1.9E-02 | 0.66 | 2.6E-02 |
| beta-hydroxyisovalerate | Leucine, Isoleucine and Valine Metabolism | 1.39 | 6.1E-03 | 0.53 | 2.3E-04 |
| taurocholenate sulfate | Secondary Bile Acid Metabolism | 1.38 | 4.1E-02 | 0.55 | 6.8E-03 |
| sphingomyelin (d18:1/25:0, d19:0/24:1, d20:1/23:0, d19:1/24:0) | Sphingomyelins | 1.36 | 4.9E-02 | 0.23 | 8.9E-04 |
| 3-hydroxy-2-ethylpropionate | Leucine, Isoleucine and Valine Metabolism | 1.36 | 1.0E-02 | 0.52 | 8.9E-05 |
| kynurenine | Tryptophan Metabolism | 1.36 | 3.7E-04 | 0.79 | 2.6E-02 |
| n-formylanthranilic acid | Tryptophan Metabolism | 1.36 | 2.6E-02 | 0.58 | 2.6E-03 |
| 1-(1-enyl-palmitoyl)-2-linoleoyl-gpe (p-16:0/18:2) | Plasmalogen | 1.35 | 4.6E-03 | 0.53 | 1.1E-04 |
| sphingomyelin (d17:1/14:0, d16:1/15:0) | Sphingomyelins | 1.35 | 1.7E-02 | 0.47 | 1.0E-04 |
| n-palmitoyl-heptadecasphingosine (d17:1/16:0) | Ceramides | 1.35 | 8.3E-03 | 0.39 | 4.1E-04 |
| 1-(1-enyl-stearoyl)-2-arachidonoyl-gpe (p-18:0/20:4) | Plasmalogen | 1.34 | 1.2E-02 | 0.37 | 6.0E-04 |
| 2-methylcitrate/homocitrate | TCA Cycle | 1.34 | 5.7E-04 | 0.82 | 4.7E-02 |
| 1-oleoyl-2-linoleoyl-gpe (18:1/18:2) | Phosphatidylethanolamine (PE) | 1.34 | 2.3E-02 | 1.58 | 3.8E-02 |
| 1-(1-enyl-stearoyl)-gpe (p-18:0) | Lysoplasmalogen | 1.33 | 5.1E-03 | 0.46 | 3.0E-04 |
| formiminoglutamate | Histidine Metabolism | 1.32 | 1.6E-02 | 0.56 | 1.7E-04 |
| n-stearoyl-sphingadienine (d18:2/18:0) | Ceramides | 1.32 | 1.3E-02 | 0.50 | 1.1E-04 |
| 1-ribosyl-imidazoleacetate | Histidine Metabolism | 1.31 | 4.6E-03 | 1.49 | 2.0E-03 |
| trans-4-hydroxyproline | Urea cycle; Arginine and Proline Metabolism | 1.30 | 7.0E-03 | 0.45 | 6.7E-04 |
| n-stearoyltaurine | Endocannabinoid | 1.29 | 3.8E-02 | 0.58 | 1.0E-03 |
| cystine | Methionine, Cysteine, SAM and Taurine Metabolism | 1.28 | 4.7E-04 | 0.85 | 4.9E-02 |
| citrulline | Urea cycle; Arginine and Proline Metabolism | 1.27 | 3.8E-04 | 1.20 | 1.5E-02 |
| 1-(1-enyl-palmitoyl)-2-arachidonoyl-gpe (p-16:0/20:4) | Plasmalogen | 1.27 | 2.4E-02 | 0.42 | 3.6E-04 |
| n-acetyltyrosine | Tyrosine Metabolism | 1.26 | 6.6E-03 | 0.74 | 2.0E-02 |
| sphingomyelin (d17:1/16:0, d18:1/15:0, d16:1/17:0) | Sphingomyelins | 1.25 | 4.6E-03 | 0.57 | 1.5E-04 |
| n-acetyl-isoputreanine | Polyamine Metabolism | 1.24 | 8.4E-03 | 1.34 | 1.9E-02 |
| 3,4-dihydroxybutyrate | Fatty Acid, Dihydroxy | 1.24 | 2.0E-03 | 0.68 | 1.5E-02 |
| sphingomyelin (d18:1/21:0, d17:1/22:0, d16:1/23:0) | Sphingomyelins | 1.24 | 9.2E-03 | 0.62 | 9.3E-05 |
| sphingomyelin (d18:2/18:1) | Sphingomyelins | 1.24 | 1.2E-02 | 0.76 | 4.9E-02 |
| 1-(1-enyl-palmitoyl)-gpc (p-16:0) | Lysoplasmalogen | 1.23 | 5.7E-03 | 0.74 | 6.0E-03 |
| myristoylcarnitine (c14) | Fatty Acid Metabolism (Acyl Carnitine, Long Chain Saturated) | 1.22 | 4.3E-02 | 0.61 | 1.1E-03 |
| urate | Purine Metabolism, (Hypo)Xanthine/Inosine containing | 1.22 | 2.4E-03 | 0.85 | 4.6E-02 |
| ceramide (d18:1/20:0, d16:1/22:0, d20:1/18:0) | Ceramides | 1.21 | 4.9E-02 | 0.64 | 1.2E-03 |
| n-acetyl-beta-alanine | Pyrimidine Metabolism, Uracil containing | 1.21 | 1.1E-02 | 0.77 | 8.7E-03 |
| sphingomyelin (d18:2/23:1) | Sphingomyelins | 1.21 | 1.7E-02 | 0.69 | 6.6E-03 |
| stearoylcarnitine (c18) | Fatty Acid Metabolism (Acyl Carnitine, Long Chain Saturated) | 1.20 | 4.0E-02 | 0.67 | 1.6E-03 |
| 1-linoleoyl-gpe (18:2) | Lysophospholipid | 1.20 | 3.5E-02 | 1.38 | 2.5E-02 |
| sphingomyelin (d18:1/17:0, d17:1/18:0, d19:1/16:0) | Sphingomyelins | 1.20 | 6.8E-03 | 0.60 | 1.3E-04 |
| sphingomyelin (d18:1/14:0, d16:1/16:0) | Sphingomyelins | 1.19 | 1.6E-02 | 0.65 | 3.7E-04 |
| sphingomyelin (d18:1/19:0, d19:1/18:0) | Sphingomyelins | 1.18 | 4.1E-02 | 0.58 | 1.1E-04 |
| 1-(1-enyl-palmitoyl)-2-arachidonoyl-gpc (p-16:0/20:4) | Plasmalogen | 1.18 | 3.8E-02 | 0.59 | 8.8E-05 |
| n-palmitoyl-sphingosine (d18:1/16:0) | Ceramides | 1.18 | 1.6E-02 | 0.79 | 4.0E-02 |
| gamma-glutamylvaline | Gamma-glutamyl Amino Acid | 1.16 | 1.3E-02 | 0.82 | 3.4E-02 |
| palmitoyl-sphingosine-phosphoethanolamine (d18:1/16:0) | Ceramide PEs | 1.12 | 1.8E-02 | 1.31 | 3.6E-02 |
| sphingomyelin (d18:2/24:1, d18:1/24:2) | Sphingomyelins | 1.08 | 4.7E-02 | 1.32 | 1.6E-02 |
| ^1^Fold change represents ratio of geometric means of vegan relative to non-vegetarian dietary pattern or 90th vs 10th percentiles of creatinine | | |  |  |  |
| ^2^Adjustment for race, BMI, sex, age, batch, study, and batch*study | | | | | |

| **Supplementary Table 10.** Metabolites associated with pesco-vegetarian relative to non-vegetarian dietary patterns at FDR < 0.05 in n=139 AHS-2 participants^1^ | | | |
| --- | --- | --- | --- |
| **Metabolite** | **Subclass** | **Fold Change** | **FDR** |
| genistein sulfate | Food Component/Plant | 6.19 | 1.9E-02 |
| hydroxy-cmpf | Fatty Acid, Dicarboxylate | 5.77 | 5.0E-03 |
| daidzein sulfate (2) | Food Component/Plant | 5.43 | 2.7E-02 |
| 3-carboxy-4-methyl-5-propyl-2-furanpropanoate (cmpf) | Fatty Acid, Dicarboxylate | 4.54 | 2.1E-02 |
| s-allylcysteine | Food Component/Plant | 4.00 | 3.2E-02 |
| 4-acetylphenol sulfate | Benzoate Metabolism | 3.88 | 8.6E-03 |
| methyl glucopyranoside (alpha + beta) | Food Component/Plant | 3.86 | <2.0E-03 |
| alliin | Food Component/Plant | 3.46 | 2.4E-02 |
| branched chain 14:0 dicarboxylic acid | Fatty Acid, Dicarboxylate | 3.09 | 2.1E-02 |
| glycochenodeoxycholate 3-sulfate | Primary Bile Acid Metabolism | 3.05 | 3.1E-02 |
| alpha-cehc sulfate | Tocopherol Metabolism | 3.01 | 4.9E-02 |
| docosahexaenoylcarnitine (c22:6) | Fatty Acid Metabolism (Acyl Carnitine, Polyunsaturated) | 2.84 | 3.4E-03 |
| ethyl beta-glucopyranoside | Food Component/Plant | 2.75 | 3.1E-02 |
| 3-methyladipate | Fatty Acid, Dicarboxylate | 2.72 | 2.0E-02 |
| glutamine conjugate of c6h10o2 (2) | Partially Characterized Molecules | 2.71 | 3.3E-02 |
| 2-aminophenol sulfate | Food Component/Plant | 2.65 | 3.1E-02 |
| 14-hdohe/17-hdohe | Docosanoid | 2.62 | 2.3E-02 |
| 2-butenoylglycine | Fatty Acid Metabolism (Acyl Glycine) | 2.42 | 3.5E-02 |
| stearidonate (18:4n3) | Long Chain Polyunsaturated Fatty Acid (n3 and n6) | 2.42 | 4.9E-03 |
| s-methylcysteine | Methionine, Cysteine, SAM and Taurine Metabolism | 2.34 | 5.7E-03 |
| docosapentaenoylcarnitine (c22:5n3) | Fatty Acid Metabolism (Acyl Carnitine, Polyunsaturated) | 2.33 | 9.1E-03 |
| n-formylphenylalanine | Tyrosine Metabolism | 2.31 | 6.3E-03 |
| 2'-o-methyluridine | Pyrimidine Metabolism, Uracil containing | 2.27 | 2.0E-02 |
| gamma-cehc glucuronide | Tocopherol Metabolism | 2.21 | 2.1E-02 |
| catechol sulfate | Benzoate Metabolism | 2.18 | 2.1E-02 |
| erythritol | Food Component/Plant | 2.17 | 4.9E-02 |
| glutamine conjugate of c6h10o2 (1) | Partially Characterized Molecules | 2.16 | 1.9E-02 |
| 12,13-dihome | Fatty Acid, Dihydroxy | 2.15 | <2.0E-03 |
| pyrraline | Food Component/Plant | 2.14 | 2.0E-02 |
| eicosapentaenoate (epa; 20:5n3) | Long Chain Polyunsaturated Fatty Acid (n3 and n6) | 2.14 | 1.3E-02 |
| 1-docosahexaenoylglycerol (22:6) | Monoacylglycerol | 2.11 | 3.8E-02 |
| docosahexaenoate (dha; 22:6n3) | Long Chain Polyunsaturated Fatty Acid (n3 and n6) | 2.11 | 6.2E-03 |
| linolenoylcarnitine (c18:3) | Fatty Acid Metabolism (Acyl Carnitine, Polyunsaturated) | 2.10 | <2.0E-03 |
| 2-aminoheptanoate | Fatty Acid, Amino | 2.08 | <2.0E-03 |
| linoleoylcarnitine (c18:2) | Fatty Acid Metabolism (Acyl Carnitine, Polyunsaturated) | 2.08 | 5.7E-03 |
| linolenate [alpha or gamma; (18:3n3 or 6)] | Long Chain Polyunsaturated Fatty Acid (n3 and n6) | 2.07 | 2.0E-02 |
| guaiacol sulfate | Benzoate Metabolism | 2.00 | 2.7E-02 |
| octadecadienedioate (c18:2-dc) | Fatty Acid, Dicarboxylate | 1.97 | 7.7E-03 |
| gamma-glutamylglutamate | Gamma-glutamyl Amino Acid | 1.94 | 1.2E-02 |
| guanidinoacetate | Creatine Metabolism | 1.94 | 7.9E-03 |
| s-methylcysteine sulfoxide | Methionine, Cysteine, SAM and Taurine Metabolism | 1.92 | 2.0E-02 |
| dopamine 4-sulfate | Tyrosine Metabolism | 1.89 | 3.9E-02 |
| 2,6-dihydroxybenzoic acid | Drug - Topical Agents | 1.86 | 2.5E-02 |
| 9,10-dihome | Fatty Acid, Dihydroxy | 1.86 | 1.3E-02 |
| gamma-cehc | Tocopherol Metabolism | 1.83 | 4.1E-02 |
| dopamine 3-o-sulfate | Tyrosine Metabolism | 1.80 | 3.0E-02 |
| hypotaurine | Methionine, Cysteine, SAM and Taurine Metabolism | 1.74 | 1.6E-02 |
| n-delta-acetylornithine | Urea cycle; Arginine and Proline Metabolism | 1.73 | 4.2E-02 |
| hexadecadienoate (16:2n6) | Long Chain Polyunsaturated Fatty Acid (n3 and n6) | 1.67 | 4.4E-02 |
| 5-methyluridine (ribothymidine) | Pyrimidine Metabolism, Uracil containing | 1.66 | 2.7E-02 |
| n-linoleoyltaurine | Endocannabinoid | 1.65 | 4.9E-02 |
| dihomo-linoleoylcarnitine (c20:2) | Fatty Acid Metabolism (Acyl Carnitine, Polyunsaturated) | 1.64 | 3.5E-02 |
| gamma-glutamyltryptophan | Gamma-glutamyl Amino Acid | 1.63 | 1.6E-02 |
| 2,3-dihydroxy-2-methylbutyrate | Leucine, Isoleucine and Valine Metabolism | 1.63 | 4.9E-02 |
| lactate | Glycolysis, Gluconeogenesis, and Pyruvate Metabolism | 1.62 | 2.0E-02 |
| s-adenosylhomocysteine (sah) | Methionine, Cysteine, SAM and Taurine Metabolism | 1.62 | 3.0E-02 |
| 13-hode + 9-hode | Fatty Acid, Monohydroxy | 1.60 | 4.9E-02 |
| linoleate (18:2n6) | Long Chain Polyunsaturated Fatty Acid (n3 and n6) | 1.60 | 4.1E-02 |
| gamma-glutamylcitrulline | Gamma-glutamyl Amino Acid | 1.58 | 2.7E-02 |
| butyrate/isobutyrate (4:0) | Short Chain Fatty Acid | 1.58 | 4.0E-02 |
| 3-hydroxybutyroylglycine | Fatty Acid Metabolism (Acyl Glycine) | 1.57 | 5.0E-02 |
| cis-4-decenoate (10:1n6) | Medium Chain Fatty Acid | 1.53 | 4.2E-02 |
| malate | TCA Cycle | 1.51 | 1.7E-02 |
| oleoylcarnitine (c18:1) | Fatty Acid Metabolism (Acyl Carnitine, Monounsaturated) | 1.50 | 4.9E-02 |
| octadecenedioate (c18:1-dc) | Fatty Acid, Dicarboxylate | 1.47 | 4.9E-02 |
| n-acetylmethionine | Methionine, Cysteine, SAM and Taurine Metabolism | 1.45 | 4.2E-02 |
| uridine | Pyrimidine Metabolism, Uracil containing | 1.42 | 3.1E-02 |
| caproate (6:0) | Medium Chain Fatty Acid | 1.42 | 1.7E-02 |
| 3-methoxytyrosine | Tyrosine Metabolism | 1.41 | 1.6E-02 |
| s-1-pyrroline-5-carboxylate | Glutamate Metabolism | 1.41 | 4.3E-02 |
| cysteine | Methionine, Cysteine, SAM and Taurine Metabolism | 1.40 | 4.9E-02 |
| 6-bromotryptophan | Tryptophan Metabolism | 1.39 | 5.0E-02 |
| 1-stearoyl-2-linoleoyl-gpi (18:0/18:2) | Phosphatidylinositol (PI) | 1.39 | 3.1E-02 |
| thyroxine | Tyrosine Metabolism | 1.35 | 5.0E-02 |
| proline | Urea cycle; Arginine and Proline Metabolism | 1.35 | 3.1E-02 |
| 16-hydroxypalmitate | Fatty Acid, Monohydroxy | 1.34 | 4.8E-02 |
| gamma-glutamylglutamine | Gamma-glutamyl Amino Acid | 1.31 | 4.0E-02 |
| ornithine | Urea cycle; Arginine and Proline Metabolism | 1.30 | 4.9E-02 |
| 1-palmitoyl-gpc (16:0) | Lysophospholipid | 0.82 | 2.0E-02 |
| 1-(1-enyl-palmitoyl)-2-oleoyl-gpc (p-16:0/18:1) | Plasmalogen | 0.79 | 3.7E-02 |
| 1-linoleoyl-gpc (18:2) | Lysophospholipid | 0.78 | 1.8E-02 |
| 1-palmitoyl-2-linoleoyl-gpc (16:0/18:2) | Phosphatidylcholine (PC) | 0.78 | 3.0E-02 |
| n-acetyl-beta-alanine | Pyrimidine Metabolism, Uracil containing | 0.78 | 2.2E-02 |
| 1-stearoyl-gpe (18:0) | Lysophospholipid | 0.78 | 4.0E-02 |
| 1-(1-enyl-palmitoyl)-2-palmitoyl-gpc (p-16:0/16:0) | Plasmalogen | 0.77 | 1.7E-02 |
| palmitoyl sphingomyelin (d18:1/16:0) | Sphingomyelins | 0.76 | 1.2E-02 |
| 1-palmitoyl-2-dihomo-linolenoyl-gpc (16:0/20:3n3 or 6) | Phosphatidylcholine (PC) | 0.75 | 1.5E-02 |
| sphingomyelin (d18:2/24:1, d18:1/24:2) | Sphingomyelins | 0.75 | 1.3E-02 |
| 1-linoleoyl-2-arachidonoyl-gpc (18:2/20:4n6) | Phosphatidylcholine (PC) | 0.75 | 2.3E-02 |
| 1-oleoyl-gpc (18:1) | Lysophospholipid | 0.75 | 1.9E-03 |
| sphingomyelin (d18:1/22:1, d18:2/22:0, d16:1/24:1) | Sphingomyelins | 0.74 | 1.3E-02 |
| adenine | Purine Metabolism, Adenine containing | 0.74 | 1.9E-02 |
| cholesterol | Sterol | 0.73 | 4.3E-03 |
| palmitoyl-sphingosine-phosphoethanolamine (d18:1/16:0) | Ceramide PEs | 0.73 | 1.3E-02 |
| glycerophosphoethanolamine | Phospholipid Metabolism | 0.72 | 1.3E-02 |
| imidazole lactate | Histidine Metabolism | 0.72 | 8.7E-03 |
| 1-(1-enyl-palmitoyl)-2-palmitoleoyl-gpc (p-16:0/16:1) | Plasmalogen | 0.72 | 1.9E-02 |
| myristoyl dihydrosphingomyelin (d18:0/14:0) | Dihydrosphingomyelins | 0.71 | 2.3E-02 |
| sphingomyelin (d18:1/22:2, d18:2/22:1, d16:1/24:2) | Sphingomyelins | 0.71 | 1.3E-02 |
| 1-palmitoyl-2-oleoyl-gpc (16:0/18:1) | Phosphatidylcholine (PC) | 0.71 | 2.7E-03 |
| 1-stearoyl-2-oleoyl-gpc (18:0/18:1) | Phosphatidylcholine (PC) | 0.71 | 3.1E-03 |
| sarcosine | Glycine, Serine and Threonine Metabolism | 0.71 | 2.3E-02 |
| sphingomyelin (d18:2/14:0, d18:1/14:1) | Sphingomyelins | 0.71 | 4.0E-02 |
| quinolinate | Nicotinate and Nicotinamide Metabolism | 0.71 | 4.3E-02 |
| 1-stearoyl-2-arachidonoyl-gpc (18:0/20:4) | Phosphatidylcholine (PC) | 0.70 | 3.4E-03 |
| tricosanoyl sphingomyelin (d18:1/23:0) | Sphingomyelins | 0.69 | 4.3E-03 |
| sphingomyelin (d18:2/23:0, d18:1/23:1, d17:1/24:1) | Sphingomyelins | 0.69 | 1.9E-03 |
| n6,n6,n6-trimethyllysine | Lysine Metabolism | 0.69 | 1.9E-02 |
| 6-oxopiperidine-2-carboxylate | Lysine Metabolism | 0.69 | 3.7E-02 |
| stearoyl sphingomyelin (d18:1/18:0) | Sphingomyelins | 0.69 | 5.5E-03 |
| 1-palmitoleoyl-gpc (16:1) | Lysophospholipid | 0.69 | 3.1E-03 |
| urea | Urea cycle; Arginine and Proline Metabolism | 0.68 | 9.4E-03 |
| ribonate | Pentose Metabolism | 0.68 | 1.3E-02 |
| creatine | Creatine Metabolism | 0.68 | 1.2E-02 |
| 1-(1-enyl-oleoyl)-gpe (p-18:1) | Lysoplasmalogen | 0.68 | 4.1E-03 |
| sphingomyelin (d18:1/21:0, d17:1/22:0, d16:1/23:0) | Sphingomyelins | 0.67 | 3.0E-03 |
| sphingomyelin (d18:2/16:0, d18:1/16:1) | Sphingomyelins | 0.67 | 5.2E-04 |
| retinol (vitamin a) | Vitamin A Metabolism | 0.67 | 2.3E-02 |
| 5-methylthioadenosine (mta) | Polyamine Metabolism | 0.67 | 2.1E-04 |
| beta-hydroxyisovaleroylcarnitine | Leucine, Isoleucine and Valine Metabolism | 0.67 | 7.5E-03 |
| sphingomyelin (d18:1/14:0, d16:1/16:0) | Sphingomyelins | 0.67 | 3.0E-03 |
| homocitrulline | Urea cycle; Arginine and Proline Metabolism | 0.67 | 4.9E-02 |
| 1-palmitoyl-2-arachidonoyl-gpc (16:0/20:4n6) | Phosphatidylcholine (PC) | 0.67 | 7.9E-04 |
| palmitoylcholine | Fatty Acid Metabolism (Acyl Choline) | 0.66 | 3.6E-02 |
| sphingomyelin (d18:2/21:0, d16:2/23:0) | Sphingomyelins | 0.66 | 2.9E-03 |
| isobutyrylcarnitine (c4) | Leucine, Isoleucine and Valine Metabolism | 0.65 | 4.6E-02 |
| n-acetylcarnosine | Histidine Metabolism | 0.65 | 4.1E-02 |
| 3-aminoisobutyrate | Pyrimidine Metabolism, Thymine containing | 0.65 | 4.0E-02 |
| 1-(1-enyl-palmitoyl)-gpe (p-16:0) | Lysoplasmalogen | 0.65 | 1.2E-03 |
| sphingomyelin (d18:1/20:2, d18:2/20:1, d16:1/22:2) | Sphingomyelins | 0.65 | 7.0E-03 |
| 1-(1-enyl-palmitoyl)-2-linoleoyl-gpc (p-16:0/18:2) | Plasmalogen | 0.64 | 8.1E-04 |
| behenoyl sphingomyelin (d18:1/22:0) | Sphingomyelins | 0.64 | 6.0E-03 |
| sphingomyelin (d18:1/18:1, d18:2/18:0) | Sphingomyelins | 0.63 | 5.3E-04 |
| erythronate | Aminosugar Metabolism | 0.63 | 3.7E-02 |
| stearoylcholine | Fatty Acid Metabolism (Acyl Choline) | 0.62 | 2.0E-02 |
| 1-(1-enyl-palmitoyl)-gpc (p-16:0) | Lysoplasmalogen | 0.62 | 2.4E-04 |
| 1-arachidonoyl-gpe (20:4n6) | Lysophospholipid | 0.62 | 3.8E-04 |
| 1-stearoyl-2-arachidonoyl-gpe (18:0/20:4) | Phosphatidylethanolamine (PE) | 0.61 | 3.4E-03 |
| trans-4-hydroxyproline | Urea cycle; Arginine and Proline Metabolism | 0.61 | 3.2E-04 |
| ceramide (d18:1/17:0, d17:1/18:0) | Ceramides | 0.60 | 1.5E-02 |
| phenylacetylglutamine | Acetylated Peptides | 0.60 | 4.7E-02 |
| sphingomyelin (d18:1/19:0, d19:1/18:0) | Sphingomyelins | 0.60 | 5.9E-04 |
| 1-oleoyl-2-arachidonoyl-gpe (18:1/20:4) | Phosphatidylethanolamine (PE) | 0.59 | 1.6E-02 |
| hexanoylcarnitine (c6) | Fatty Acid Metabolism (Acyl Carnitine, Medium Chain) | 0.59 | 2.3E-02 |
| n-stearoyl-sphingosine (d18:1/18:0) | Ceramides | 0.59 | 1.2E-02 |
| oleoylcholine | Fatty Acid Metabolism (Acyl Choline) | 0.58 | 6.3E-03 |
| docosapentaenoate (n6 dpa; 22:5n6) | Long Chain Polyunsaturated Fatty Acid (n3 and n6) | 0.58 | 1.5E-02 |
| sphingomyelin (d18:2/23:1) | Sphingomyelins | 0.58 | 4.1E-04 |
| sphingomyelin (d18:1/17:0, d17:1/18:0, d19:1/16:0) | Sphingomyelins | 0.57 | 4.4E-04 |
| 1-(1-enyl-palmitoyl)-2-oleoyl-gpe (p-16:0/18:1) | Plasmalogen | 0.57 | 2.6E-04 |
| n-stearoyl-sphinganine (d18:0/18:0) | Dihydroceramides | 0.57 | 2.5E-02 |
| 1-palmitoyl-2-arachidonoyl-gpe (16:0/20:4) | Phosphatidylethanolamine (PE) | 0.57 | 2.1E-03 |
| fructosyllysine | Lysine Metabolism | 0.57 | 2.5E-02 |
| sphingomyelin (d17:1/14:0, d16:1/15:0) | Sphingomyelins | 0.57 | 3.6E-03 |
| sphingomyelin (d18:1/24:1, d18:2/24:0) | Sphingomyelins | 0.57 | 5.0E-03 |
| n-methylpipecolate | Bacterial/Fungal | 0.55 | 2.1E-02 |
| palmitoloelycholine | Fatty Acid Metabolism (Acyl Choline) | 0.55 | 1.1E-02 |
| dihomo-linolenoyl-choline | Fatty Acid Metabolism (Acyl Choline) | 0.55 | 9.5E-03 |
| xanthurenate | Tryptophan Metabolism | 0.55 | 8.9E-03 |
| guanosine | Purine Metabolism, Guanine containing | 0.55 | 3.7E-02 |
| glucuronate | Aminosugar Metabolism | 0.55 | 6.3E-03 |
| 1-palmitoyl-2-oleoyl-gpe (16:0/18:1) | Phosphatidylethanolamine (PE) | 0.54 | 2.0E-03 |
| oleoyl-arachidonoyl-glycerol (18:1/20:4) [2] | Diacylglycerol | 0.54 | 4.4E-03 |
| 1-palmitoyl-2-palmitoleoyl-gpc (16:0/16:1) | Phosphatidylcholine (PC) | 0.54 | 1.4E-03 |
| arachidonoylcholine | Fatty Acid Metabolism (Acyl Choline) | 0.54 | 3.0E-03 |
| 1-(1-enyl-stearoyl)-2-linoleoyl-gpe (p-18:0/18:2) | Plasmalogen | 0.53 | 4.8E-04 |
| sphingomyelin (d17:1/16:0, d18:1/15:0, d16:1/17:0) | Sphingomyelins | 0.53 | 6.3E-04 |
| decadienedioic acid (c10:2-dc) | Fatty Acid, Dicarboxylate | 0.52 | 2.0E-02 |
| 3-bromo-5-chloro-2,6-dihydroxybenzoic acid | Chemical | 0.52 | 7.6E-03 |
| sphingomyelin (d18:0/18:0, d19:0/17:0) | Dihydrosphingomyelins | 0.52 | 1.4E-03 |
| 4-hydroxyglutamate | Glutamate Metabolism | 0.51 | 3.6E-03 |
| 1-arachidonoyl-gpc (20:4n6) | Lysophospholipid | 0.51 | 5.2E-04 |
| (16 or 17)-methylstearate (a19:0 or i19:0) | Fatty Acid, Branched | 0.51 | 1.4E-03 |
| acetoacetate | Ketone Bodies | 0.51 | 3.7E-02 |
| sphingomyelin (d18:2/18:1) | Sphingomyelins | 0.50 | 3.4E-04 |
| sphingomyelin (d17:2/16:0, d18:2/15:0) | Sphingomyelins | 0.50 | 2.3E-04 |
| gluconate | Food Component/Plant | 0.50 | 3.1E-03 |
| phenylacetylglutamate | Acetylated Peptides | 0.49 | 4.0E-02 |
| 1-(1-enyl-stearoyl)-gpe (p-18:0) | Lysoplasmalogen | 0.49 | 7.1E-04 |
| oleoyl-linoleoyl-glycerol (18:1/18:2) [2] | Diacylglycerol | 0.49 | 1.9E-02 |
| cytidine | Pyrimidine Metabolism, Cytidine containing | 0.48 | 3.6E-03 |
| p-cresol sulfate | Benzoate Metabolism | 0.48 | 1.4E-02 |
| 1-oleoylglycerol (18:1) | Monoacylglycerol | 0.47 | 1.8E-03 |
| 1-(1-enyl-palmitoyl)-2-linoleoyl-gpe (p-16:0/18:2) | Plasmalogen | 0.46 | 5.7E-04 |
| n-acetyltaurine | Methionine, Cysteine, SAM and Taurine Metabolism | 0.45 | 5.7E-04 |
| orotidine | Pyrimidine Metabolism, Orotate containing | 0.45 | 8.7E-03 |
| mannonate | Food Component/Plant | 0.45 | 1.3E-03 |
| arabonate/xylonate | Pentose Metabolism | 0.45 | 2.8E-02 |
| inosine | Purine Metabolism, (Hypo)Xanthine/Inosine containing | 0.44 | 1.9E-02 |
| n,n,n-trimethyl-5-aminovalerate | Lysine Metabolism | 0.43 | 2.9E-04 |
| sulfate of piperine metabolite c16h19no3 (2) | Food Component/Plant | 0.42 | 2.6E-02 |
| sphingomyelin (d18:1/25:0, d19:0/24:1, d20:1/23:0, d19:1/24:0) | Sphingomyelins | 0.42 | 4.1E-04 |
| (12 or 13)-methylmyristate (a15:0 or i15:0) | Fatty Acid, Branched | 0.41 | 4.3E-03 |
| 1-(1-enyl-stearoyl)-2-oleoyl-gpe (p-18:0/18:1) | Plasmalogen | 0.41 | 1.1E-03 |
| 1-(1-enyl-palmitoyl)-2-arachidonoyl-gpe (p-16:0/20:4) | Plasmalogen | 0.40 | 9.5E-04 |
| gulonate | Ascorbate and Aldarate Metabolism | 0.40 | 1.1E-02 |
| 1-(1-enyl-palmitoyl)-2-arachidonoyl-gpc (p-16:0/20:4) | Plasmalogen | 0.39 | 1.4E-03 |
| 1-(1-enyl-stearoyl)-2-arachidonoyl-gpe (p-18:0/20:4) | Plasmalogen | 0.37 | 1.9E-03 |
| glycerophosphoinositol | Phospholipid Metabolism | 0.35 | 2.5E-02 |
| docosatrienoate (22:3n6) | Long Chain Polyunsaturated Fatty Acid (n3 and n6) | 0.34 | 5.4E-03 |
| hydantoin-5-propionate | Histidine Metabolism | 0.32 | 5.5E-04 |
| 3-(methylthio)acetaminophen sulfate | Drug - Analgesics, Anesthetics | 0.31 | 4.1E-02 |
| 1-methylurate | Xanthine Metabolism | 0.31 | 6.2E-03 |
| n-acetyl-1-methylhistidine | Histidine Metabolism | 0.30 | 2.4E-03 |
| 1,3,7-trimethylurate | Xanthine Metabolism | 0.27 | 1.6E-03 |
| ectoine | Chemical | 0.25 | 3.0E-04 |
| 1,3-dimethylurate | Xanthine Metabolism | 0.24 | 5.0E-04 |
| 1-methylxanthine | Xanthine Metabolism | 0.13 | 2.5E-04 |
| quinate | Food Component/Plant | 0.13 | 1.3E-03 |
| 1,7-dimethylurate | Xanthine Metabolism | 0.11 | 2.2E-04 |
| paraxanthine | Xanthine Metabolism | 0.10 | 3.6E-04 |
| theophylline | Xanthine Metabolism | 0.09 | 2.7E-04 |
| 1-methyl-5-imidazoleacetate | Histidine Metabolism | 0.06 | 5.7E-03 |
| caffeine | Xanthine Metabolism | 0.06 | 2.1E-03 |
| 3-methylhistidine | Histidine Metabolism | 0.06 | 2.9E-03 |
| 5-acetylamino-6-amino-3-methyluracil | Xanthine Metabolism | 0.03 | 8.1E-04 |

^1^Adjustment for race, BMI, sex, age, batch, study, and batch*study

|  |  |
| --- | --- |
|  | |

| **Supplementary Table 11.** Plasma metabolites associated with both plasma creatinine and pesco-vegetarian (relative to non-vegetarian) dietary pattern at FDR < 0.05 ^1,2^ | | | | | |
| --- | --- | --- | --- | --- | --- |
|  |  | **Creatinine** | | **Pesco-vegetarian** | |
| **Metabolite** | **Subclass** | **Fold Change** | **FDR** | **Fold Change** | **FDR** |
| 1-methyl-5-imidazoleacetate | Histidine Metabolism | 4.29 | 7.9E-04 | 0.06 | 5.7E-03 |
| 3-methylhistidine | Histidine Metabolism | 3.96 | 3.2E-03 | 0.06 | 2.9E-03 |
| 3-carboxy-4-methyl-5-propyl-2-furanpropanoate (cmpf) | Fatty Acid, Dicarboxylate | 3.72 | 2.8E-02 | 4.54 | 2.1E-02 |
| n-acetyl-1-methylhistidine | Histidine Metabolism | 3.35 | 6.0E-04 | 0.30 | 2.4E-03 |
| quinate | Food Component/Plant | 3.18 | 4.9E-02 | 0.13 | 1.3E-03 |
| sulfate of piperine metabolite c16h19no3 (2) | Food Component/Plant | 3.04 | 2.7E-03 | 0.42 | 2.6E-02 |
| ectoine | Chemical | 2.79 | 9.8E-04 | 0.25 | 3.0E-04 |
| homocitrulline | Urea cycle; Arginine and Proline Metabolism | 2.23 | <4.0E-04 | 0.67 | 4.9E-02 |
| phenylacetylglutamate | Acetylated Peptides | 2.18 | 4.6E-03 | 0.49 | 4.0E-02 |
| n-acetyltaurine | Methionine, Cysteine, SAM and Taurine Metabolism | 1.97 | <4.0E-04 | 0.45 | 5.7E-04 |
| n-methylpipecolate | Bacterial/Fungal | 1.95 | 1.7E-03 | 0.55 | 2.1E-02 |
| hydantoin-5-propionate | Histidine Metabolism | 1.94 | 1.4E-03 | 0.32 | 5.5E-04 |
| p-cresol sulfate | Benzoate Metabolism | 1.84 | 1.1E-02 | 0.48 | 1.4E-02 |
| n6,n6,n6-trimethyllysine | Lysine Metabolism | 1.81 | <4.0E-04 | 0.69 | 1.9E-02 |
| 2-butenoylglycine | Fatty Acid Metabolism (Acyl Glycine) | 1.80 | 4.0E-02 | 2.42 | 3.5E-02 |
| urea | Urea cycle; Arginine and Proline Metabolism | 1.77 | <4.0E-04 | 0.68 | 9.4E-03 |
| n,n,n-trimethyl-5-aminovalerate | Lysine Metabolism | 1.75 | 6.1E-03 | 0.43 | 2.9E-04 |
| xanthurenate | Tryptophan Metabolism | 1.70 | 2.0E-03 | 0.55 | 8.9E-03 |
| gulonate | Ascorbate and Aldarate Metabolism | 1.70 | <4.0E-04 | 0.40 | 1.1E-02 |
| guaiacol sulfate | Benzoate Metabolism | 1.65 | 7.0E-03 | 2.00 | 2.7E-02 |
| glutamine conjugate of c6h10o2 (1) | Partially Characterized Molecules | 1.63 | 1.0E-02 | 2.16 | 1.9E-02 |
| beta-hydroxyisovaleroylcarnitine | Leucine, Isoleucine and Valine Metabolism | 1.62 | 4.2E-04 | 0.67 | 7.5E-03 |
| glycerophosphoinositol | Phospholipid Metabolism | 1.62 | 1.2E-02 | 0.35 | 2.5E-02 |
| isobutyrylcarnitine (c4) | Leucine, Isoleucine and Valine Metabolism | 1.60 | 5.2E-03 | 0.65 | 4.6E-02 |
| decadienedioic acid (c10:2-dc) | Fatty Acid, Dicarboxylate | 1.57 | 3.0E-02 | 0.52 | 2.0E-02 |
| 2,3-dihydroxy-2-methylbutyrate | Leucine, Isoleucine and Valine Metabolism | 1.54 | 4.8E-03 | 1.63 | 4.9E-02 |
| gamma-cehc glucuronide | Tocopherol Metabolism | 1.54 | 8.7E-03 | 2.21 | 2.1E-02 |
| quinolinate | Nicotinate and Nicotinamide Metabolism | 1.53 | 1.3E-03 | 0.71 | 4.3E-02 |
| dopamine 4-sulfate | Tyrosine Metabolism | 1.52 | 5.9E-03 | 1.89 | 3.9E-02 |
| n-acetylcarnosine | Histidine Metabolism | 1.49 | 3.5E-03 | 0.65 | 4.1E-02 |
| retinol (vitamin a) | Vitamin A Metabolism | 1.48 | <4.0E-04 | 0.67 | 2.3E-02 |
| orotidine | Pyrimidine Metabolism, Orotate containing | 1.48 | 4.1E-04 | 0.45 | 8.7E-03 |
| 3-aminoisobutyrate | Pyrimidine Metabolism, Thymine containing | 1.47 | 1.2E-02 | 0.65 | 4.0E-02 |
| erythritol | Food Component/Plant | 1.45 | 1.3E-03 | 2.17 | 4.9E-02 |
| ceramide (d18:1/17:0, d17:1/18:0) | Ceramides | 1.45 | 1.3E-02 | 0.60 | 1.5E-02 |
| 1-(1-enyl-stearoyl)-2-linoleoyl-gpe (p-18:0/18:2) | Plasmalogen | 1.44 | 1.4E-03 | 0.53 | 4.8E-04 |
| n-delta-acetylornithine | Urea cycle; Arginine and Proline Metabolism | 1.44 | 3.3E-02 | 1.73 | 4.2E-02 |
| 3-hydroxybutyroylglycine | Fatty Acid Metabolism (Acyl Glycine) | 1.43 | 1.5E-02 | 1.57 | 5.0E-02 |
| arabonate/xylonate | Pentose Metabolism | 1.41 | 3.1E-03 | 0.45 | 2.8E-02 |
| sphingomyelin (d17:2/16:0, d18:2/15:0) | Sphingomyelins | 1.40 | 4.1E-03 | 0.50 | 2.3E-04 |
| 1-(1-enyl-stearoyl)-2-oleoyl-gpe (p-18:0/18:1) | Plasmalogen | 1.40 | 2.9E-03 | 0.41 | 1.1E-03 |
| glucuronate | Aminosugar Metabolism | 1.40 | 4.6E-03 | 0.55 | 6.3E-03 |
| s-adenosylhomocysteine (sah) | Methionine, Cysteine, SAM and Taurine Metabolism | 1.39 | 4.9E-04 | 1.62 | 3.0E-02 |
| imidazole lactate | Histidine Metabolism | 1.37 | 1.3E-03 | 0.72 | 8.7E-03 |
| sphingomyelin (d18:1/25:0, d19:0/24:1, d20:1/23:0, d19:1/24:0) | Sphingomyelins | 1.36 | 4.9E-02 | 0.42 | 4.1E-04 |
| mannonate | Food Component/Plant | 1.36 | 9.6E-04 | 0.45 | 1.3E-03 |
| 1-(1-enyl-palmitoyl)-2-linoleoyl-gpe (p-16:0/18:2) | Plasmalogen | 1.35 | 4.6E-03 | 0.46 | 5.7E-04 |
| sphingomyelin (d17:1/14:0, d16:1/15:0) | Sphingomyelins | 1.35 | 1.7E-02 | 0.57 | 3.6E-03 |
| 1-(1-enyl-stearoyl)-2-arachidonoyl-gpe (p-18:0/20:4) | Plasmalogen | 1.34 | 1.2E-02 | 0.37 | 1.9E-03 |
| erythronate | Aminosugar Metabolism | 1.33 | <4.0E-04 | 0.63 | 3.7E-02 |
| dopamine 3-o-sulfate | Tyrosine Metabolism | 1.33 | 2.2E-02 | 1.80 | 3.0E-02 |
| 1-(1-enyl-stearoyl)-gpe (p-18:0) | Lysoplasmalogen | 1.33 | 5.1E-03 | 0.49 | 7.1E-04 |
| trans-4-hydroxyproline | Urea cycle; Arginine and Proline Metabolism | 1.30 | 7.0E-03 | 0.61 | 3.2E-04 |
| 5-methylthioadenosine (mta) | Polyamine Metabolism | 1.27 | 1.5E-03 | 0.67 | 2.1E-04 |
| 1-(1-enyl-palmitoyl)-2-arachidonoyl-gpe (p-16:0/20:4) | Plasmalogen | 1.27 | 2.4E-02 | 0.40 | 9.5E-04 |
| 1-(1-enyl-palmitoyl)-2-linoleoyl-gpc (p-16:0/18:2) | Plasmalogen | 1.26 | 1.6E-03 | 0.64 | 8.1E-04 |
| gluconate | Food Component/Plant | 1.26 | 4.8E-03 | 0.50 | 3.1E-03 |
| sphingomyelin (d17:1/16:0, d18:1/15:0, d16:1/17:0) | Sphingomyelins | 1.25 | 4.6E-03 | 0.53 | 6.3E-04 |
| sphingomyelin (d18:2/23:0, d18:1/23:1, d17:1/24:1) | Sphingomyelins | 1.25 | 1.5E-03 | 0.69 | 1.9E-03 |
| ribonate | Pentose Metabolism | 1.24 | 1.9E-02 | 0.68 | 1.3E-02 |
| sphingomyelin (d18:2/14:0, d18:1/14:1) | Sphingomyelins | 1.24 | 2.5E-02 | 0.71 | 4.0E-02 |
| sphingomyelin (d18:1/21:0, d17:1/22:0, d16:1/23:0) | Sphingomyelins | 1.24 | 9.2E-03 | 0.67 | 3.0E-03 |
| sphingomyelin (d18:2/18:1) | Sphingomyelins | 1.24 | 1.2E-02 | 0.50 | 3.4E-04 |
| sphingomyelin (d18:2/21:0, d16:2/23:0) | Sphingomyelins | 1.24 | 4.9E-03 | 0.66 | 2.9E-03 |
| 1-(1-enyl-palmitoyl)-gpc (p-16:0) | Lysoplasmalogen | 1.23 | 5.7E-03 | 0.62 | 2.4E-04 |
| n-acetyl-beta-alanine | Pyrimidine Metabolism, Uracil containing | 1.21 | 1.1E-02 | 0.78 | 2.2E-02 |
| sphingomyelin (d18:2/23:1) | Sphingomyelins | 1.21 | 1.7E-02 | 0.58 | 4.1E-04 |
| sphingomyelin (d18:1/17:0, d17:1/18:0, d19:1/16:0) | Sphingomyelins | 1.20 | 6.8E-03 | 0.57 | 4.4E-04 |
| cholesterol | Sterol | 1.19 | 6.9E-03 | 0.73 | 4.3E-03 |
| sphingomyelin (d18:1/14:0, d16:1/16:0) | Sphingomyelins | 1.19 | 1.6E-02 | 0.67 | 3.0E-03 |
| sphingomyelin (d18:1/19:0, d19:1/18:0) | Sphingomyelins | 1.18 | 4.1E-02 | 0.60 | 5.9E-04 |
| 1-(1-enyl-palmitoyl)-2-arachidonoyl-gpc (p-16:0/20:4) | Plasmalogen | 1.18 | 3.8E-02 | 0.39 | 1.4E-03 |
| sphingomyelin (d18:2/16:0, d18:1/16:1) | Sphingomyelins | 1.18 | 1.3E-03 | 0.67 | 5.2E-04 |
| tricosanoyl sphingomyelin (d18:1/23:0) | Sphingomyelins | 1.18 | 1.5E-02 | 0.69 | 4.3E-03 |
| 1-(1-enyl-palmitoyl)-2-oleoyl-gpc (p-16:0/18:1) | Plasmalogen | 1.16 | 1.1E-02 | 0.79 | 3.7E-02 |
| 1-stearoyl-gpe (18:0) | Lysophospholipid | 1.16 | 3.4E-02 | 0.78 | 4.0E-02 |
| sphingomyelin (d18:1/22:1, d18:2/22:0, d16:1/24:1) | Sphingomyelins | 1.16 | 1.7E-03 | 0.74 | 1.3E-02 |
| 1-(1-enyl-palmitoyl)-2-oleoyl-gpe (p-16:0/18:1) | Plasmalogen | 1.16 | 3.7E-02 | 0.57 | 2.6E-04 |
| sphingomyelin (d18:1/18:1, d18:2/18:0) | Sphingomyelins | 1.14 | 1.3E-02 | 0.63 | 5.3E-04 |
| glycerophosphoethanolamine | Phospholipid Metabolism | 1.14 | 1.8E-02 | 0.72 | 1.3E-02 |
| 1-linoleoyl-gpc (18:2) | Lysophospholipid | 1.13 | 2.2E-02 | 0.78 | 1.8E-02 |
| behenoyl sphingomyelin (d18:1/22:0) | Sphingomyelins | 1.12 | 2.4E-02 | 0.64 | 6.0E-03 |
| palmitoyl-sphingosine-phosphoethanolamine (d18:1/16:0) | Ceramide PEs | 1.12 | 1.8E-02 | 0.73 | 1.3E-02 |
| palmitoyl sphingomyelin (d18:1/16:0) | Sphingomyelins | 1.10 | 2.4E-03 | 0.76 | 1.2E-02 |
| sphingomyelin (d18:1/24:1, d18:2/24:0) | Sphingomyelins | 1.09 | 3.5E-02 | 0.57 | 5.0E-03 |
| sphingomyelin (d18:2/24:1, d18:1/24:2) | Sphingomyelins | 1.08 | 4.7E-02 | 0.75 | 1.3E-02 |
| 1-palmitoyl-2-linoleoyl-gpc (16:0/18:2) | Phosphatidylcholine (PC) | 1.07 | 1.6E-02 | 0.78 | 3.0E-02 |
| ^1^Fold change represents ratio of geometric means of pesco-vegetarian relative to non-vegetarian dietary patterns or 90th vs 10th percentiles of creatinine | | | | |  |
| ^2^Adjustment for race, BMI, sex, age, batch, study, and batch*study | | | | | |

| **Supplementary Table 12.** Metabolites associated with vegan relative to pesco-vegetarian dietary patterns at FDR < 0.05 in n=139 AHS-2 participants^1^ | | | |
| --- | --- | --- | --- |
| **Metabolite** | **Subclass** | **Fold Change** | **FDR** |
| gentisate | Tyrosine Metabolism | 4.80 | <3.0E-04 |
| linoleoyl-linolenoyl-glycerol (18:2/18:3) [2] | Diacylglycerol | 4.56 | 1.8E-03 |
| oleoyl-linoleoyl-glycerol (18:1/18:2) [2] | Diacylglycerol | 3.77 | <3.0E-04 |
| pentose acid | Partially Characterized Molecules | 3.58 | 6.3E-04 |
| s-methylmethionine | Methionine, Cysteine, SAM and Taurine Metabolism | 3.25 | 2.1E-02 |
| 1-oleoyl-2-arachidonoyl-gpe (18:1/20:4) | Phosphatidylethanolamine (PE) | 2.96 | <3.0E-04 |
| beta-cryptoxanthin | Vitamin A Metabolism | 2.83 | 5.9E-04 |
| ectoine | Chemical | 2.76 | 6.2E-03 |
| indolepropionate | Tryptophan Metabolism | 2.73 | 8.5E-03 |
| carboxyethyl-gaba | Glutamate Metabolism | 2.70 | 2.6E-03 |
| gulonate | Ascorbate and Aldarate Metabolism | 2.60 | 1.9E-02 |
| palmitoyl-linoleoyl-glycerol (16:0/18:2) [2] | Diacylglycerol | 2.52 | 5.7E-03 |
| 1-palmitoleoyl-2-linolenoyl-gpc (16:1/18:3) | Phosphatidylcholine (PC) | 2.38 | 2.4E-03 |
| acetoacetate | Ketone Bodies | 2.35 | 2.1E-02 |
| sphingomyelin (d18:1/24:1, d18:2/24:0) | Sphingomyelins | 2.32 | <3.0E-04 |
| isocitrate | TCA Cycle | 2.31 | <3.0E-04 |
| lyxonate | Pentose Metabolism | 2.31 | 4.2E-02 |
| 1-lignoceroyl-gpc (24:0) | Lysophospholipid | 2.29 | <3.0E-04 |
| cytidine | Pyrimidine Metabolism, Cytidine containing | 2.28 | 3.1E-03 |
| 1-linoleoyl-2-linolenoyl-gpc (18:2/18:3) | Phosphatidylcholine (PC) | 2.11 | 2.8E-03 |
| 1-oleoylglycerol (18:1) | Monoacylglycerol | 2.08 | 5.5E-03 |
| n-acetylglycine | Glycine, Serine and Threonine Metabolism | 2.06 | 1.0E-03 |
| sphinganine-1-phosphate | Sphingolipid Synthesis | 2.06 | 2.2E-03 |
| linoleoyl-arachidonoyl-glycerol (18:2/20:4) [1] | Diacylglycerol | 2.05 | 3.1E-03 |
| 3-aminoisobutyrate | Pyrimidine Metabolism, Thymine containing | 2.05 | 2.6E-03 |
| linoleoyl-arachidonoyl-glycerol (18:2/20:4) [2] | Diacylglycerol | 2.01 | 2.9E-03 |
| 1,2-dilinoleoyl-gpe (18:2/18:2) | Phosphatidylethanolamine (PE) | 2.00 | 2.0E-02 |
| 1-oleoyl-2-linoleoyl-gpe (18:1/18:2) | Phosphatidylethanolamine (PE) | 2.00 | 3.8E-03 |
| oleoyl-arachidonoyl-glycerol (18:1/20:4) [2] | Diacylglycerol | 2.00 | 3.7E-03 |
| 1-stearoyl-2-oleoyl-gps (18:0/18:1) | Phosphatidylserine (PS) | 2.00 | 5.3E-03 |
| 1-stearoyl-2-arachidonoyl-gpe (18:0/20:4) | Phosphatidylethanolamine (PE) | 1.99 | <3.0E-04 |
| 1-palmitoyl-2-oleoyl-gpe (16:0/18:1) | Phosphatidylethanolamine (PE) | 1.99 | 1.8E-03 |
| 1-palmitoyl-2-arachidonoyl-gpe (16:0/20:4) | Phosphatidylethanolamine (PE) | 1.99 | 8.9E-04 |
| oleoyl-linoleoyl-glycerol (18:1/18:2) [1] | Diacylglycerol | 1.97 | 2.1E-03 |
| orotidine | Pyrimidine Metabolism, Orotate containing | 1.97 | 4.4E-02 |
| oleoylcholine | Fatty Acid Metabolism (Acyl Choline) | 1.94 | 2.8E-03 |
| hexanoylglycine | Fatty Acid Metabolism (Acyl Glycine) | 1.91 | 2.5E-02 |
| arachidonoylcholine | Fatty Acid Metabolism (Acyl Choline) | 1.88 | 5.7E-03 |
| ergothioneine | Food Component/Plant | 1.85 | 6.2E-03 |
| gluconate | Food Component/Plant | 1.84 | 1.7E-02 |
| dihomo-linolenoyl-choline | Fatty Acid Metabolism (Acyl Choline) | 1.83 | 1.7E-02 |
| 1,2-dilinoleoyl-gpc (18:2/18:2) | Phosphatidylcholine (PC) | 1.83 | <3.0E-04 |
| 1-stearoyl-2-linoleoyl-gpe (18:0/18:2) | Phosphatidylethanolamine (PE) | 1.83 | 2.8E-03 |
| carotene diol (1) | Vitamin A Metabolism | 1.83 | 5.6E-03 |
| sphingomyelin (d18:2/24:2) | Sphingomyelins | 1.82 | <3.0E-04 |
| glutamine_degradant | Partially Characterized Molecules | 1.82 | 6.9E-03 |
| 1-linoleoyl-2-arachidonoyl-gpc (18:2/20:4n6) | Phosphatidylcholine (PC) | 1.80 | <3.0E-04 |
| stearoylcholine | Fatty Acid Metabolism (Acyl Choline) | 1.80 | 8.6E-03 |
| palmitoyl-sphingosine-phosphoethanolamine (d18:1/16:0) | Ceramide PEs | 1.80 | <3.0E-04 |
| 12,13-dihome | Fatty Acid, Dihydroxy | 1.79 | 6.8E-03 |
| sphingomyelin (d18:2/24:1, d18:1/24:2) | Sphingomyelins | 1.77 | <3.0E-04 |
| octadecadienedioate (c18:2-dc) | Fatty Acid, Dicarboxylate | 1.75 | 1.6E-02 |
| carotene diol (3) | Vitamin A Metabolism | 1.75 | 2.7E-02 |
| choline phosphate | Phospholipid Metabolism | 1.75 | 3.7E-03 |
| lactosyl-n-nervonoyl-sphingosine (d18:1/24:1) | Lactosylceramides (LCER) | 1.74 | 8.0E-04 |
| 9,10-dihome | Fatty Acid, Dihydroxy | 1.73 | 1.5E-02 |
| n-acetyltaurine | Methionine, Cysteine, SAM and Taurine Metabolism | 1.73 | 2.2E-02 |
| glucuronate | Aminosugar Metabolism | 1.71 | 2.7E-02 |
| sphingosine 1-phosphate | Sphingosines | 1.70 | 2.4E-03 |
| oleoyl-arachidonoyl-glycerol (18:1/20:4) [1] | Diacylglycerol | 1.70 | 2.1E-02 |
| 1-arachidonoyl-gpe (20:4n6) | Lysophospholipid | 1.69 | <3.0E-04 |
| 1-palmitoyl-2-linoleoyl-gpe (16:0/18:2) | Phosphatidylethanolamine (PE) | 1.68 | 2.2E-02 |
| carotene diol (2) | Vitamin A Metabolism | 1.68 | 8.6E-03 |
| 1-ribosyl-imidazoleacetate | Histidine Metabolism | 1.67 | 7.6E-04 |
| 1-linolenoyl-gpc (18:3) | Lysophospholipid | 1.65 | 9.3E-03 |
| 1-(1-enyl-palmitoyl)-2-linoleoyl-gpc (p-16:0/18:2) | Plasmalogen | 1.64 | 8.4E-04 |
| 1-stearoyl-2-arachidonoyl-gpc (18:0/20:4) | Phosphatidylcholine (PC) | 1.64 | <3.0E-04 |
| 1-linoleoyl-gpe (18:2) | Lysophospholipid | 1.63 | 2.4E-03 |
| 1-palmitoyl-2-dihomo-linolenoyl-gpc (16:0/20:3n3 or 6) | Phosphatidylcholine (PC) | 1.62 | <3.0E-04 |
| sphingomyelin (d18:2/16:0, d18:1/16:1) | Sphingomyelins | 1.62 | <3.0E-04 |
| 1-arachidonoyl-gpc (20:4n6) | Lysophospholipid | 1.61 | 3.2E-03 |
| 1-(1-enyl-palmitoyl)-2-oleoyl-gpe (p-16:0/18:1) | Plasmalogen | 1.61 | 1.9E-03 |
| retinol (vitamin a) | Vitamin A Metabolism | 1.61 | 1.5E-02 |
| 1-palmitoyl-2-arachidonoyl-gpc (16:0/20:4n6) | Phosphatidylcholine (PC) | 1.58 | 7.3E-04 |
| 1-(1-enyl-palmitoyl)-2-palmitoleoyl-gpc (p-16:0/16:1) | Plasmalogen | 1.58 | 3.2E-03 |
| palmitoylcholine | Fatty Acid Metabolism (Acyl Choline) | 1.58 | 3.6E-02 |
| glycine | Glycine, Serine and Threonine Metabolism | 1.54 | 8.5E-04 |
| 1-(1-enyl-palmitoyl)-2-arachidonoyl-gpc (p-16:0/20:4) | Plasmalogen | 1.54 | 5.7E-03 |
| 1-methyl-4-imidazoleacetate | Histidine Metabolism | 1.54 | 6.6E-03 |
| behenoyl sphingomyelin (d18:1/22:0) | Sphingomyelins | 1.54 | 1.5E-02 |
| 1,2-dipalmitoyl-gpc (16:0/16:0) | Phosphatidylcholine (PC) | 1.53 | 1.8E-03 |
| sphingomyelin (d18:2/18:1) | Sphingomyelins | 1.52 | 1.7E-02 |
| palmitoyl dihydrosphingomyelin (d18:0/16:0) | Dihydrosphingomyelins | 1.52 | 2.1E-03 |
| oxalate (ethanedioate) | Ascorbate and Aldarate Metabolism | 1.50 | 2.8E-03 |
| n2,n2-dimethylguanosine | Purine Metabolism, Guanine containing | 1.49 | 1.6E-03 |
| succinylcarnitine (c4-dc) | TCA Cycle | 1.49 | 3.2E-02 |
| 1-palmitoyl-2-linoleoyl-gpc (16:0/18:2) | Phosphatidylcholine (PC) | 1.47 | 2.9E-03 |
| dodecadienoate (12:2) | Fatty Acid, Dicarboxylate | 1.46 | 4.1E-02 |
| glycosyl ceramide (d18:2/24:1, d18:1/24:2) | Hexosylceramides (HCER) | 1.46 | 6.6E-03 |
| 1-(1-enyl-palmitoyl)-2-oleoyl-gpc (p-16:0/18:1) | Plasmalogen | 1.46 | 2.9E-03 |
| sphingomyelin (d18:1/20:1, d18:2/20:0) | Sphingomyelins | 1.45 | 6.8E-03 |
| 1-stearoyl-2-linoleoyl-gpc (18:0/18:2) | Phosphatidylcholine (PC) | 1.45 | 2.7E-03 |
| 1-palmitoyl-2-stearoyl-gpc (16:0/18:0) | Phosphatidylcholine (PC) | 1.45 | 1.7E-03 |
| palmitoyl sphingomyelin (d18:1/16:0) | Sphingomyelins | 1.45 | 2.5E-03 |
| 3-ureidopropionate | Pyrimidine Metabolism, Uracil containing | 1.44 | 1.6E-02 |
| 1-palmitoyl-2-oleoyl-gpc (16:0/18:1) | Phosphatidylcholine (PC) | 1.44 | 3.1E-03 |
| lignoceroyl sphingomyelin (d18:1/24:0) | Sphingomyelins | 1.43 | 1.5E-02 |
| 1-oleoyl-gpc (18:1) | Lysophospholipid | 1.42 | 6.9E-04 |
| hydroxypalmitoyl sphingomyelin (d18:1/16:0(oh)) | Sphingomyelins | 1.41 | 1.5E-02 |
| gamma-glutamylglycine | Gamma-glutamyl Amino Acid | 1.41 | 6.9E-03 |
| 1-stearoyl-gpe (18:0) | Lysophospholipid | 1.39 | 1.6E-02 |
| adenine | Purine Metabolism, Adenine containing | 1.39 | 1.8E-02 |
| glycerophosphorylcholine (gpc) | Phospholipid Metabolism | 1.38 | 2.7E-02 |
| sphingomyelin (d18:2/21:0, d16:2/23:0) | Sphingomyelins | 1.38 | 3.2E-02 |
| 1-linoleoyl-gpc (18:2) | Lysophospholipid | 1.37 | 5.8E-03 |
| sphingomyelin (d18:1/22:1, d18:2/22:0, d16:1/24:1) | Sphingomyelins | 1.37 | 1.6E-02 |
| isoleucylglycine | Dipeptide | 1.36 | 3.0E-02 |
| 1-stearoyl-2-oleoyl-gpc (18:0/18:1) | Phosphatidylcholine (PC) | 1.35 | 1.6E-02 |
| sphingomyelin (d18:1/18:1, d18:2/18:0) | Sphingomyelins | 1.35 | 2.9E-02 |
| sphingomyelin (d18:2/23:0, d18:1/23:1, d17:1/24:1) | Sphingomyelins | 1.34 | 1.8E-02 |
| 1-palmitoleoyl-gpc (16:1) | Lysophospholipid | 1.33 | 3.7E-02 |
| tricosanoyl sphingomyelin (d18:1/23:0) | Sphingomyelins | 1.33 | 4.3E-02 |
| 5-methylthioadenosine (mta) | Polyamine Metabolism | 1.32 | 1.5E-02 |
| aconitate [cis or trans] | TCA Cycle | 1.32 | 8.6E-03 |
| cholesterol | Sterol | 1.31 | 2.1E-02 |
| 1-palmitoyl-gpc (16:0) | Lysophospholipid | 1.30 | 4.6E-03 |
| 1-stearoyl-gpc (18:0) | Lysophospholipid | 1.28 | 1.6E-02 |
| lactosyl-n-palmitoyl-sphingosine (d18:1/16:0) | Lactosylceramides (LCER) | 1.25 | 3.3E-02 |
| dimethylarginine (sdma + adma) | Urea cycle; Arginine and Proline Metabolism | 1.15 | 3.6E-02 |
| cystine | Methionine, Cysteine, SAM and Taurine Metabolism | 0.82 | 3.5E-02 |
| methionine sulfoxide | Methionine, Cysteine, SAM and Taurine Metabolism | 0.82 | 2.3E-02 |
| phenylalanine | Phenylalanine Metabolism | 0.81 | 1.9E-02 |
| lysine | Lysine Metabolism | 0.81 | 1.9E-02 |
| threonine | Glycine, Serine and Threonine Metabolism | 0.79 | 1.3E-02 |
| cysteine sulfinic acid | Methionine, Cysteine, SAM and Taurine Metabolism | 0.78 | 7.6E-03 |
| n6-acetyllysine | Lysine Metabolism | 0.77 | 1.4E-02 |
| gamma-glutamylvaline | Gamma-glutamyl Amino Acid | 0.77 | 2.3E-02 |
| glutamate | Glutamate Metabolism | 0.77 | 4.0E-02 |
| leucine | Leucine, Isoleucine and Valine Metabolism | 0.76 | 4.9E-03 |
| 3-methyl-2-oxobutyrate | Leucine, Isoleucine and Valine Metabolism | 0.76 | 4.6E-02 |
| gamma-glutamylmethionine | Gamma-glutamyl Amino Acid | 0.76 | 2.9E-02 |
| caproate (6:0) | Medium Chain Fatty Acid | 0.75 | 2.3E-02 |
| trans-4-hydroxyproline | Urea cycle; Arginine and Proline Metabolism | 0.74 | 1.6E-02 |
| isoleucine | Leucine, Isoleucine and Valine Metabolism | 0.74 | 2.8E-03 |
| stearoylcarnitine (c18) | Fatty Acid Metabolism (Acyl Carnitine, Long Chain Saturated) | 0.74 | 3.7E-02 |
| tryptophan | Tryptophan Metabolism | 0.74 | 1.2E-02 |
| 2-aminobutyrate | Glutathione Metabolism | 0.74 | 1.4E-02 |
| n-acetyltyrosine | Tyrosine Metabolism | 0.74 | 4.2E-02 |
| 6-bromotryptophan | Tryptophan Metabolism | 0.74 | 4.4E-02 |
| n-acetylvaline | Leucine, Isoleucine and Valine Metabolism | 0.73 | 1.4E-02 |
| gamma-glutamylleucine | Gamma-glutamyl Amino Acid | 0.73 | 8.9E-03 |
| gamma-glutamylphenylalanine | Gamma-glutamyl Amino Acid | 0.72 | 1.9E-02 |
| ceramide (d18:1/20:0, d16:1/22:0, d20:1/18:0) | Ceramides | 0.72 | 3.5E-02 |
| 3-(4-hydroxyphenyl)lactate | Tyrosine Metabolism | 0.72 | 2.0E-02 |
| 2-hydroxy-3-methylvalerate | Leucine, Isoleucine and Valine Metabolism | 0.72 | 3.6E-02 |
| methylsuccinate | Leucine, Isoleucine and Valine Metabolism | 0.72 | 3.9E-02 |
| uridine | Pyrimidine Metabolism, Uracil containing | 0.70 | 1.9E-02 |
| cysteine s-sulfate | Methionine, Cysteine, SAM and Taurine Metabolism | 0.70 | 3.9E-02 |
| valine | Leucine, Isoleucine and Valine Metabolism | 0.70 | 4.9E-03 |
| 1-stearoyl-2-docosahexaenoyl-gpc (18:0/22:6) | Phosphatidylcholine (PC) | 0.69 | 1.9E-02 |
| formiminoglutamate | Histidine Metabolism | 0.69 | 3.3E-02 |
| lignoceroylcarnitine (c24) | Fatty Acid Metabolism (Acyl Carnitine, Long Chain Saturated) | 0.69 | 4.7E-02 |
| 3-hydroxy-2-ethylpropionate | Leucine, Isoleucine and Valine Metabolism | 0.68 | 3.3E-02 |
| 2r,3r-dihydroxybutyrate | Fatty Acid, Dihydroxy | 0.68 | 3.4E-02 |
| gamma-glutamylisoleucine | Gamma-glutamyl Amino Acid | 0.67 | 1.0E-02 |
| palmitate (16:0) | Long Chain Saturated Fatty Acid | 0.66 | 2.8E-02 |
| n-formylanthranilic acid | Tryptophan Metabolism | 0.66 | 4.5E-02 |
| n-stearoyltaurine | Endocannabinoid | 0.66 | 3.0E-02 |
| margarate (17:0) | Long Chain Saturated Fatty Acid | 0.66 | 4.6E-02 |
| n-stearoyl-sphingadienine (d18:2/18:0) | Ceramides | 0.66 | 2.6E-02 |
| linoleoylcarnitine (c18:2) | Fatty Acid Metabolism (Acyl Carnitine, Polyunsaturated) | 0.66 | 4.9E-02 |
| 5-methyluridine (ribothymidine) | Pyrimidine Metabolism, Uracil containing | 0.66 | 3.7E-02 |
| pentadecanoate (15:0) | Long Chain Saturated Fatty Acid | 0.66 | 1.9E-02 |
| cysteine | Methionine, Cysteine, SAM and Taurine Metabolism | 0.65 | 9.3E-03 |
| oleoylcarnitine (c18:1) | Fatty Acid Metabolism (Acyl Carnitine, Monounsaturated) | 0.65 | 2.4E-02 |
| palmitoleoylcarnitine (c16:1) | Fatty Acid Metabolism (Acyl Carnitine, Monounsaturated) | 0.65 | 2.4E-02 |
| proline | Urea cycle; Arginine and Proline Metabolism | 0.65 | 1.1E-03 |
| 1-methylhistidine | Histidine Metabolism | 0.65 | 1.3E-02 |
| beta-hydroxyisovalerate | Leucine, Isoleucine and Valine Metabolism | 0.64 | 2.2E-02 |
| 3-methoxytyramine sulfate | Tyrosine Metabolism | 0.64 | 1.6E-02 |
| octadecanedioylcarnitine (c18-dc) | Fatty Acid Metabolism (Acyl Carnitine, Dicarboxylate) | 0.63 | 4.9E-02 |
| lactate | Glycolysis, Gluconeogenesis, and Pyruvate Metabolism | 0.63 | 1.4E-02 |
| sphingosine | Sphingosines | 0.63 | 2.6E-02 |
| behenoylcarnitine (c22) | Fatty Acid Metabolism (Acyl Carnitine, Long Chain Saturated) | 0.63 | 3.3E-02 |
| indoleacetate | Tryptophan Metabolism | 0.62 | 2.3E-02 |
| stearate (18:0) | Long Chain Saturated Fatty Acid | 0.62 | 8.5E-03 |
| sphinganine | Sphingolipid Synthesis | 0.62 | 1.1E-02 |
| gamma-glutamylthreonine | Gamma-glutamyl Amino Acid | 0.60 | 1.0E-03 |
| thyroxine | Tyrosine Metabolism | 0.59 | 6.7E-04 |
| tiglylcarnitine (c5:1-dc) | Leucine, Isoleucine and Valine Metabolism | 0.59 | 8.5E-03 |
| picolinoylglycine | Fatty Acid Metabolism (Acyl Glycine) | 0.59 | 1.6E-02 |
| alpha-ketoglutarate | TCA Cycle | 0.59 | 1.4E-02 |
| gamma-glutamyltryptophan | Gamma-glutamyl Amino Acid | 0.59 | 4.3E-03 |
| laurylcarnitine (c12) | Fatty Acid Metabolism (Acyl Carnitine, Medium Chain) | 0.59 | 2.0E-02 |
| 8-methoxykynurenate | Tryptophan Metabolism | 0.59 | 2.2E-02 |
| nonanoylcarnitine (c9) | Fatty Acid Metabolism (Acyl Carnitine, Medium Chain) | 0.58 | 1.6E-02 |
| guanidinoacetate | Creatine Metabolism | 0.58 | 1.4E-02 |
| 21-hydroxypregnenolone disulfate | Pregnenolone Steroids | 0.57 | 3.0E-02 |
| sphingomyelin (d18:1/25:0, d19:0/24:1, d20:1/23:0, d19:1/24:0) | Sphingomyelins | 0.57 | 8.2E-03 |
| isovalerylglycine | Leucine, Isoleucine and Valine Metabolism | 0.57 | 2.3E-02 |
| 3-hydroxystearate | Fatty Acid, Monohydroxy | 0.57 | 5.4E-03 |
| s-adenosylhomocysteine (sah) | Methionine, Cysteine, SAM and Taurine Metabolism | 0.56 | 5.2E-03 |
| gamma-glutamyltyrosine | Gamma-glutamyl Amino Acid | 0.55 | 8.5E-04 |
| 3-indoxyl sulfate | Tryptophan Metabolism | 0.55 | 1.4E-02 |
| butyrate/isobutyrate (4:0) | Short Chain Fatty Acid | 0.54 | 4.0E-03 |
| isovalerylcarnitine (c5) | Leucine, Isoleucine and Valine Metabolism | 0.54 | 5.4E-03 |
| taurocholenate sulfate | Secondary Bile Acid Metabolism | 0.53 | 1.6E-02 |
| myristoylcarnitine (c14) | Fatty Acid Metabolism (Acyl Carnitine, Long Chain Saturated) | 0.53 | 7.6E-04 |
| palmitoylcarnitine (c16) | Fatty Acid Metabolism (Acyl Carnitine, Long Chain Saturated) | 0.53 | 5.3E-04 |
| (16 or 17)-methylstearate (a19:0 or i19:0) | Fatty Acid, Branched | 0.53 | 4.9E-03 |
| 2-methylbutyrylcarnitine (c5) | Leucine, Isoleucine and Valine Metabolism | 0.53 | 2.0E-03 |
| branched-chain, straight-chain, or cyclopropyl 10:1 fatty acid (1) | Partially Characterized Molecules | 0.52 | 3.9E-03 |
| propionylcarnitine (c3) | Fatty Acid Metabolism (also BCAA Metabolism) | 0.52 | 4.2E-03 |
| gamma-cehc glucuronide | Tocopherol Metabolism | 0.51 | 2.5E-02 |
| 2-linoleoylglycerol (18:2) | Monoacylglycerol | 0.51 | 2.3E-02 |
| delta-cehc | Tocopherol Metabolism | 0.51 | 1.9E-02 |
| gamma-glutamylglutamate | Gamma-glutamyl Amino Acid | 0.50 | 4.3E-03 |
| n-palmitoyl-heptadecasphingosine (d17:1/16:0) | Ceramides | 0.50 | 4.8E-04 |
| eicosapentaenoate (epa; 20:5n3) | Long Chain Polyunsaturated Fatty Acid (n3 and n6) | 0.50 | 1.0E-02 |
| indolin-2-one | Food Component/Plant | 0.50 | 1.7E-02 |
| 10-undecenoate (11:1n1) | Medium Chain Fatty Acid | 0.49 | 6.7E-04 |
| 3-carboxy-4-methyl-5-pentyl-2-furanpropionate (3-cmpfp) | Fatty Acid, Dicarboxylate | 0.49 | 6.3E-04 |
| 5-hepe | Eicosanoid | 0.48 | 1.3E-02 |
| docosapentaenoylcarnitine (c22:5n3) | Fatty Acid Metabolism (Acyl Carnitine, Polyunsaturated) | 0.47 | 9.7E-03 |
| erythritol | Food Component/Plant | 0.47 | 3.1E-02 |
| n-formylphenylalanine | Tyrosine Metabolism | 0.47 | 5.7E-03 |
| delta-cehc glucuronide | Tocopherol Metabolism | 0.47 | 2.1E-02 |
| (2,4 or 2,5)-dimethylphenol sulfate | Food Component/Plant | 0.47 | 4.6E-02 |
| ceramide (d16:1/24:1, d18:1/22:1) | Ceramides | 0.47 | 9.2E-03 |
| cortolone glucuronide (1) | Corticosteroids | 0.47 | 3.3E-03 |
| ceramide (d18:1/17:0, d17:1/18:0) | Ceramides | 0.47 | 1.1E-03 |
| 1-palmitoylglycerol (16:0) | Monoacylglycerol | 0.45 | 6.2E-03 |
| 1-linoleoylglycerol (18:2) | Monoacylglycerol | 0.45 | 2.3E-02 |
| pyruvate | Glycolysis, Gluconeogenesis, and Pyruvate Metabolism | 0.45 | 4.1E-03 |
| decanoylcarnitine (c10) | Fatty Acid Metabolism (Acyl Carnitine, Medium Chain) | 0.45 | 1.5E-02 |
| 1-arachidonylglycerol (20:4) | Monoacylglycerol | 0.45 | 4.6E-02 |
| 16a-hydroxy dhea 3-sulfate | Androgenic Steroids | 0.45 | 1.0E-02 |
| phenylacetate | Phenylalanine Metabolism | 0.45 | 3.4E-02 |
| indoleacetylglutamine | Tryptophan Metabolism | 0.43 | 1.8E-02 |
| 1-myristoylglycerol (14:0) | Monoacylglycerol | 0.42 | 4.1E-03 |
| andro steroid monosulfate c19h28o6s (1) | Androgenic Steroids | 0.42 | 8.9E-03 |
| undecanedioate (c11-dc) | Fatty Acid, Dicarboxylate | 0.42 | 3.0E-02 |
| 1-dihomo-linolenylglycerol (20:3) | Monoacylglycerol | 0.41 | 2.7E-02 |
| isoursodeoxycholate | Secondary Bile Acid Metabolism | 0.40 | 3.5E-02 |
| 2'-o-methyluridine | Pyrimidine Metabolism, Uracil containing | 0.40 | 4.3E-03 |
| 4-hydroxyphenylpyruvate | Tyrosine Metabolism | 0.39 | 6.9E-04 |
| 2-hydroxybutyrate/2-hydroxyisobutyrate | Glutathione Metabolism | 0.39 | 3.3E-03 |
| 14-hdohe/17-hdohe | Docosanoid | 0.39 | 1.3E-02 |
| 3,5-dichloro-2,6-dihydroxybenzoic acid | Chemical | 0.39 | 3.8E-04 |
| 1-arachidonoyl-gpa (20:4) | Lysophospholipid | 0.39 | 2.7E-02 |
| n,n,n-trimethyl-5-aminovalerate | Lysine Metabolism | 0.39 | 5.3E-04 |
| tyramine o-sulfate | Tyrosine Metabolism | 0.38 | 2.3E-02 |
| heme | Hemoglobin and Porphyrin Metabolism | 0.37 | 1.9E-02 |
| sulfate of piperine metabolite c16h19no3 (2) | Food Component/Plant | 0.37 | 1.6E-02 |
| glycochenodeoxycholate glucuronide (1) | Primary Bile Acid Metabolism | 0.36 | 1.6E-02 |
| maleate | Fatty Acid, Dicarboxylate | 0.36 | 4.9E-03 |
| heptenedioate (c7:1-dc) | Fatty Acid, Dicarboxylate | 0.36 | 5.6E-04 |
| margaroylcarnitine (c17) | Fatty Acid Metabolism (Acyl Carnitine, Long Chain Saturated) | 0.35 | 1.8E-03 |
| 1-linoleoyl-gpa (18:2) | Lysophospholipid | 0.35 | 4.9E-03 |
| 3-methylhistidine | Histidine Metabolism | 0.35 | 7.9E-03 |
| glycoursodeoxycholic acid sulfate (1) | Secondary Bile Acid Metabolism | 0.34 | 3.1E-02 |
| glucuronide of piperine metabolite c17h21no3 (5) | Food Component/Plant | 0.33 | 1.6E-02 |
| p-cresol glucuronide | Tyrosine Metabolism | 0.33 | 4.2E-02 |
| azelate (c9-dc) | Fatty Acid, Dicarboxylate | 0.33 | 3.3E-02 |
| ursodeoxycholate | Secondary Bile Acid Metabolism | 0.33 | 3.8E-02 |
| 1-pentadecanoylglycerol (15:0) | Monoacylglycerol | 0.33 | 3.3E-03 |
| 2-palmitoylglycerol (16:0) | Monoacylglycerol | 0.32 | 6.8E-03 |
| fructose | Fructose, Mannose and Galactose Metabolism | 0.31 | 7.3E-04 |
| tridecenedioate (c13:1-dc) | Fatty Acid, Dicarboxylate | 0.30 | 5.9E-04 |
| undecenoylcarnitine (c11:1) | Fatty Acid Metabolism (Acyl Carnitine, Monounsaturated) | 0.29 | 5.9E-04 |
| perfluorooctanesulfonate (pfos) | Chemical | 0.29 | 4.1E-04 |
| docosahexaenoate (dha; 22:6n3) | Long Chain Polyunsaturated Fatty Acid (n3 and n6) | 0.29 | 1.1E-03 |
| glucuronide of piperine metabolite c17h21no3 (3) | Food Component/Plant | 0.29 | 8.4E-03 |
| sulfate of piperine metabolite c18h21no3 (1) | Food Component/Plant | 0.28 | 1.1E-02 |
| 3-bromo-5-chloro-2,6-dihydroxybenzoic acid | Chemical | 0.28 | 7.6E-04 |
| (14 or 15)-methylpalmitate (a17:0 or i17:0) | Fatty Acid, Branched | 0.28 | 8.9E-04 |
| phenylacetylcarnitine | Acetylated Peptides | 0.27 | 1.5E-03 |
| 1-palmitoyl-gpa (16:0) | Lysophospholipid | 0.26 | 5.6E-03 |
| sulfate of piperine metabolite c18h21no3 (3) | Food Component/Plant | 0.26 | 3.9E-03 |
| sulfate of piperine metabolite c16h19no3 (3) | Food Component/Plant | 0.25 | 3.9E-03 |
| 3-methyladipate | Fatty Acid, Dicarboxylate | 0.23 | 4.4E-04 |
| 1-margaroylglycerol (17:0) | Monoacylglycerol | 0.22 | 9.9E-04 |
| 1-docosahexaenoylglycerol (22:6) | Monoacylglycerol | 0.22 | 6.7E-04 |
| glucuronide of piperine metabolite c17h21no3 (4) | Food Component/Plant | 0.20 | 2.6E-03 |
| glutamine conjugate of c7h12o2 | Partially Characterized Molecules | 0.17 | 7.1E-04 |
| docosahexaenoylcarnitine (c22:6) | Fatty Acid Metabolism (Acyl Carnitine, Polyunsaturated) | 0.15 | 1.3E-03 |
| piperine | Food Component/Plant | 0.12 | 1.4E-03 |
| hydroxy-cmpf | Fatty Acid, Dicarboxylate | 0.01 | 5.3E-03 |
| 3-carboxy-4-methyl-5-propyl-2-furanpropanoate (cmpf) | Fatty Acid, Dicarboxylate | 0.01 | 2.7E-03 |

^1^Adjustment for race, BMI, sex, age, batch, study, and batch*study

| **Supplementary Table 13.** Plasma metabolites associated with both plasma creatinine and vegan relative to pesco-vegetarian dietary pattern at FDR < 0.05^1,2^ | | | | | |
| --- | --- | --- | --- | --- | --- |
|  |  | **Creatinine** | | **Vegan** | |
| **Metabolite** | **Subclass** | **Fold Change** | **FDR** | **Fold Change** | **FDR** |
| piperine | Food Component/Plant | 5.10 | 8.4E-03 | 0.12 | 1.4E-03 |
| glucuronide of piperine metabolite c17h21no3 (4) | Food Component/Plant | 4.02 | 2.1E-03 | 0.20 | 2.6E-03 |
| 3-methylhistidine | Histidine Metabolism | 3.96 | 3.2E-03 | 0.35 | 7.9E-03 |
| 3-carboxy-4-methyl-5-propyl-2-furanpropanoate (cmpf) | Fatty Acid, Dicarboxylate | 3.72 | 2.8E-02 | 0.01 | 2.7E-03 |
| glucuronide of piperine metabolite c17h21no3 (3) | Food Component/Plant | 3.48 | 2.2E-03 | 0.29 | 8.4E-03 |
| glucuronide of piperine metabolite c17h21no3 (5) | Food Component/Plant | 3.39 | 2.0E-03 | 0.33 | 1.6E-02 |
| sulfate of piperine metabolite c16h19no3 (3) | Food Component/Plant | 3.30 | 3.8E-03 | 0.25 | 3.9E-03 |
| sulfate of piperine metabolite c18h21no3 (1) | Food Component/Plant | 3.08 | 8.6E-03 | 0.28 | 1.1E-02 |
| sulfate of piperine metabolite c16h19no3 (2) | Food Component/Plant | 3.04 | 2.7E-03 | 0.37 | 1.6E-02 |
| ectoine | Chemical | 2.79 | 9.8E-04 | 2.76 | 6.2E-03 |
| p-cresol glucuronide | Tyrosine Metabolism | 2.74 | 4.2E-02 | 0.33 | 4.2E-02 |
| sulfate of piperine metabolite c18h21no3 (3) | Food Component/Plant | 2.72 | 9.2E-03 | 0.26 | 3.9E-03 |
| glutamine conjugate of c7h12o2 | Partially Characterized Molecules | 2.71 | 8.9E-03 | 0.17 | 7.1E-04 |
| (2,4 or 2,5)-dimethylphenol sulfate | Food Component/Plant | 2.42 | 4.2E-03 | 0.47 | 4.6E-02 |
| indoleacetylglutamine | Tryptophan Metabolism | 2.33 | 1.5E-03 | 0.43 | 1.8E-02 |
| tyramine o-sulfate | Tyrosine Metabolism | 2.28 | 4.5E-03 | 0.38 | 2.3E-02 |
| heptenedioate (c7:1-dc) | Fatty Acid, Dicarboxylate | 2.22 | 1.3E-03 | 0.36 | 5.6E-04 |
| n-acetyltaurine | Methionine, Cysteine, SAM and Taurine Metabolism | 1.97 | <3.6E-04 | 1.73 | 2.2E-02 |
| tridecenedioate (c13:1-dc) | Fatty Acid, Dicarboxylate | 1.95 | 6.8E-03 | 0.30 | 5.9E-04 |
| 1-methylhistidine | Histidine Metabolism | 1.83 | <3.6E-04 | 0.65 | 1.3E-02 |
| n,n,n-trimethyl-5-aminovalerate | Lysine Metabolism | 1.75 | 6.1E-03 | 0.39 | 5.3E-04 |
| 8-methoxykynurenate | Tryptophan Metabolism | 1.75 | 1.4E-03 | 0.59 | 2.2E-02 |
| delta-cehc glucuronide | Tocopherol Metabolism | 1.72 | 5.0E-03 | 0.47 | 2.1E-02 |
| gulonate | Ascorbate and Aldarate Metabolism | 1.70 | <3.6E-04 | 2.60 | 1.9E-02 |
| undecenoylcarnitine (c11:1) | Fatty Acid Metabolism (Acyl Carnitine, Monounsaturated) | 1.68 | 1.4E-03 | 0.29 | 5.9E-04 |
| tiglylcarnitine (c5:1-dc) | Leucine, Isoleucine and Valine Metabolism | 1.66 | 6.2E-04 | 0.59 | 8.5E-03 |
| picolinoylglycine | Fatty Acid Metabolism (Acyl Glycine) | 1.61 | 9.4E-04 | 0.59 | 1.6E-02 |
| 2-methylbutyrylcarnitine (c5) | Leucine, Isoleucine and Valine Metabolism | 1.55 | 1.7E-03 | 0.53 | 2.0E-03 |
| 3-carboxy-4-methyl-5-pentyl-2-furanpropionate (3-cmpfp) | Fatty Acid, Dicarboxylate | 1.55 | 1.6E-03 | 0.49 | 6.3E-04 |
| gamma-cehc glucuronide | Tocopherol Metabolism | 1.54 | 8.7E-03 | 0.51 | 2.5E-02 |
| lyxonate | Pentose Metabolism | 1.51 | 1.7E-03 | 2.31 | 4.2E-02 |
| 2r,3r-dihydroxybutyrate | Fatty Acid, Dihydroxy | 1.50 | 5.8E-04 | 0.68 | 3.4E-02 |
| retinol (vitamin a) | Vitamin A Metabolism | 1.48 | <3.6E-04 | 1.61 | 1.5E-02 |
| isovalerylglycine | Leucine, Isoleucine and Valine Metabolism | 1.48 | 5.0E-03 | 0.57 | 2.3E-02 |
| orotidine | Pyrimidine Metabolism, Orotate containing | 1.48 | 4.1E-04 | 1.97 | 4.4E-02 |
| 3-aminoisobutyrate | Pyrimidine Metabolism, Thymine containing | 1.47 | 1.2E-02 | 2.05 | 2.6E-03 |
| indoleacetate | Tryptophan Metabolism | 1.47 | 7.6E-03 | 0.62 | 2.3E-02 |
| pentose acid | Partially Characterized Molecules | 1.47 | 4.9E-02 | 3.58 | 6.3E-04 |
| 3-indoxyl sulfate | Tryptophan Metabolism | 1.45 | 1.1E-02 | 0.55 | 1.4E-02 |
| erythritol | Food Component/Plant | 1.45 | 1.3E-03 | 0.47 | 3.1E-02 |
| ceramide (d18:1/17:0, d17:1/18:0) | Ceramides | 1.45 | 1.3E-02 | 0.47 | 1.1E-03 |
| margaroylcarnitine (c17) | Fatty Acid Metabolism (Acyl Carnitine, Long Chain Saturated) | 1.43 | 6.5E-03 | 0.35 | 1.8E-03 |
| glucuronate | Aminosugar Metabolism | 1.40 | 4.6E-03 | 1.71 | 2.7E-02 |
| s-adenosylhomocysteine (sah) | Methionine, Cysteine, SAM and Taurine Metabolism | 1.39 | 4.9E-04 | 0.56 | 5.2E-03 |
| beta-hydroxyisovalerate | Leucine, Isoleucine and Valine Metabolism | 1.39 | 6.1E-03 | 0.64 | 2.2E-02 |
| 1-palmitoyl-2-linoleoyl-gpe (16:0/18:2) | Phosphatidylethanolamine (PE) | 1.38 | 1.1E-02 | 1.68 | 2.2E-02 |
| taurocholenate sulfate | Secondary Bile Acid Metabolism | 1.38 | 4.1E-02 | 0.53 | 1.6E-02 |
| sphingomyelin (d18:1/25:0, d19:0/24:1, d20:1/23:0, d19:1/24:0) | Sphingomyelins | 1.36 | 4.9E-02 | 0.57 | 8.2E-03 |
| 3-hydroxy-2-ethylpropionate | Leucine, Isoleucine and Valine Metabolism | 1.36 | 1.0E-02 | 0.68 | 3.3E-02 |
| n-formylanthranilic acid | Tryptophan Metabolism | 1.36 | 2.6E-02 | 0.66 | 4.5E-02 |
| n-palmitoyl-heptadecasphingosine (d17:1/16:0) | Ceramides | 1.35 | 8.3E-03 | 0.50 | 4.8E-04 |
| glutamine_degradant | Partially Characterized Molecules | 1.35 | 3.2E-02 | 1.82 | 6.9E-03 |
| 1-oleoyl-2-linoleoyl-gpe (18:1/18:2) | Phosphatidylethanolamine (PE) | 1.34 | 2.3E-02 | 2.00 | 3.8E-03 |
| decanoylcarnitine (c10) | Fatty Acid Metabolism (Acyl Carnitine, Medium Chain) | 1.33 | 4.4E-02 | 0.45 | 1.5E-02 |
| formiminoglutamate | Histidine Metabolism | 1.32 | 1.6E-02 | 0.69 | 3.3E-02 |
| n-stearoyl-sphingadienine (d18:2/18:0) | Ceramides | 1.32 | 1.3E-02 | 0.66 | 2.6E-02 |
| maleate | Fatty Acid, Dicarboxylate | 1.31 | 4.1E-02 | 0.36 | 4.9E-03 |
| 1-ribosyl-imidazoleacetate | Histidine Metabolism | 1.31 | 4.6E-03 | 1.67 | 7.6E-04 |
| laurylcarnitine (c12) | Fatty Acid Metabolism (Acyl Carnitine, Medium Chain) | 1.31 | 3.9E-02 | 0.59 | 2.0E-02 |
| gamma-glutamylisoleucine | Gamma-glutamyl Amino Acid | 1.31 | 4.2E-03 | 0.67 | 1.0E-02 |
| trans-4-hydroxyproline | Urea cycle; Arginine and Proline Metabolism | 1.30 | 7.0E-03 | 0.74 | 1.6E-02 |
| n-stearoyltaurine | Endocannabinoid | 1.29 | 3.8E-02 | 0.66 | 3.0E-02 |
| choline phosphate | Phospholipid Metabolism | 1.29 | 1.5E-02 | 1.75 | 3.7E-03 |
| cystine | Methionine, Cysteine, SAM and Taurine Metabolism | 1.28 | 4.7E-04 | 0.82 | 3.5E-02 |
| 5-methylthioadenosine (mta) | Polyamine Metabolism | 1.27 | 1.5E-03 | 1.32 | 1.5E-02 |
| succinylcarnitine (c4-dc) | TCA Cycle | 1.27 | 2.8E-02 | 1.49 | 3.2E-02 |
| n-acetyltyrosine | Tyrosine Metabolism | 1.26 | 6.6E-03 | 0.74 | 4.2E-02 |
| 1-(1-enyl-palmitoyl)-2-linoleoyl-gpc (p-16:0/18:2) | Plasmalogen | 1.26 | 1.6E-03 | 1.64 | 8.4E-04 |
| 1-methyl-4-imidazoleacetate | Histidine Metabolism | 1.26 | 1.6E-02 | 1.54 | 6.6E-03 |
| gluconate | Food Component/Plant | 1.26 | 4.8E-03 | 1.84 | 1.7E-02 |
| n2,n2-dimethylguanosine | Purine Metabolism, Guanine containing | 1.25 | 4.3E-04 | 1.49 | 1.6E-03 |
| n6-acetyllysine | Lysine Metabolism | 1.25 | 3.6E-04 | 0.77 | 1.4E-02 |
| sphingomyelin (d18:2/23:0, d18:1/23:1, d17:1/24:1) | Sphingomyelins | 1.25 | 1.5E-03 | 1.34 | 1.8E-02 |
| dimethylarginine (sdma + adma) | Urea cycle; Arginine and Proline Metabolism | 1.24 | <3.6E-04 | 1.15 | 3.6E-02 |
| sphingomyelin (d18:2/18:1) | Sphingomyelins | 1.24 | 1.2E-02 | 1.52 | 1.7E-02 |
| sphingomyelin (d18:2/21:0, d16:2/23:0) | Sphingomyelins | 1.24 | 4.9E-03 | 1.38 | 3.2E-02 |
| myristoylcarnitine (c14) | Fatty Acid Metabolism (Acyl Carnitine, Long Chain Saturated) | 1.22 | 4.3E-02 | 0.53 | 7.6E-04 |
| 3-methoxytyramine sulfate | Tyrosine Metabolism | 1.22 | 2.4E-02 | 0.64 | 1.6E-02 |
| ceramide (d18:1/20:0, d16:1/22:0, d20:1/18:0) | Ceramides | 1.21 | 4.9E-02 | 0.72 | 3.5E-02 |
| methylsuccinate | Leucine, Isoleucine and Valine Metabolism | 1.21 | 2.8E-02 | 0.72 | 3.9E-02 |
| n-acetylvaline | Leucine, Isoleucine and Valine Metabolism | 1.21 | 2.6E-03 | 0.73 | 1.4E-02 |
| stearoylcarnitine (c18) | Fatty Acid Metabolism (Acyl Carnitine, Long Chain Saturated) | 1.20 | 4.0E-02 | 0.74 | 3.7E-02 |
| 1-linoleoyl-gpe (18:2) | Lysophospholipid | 1.20 | 3.5E-02 | 1.63 | 2.4E-03 |
| gamma-glutamylphenylalanine | Gamma-glutamyl Amino Acid | 1.20 | 4.0E-03 | 0.72 | 1.9E-02 |
| cholesterol | Sterol | 1.19 | 6.9E-03 | 1.31 | 2.1E-02 |
| 1-linoleoyl-gpa (18:2) | Lysophospholipid | 1.19 | 3.3E-02 | 0.35 | 4.9E-03 |
| 1-(1-enyl-palmitoyl)-2-arachidonoyl-gpc (p-16:0/20:4) | Plasmalogen | 1.18 | 3.8E-02 | 1.54 | 5.7E-03 |
| sphingomyelin (d18:2/16:0, d18:1/16:1) | Sphingomyelins | 1.18 | 1.3E-03 | 1.62 | <5.3E-04 |
| tricosanoyl sphingomyelin (d18:1/23:0) | Sphingomyelins | 1.18 | 1.5E-02 | 1.33 | 4.3E-02 |
| 1-(1-enyl-palmitoyl)-2-oleoyl-gpc (p-16:0/18:1) | Plasmalogen | 1.16 | 1.1E-02 | 1.46 | 2.9E-03 |
| lignoceroyl sphingomyelin (d18:1/24:0) | Sphingomyelins | 1.16 | 2.6E-02 | 1.43 | 1.5E-02 |
| 1-stearoyl-gpe (18:0) | Lysophospholipid | 1.16 | 3.4E-02 | 1.39 | 1.6E-02 |
| hydroxypalmitoyl sphingomyelin (d18:1/16:0(oh)) | Sphingomyelins | 1.16 | 1.5E-02 | 1.41 | 1.5E-02 |
| sphingomyelin (d18:1/22:1, d18:2/22:0, d16:1/24:1) | Sphingomyelins | 1.16 | 1.7E-03 | 1.37 | 1.6E-02 |
| 1-(1-enyl-palmitoyl)-2-oleoyl-gpe (p-16:0/18:1) | Plasmalogen | 1.16 | 3.7E-02 | 1.61 | 1.9E-03 |
| gamma-glutamylvaline | Gamma-glutamyl Amino Acid | 1.16 | 1.3E-02 | 0.77 | 2.3E-02 |
| sphingomyelin (d18:1/18:1, d18:2/18:0) | Sphingomyelins | 1.14 | 1.3E-02 | 1.35 | 2.9E-02 |
| 1-linoleoyl-gpc (18:2) | Lysophospholipid | 1.13 | 2.2E-02 | 1.37 | 5.8E-03 |
| methionine sulfoxide | Methionine, Cysteine, SAM and Taurine Metabolism | 1.13 | 1.8E-02 | 0.82 | 2.3E-02 |
| behenoyl sphingomyelin (d18:1/22:0) | Sphingomyelins | 1.12 | 2.4E-02 | 1.54 | 1.5E-02 |
| palmitoyl-sphingosine-phosphoethanolamine (d18:1/16:0) | Ceramide PEs | 1.12 | 1.8E-02 | 1.80 | <5.3E-04 |
| sphingomyelin (d18:1/20:1, d18:2/20:0) | Sphingomyelins | 1.10 | 3.7E-02 | 1.45 | 6.8E-03 |
| palmitoyl sphingomyelin (d18:1/16:0) | Sphingomyelins | 1.10 | 2.4E-03 | 1.45 | 2.5E-03 |
| sphingomyelin (d18:1/24:1, d18:2/24:0) | Sphingomyelins | 1.09 | 3.5E-02 | 2.32 | <5.3E-04 |
| cysteine sulfinic acid | Methionine, Cysteine, SAM and Taurine Metabolism | 1.08 | 4.7E-02 | 0.78 | 7.6E-03 |
| sphingomyelin (d18:2/24:1, d18:1/24:2) | Sphingomyelins | 1.08 | 4.7E-02 | 1.77 | <5.3E-04 |
| 1-palmitoyl-2-linoleoyl-gpc (16:0/18:2) | Phosphatidylcholine (PC) | 1.07 | 1.6E-02 | 1.47 | 2.9E-03 |
| ^1^Fold change represents ratio of geometric means of vegan relative to pesco-vegetarian dietary patterns or 90th vs 10th percentiles of creatinine | | | |  |  |
| ^2^Adjustment for race, BMI, sex, age, batch, study, and batch*study | | | | | |

| **Supplementary Table 14.** Metabolites in common among analyses comparing vegan and pesco-vegetarian dietary patterns with non-vegetarian dietary pattern, as well as serum creatinine concentration (high vs low) | | | |
| --- | --- | --- | --- |
| **Metabolite** | **Vegan** | **Pesco-** | **Creatinine** |
| 1-methyl-5-imidazoleacetate | 0.04 | 0.06 | 4.29 |
| 3-methylhistidine | 0.02 | 0.06 | 3.96 |
| 3-carboxy-4-methyl-5-propyl-2-furanpropanoate (cmpf) | 0.04 | 4.54 | 3.72 |
| n-acetyl-1-methylhistidine | 0.31 | 0.3 | 3.35 |
| quinate | 0.28 | 0.13 | 3.18 |
| sulfate of piperine metabolite c16h19no3 (2) | 0.15 | 0.42 | 3.04 |
| homocitrulline | 0.68 | 0.67 | 2.23 |
| phenylacetylglutamate | 0.5 | 0.49 | 2.18 |
| hydantoin-5-propionate | 0.52 | 0.32 | 1.94 |
| p-cresol sulfate | 0.38 | 0.48 | 1.84 |
| 2-butenoylglycine | 3.41 | 2.42 | 1.8 |
| urea | 0.72 | 0.68 | 1.77 |
| n,n,n-trimethyl-5-aminovalerate | 0.16 | 0.43 | 1.75 |
| xanthurenate | 0.63 | 0.55 | 1.7 |
| guaiacol sulfate | 1.95 | 2 | 1.65 |
| beta-hydroxyisovaleroylcarnitine | 0.53 | 0.67 | 1.62 |
| isobutyrylcarnitine (c4) | 0.48 | 0.65 | 1.6 |
| decadienedioic acid (c10:2-dc) | 0.58 | 0.52 | 1.57 |
| 2,3-dihydroxy-2-methylbutyrate | 1.9 | 1.63 | 1.54 |
| quinolinate | 0.71 | 0.71 | 1.53 |
| ceramide (d18:1/17:0, d17:1/18:0) | 0.28 | 0.6 | 1.45 |
| 1-(1-enyl-stearoyl)-2-linoleoyl-gpe (p-18:0/18:2) | 0.56 | 0.53 | 1.44 |
| n-delta-acetylornithine | 2.5 | 1.73 | 1.44 |
| 3-hydroxybutyroylglycine | 2.11 | 1.57 | 1.43 |
| 1-(1-enyl-stearoyl)-2-oleoyl-gpe (p-18:0/18:1) | 0.47 | 0.41 | 1.4 |
| sphingomyelin (d17:2/16:0, d18:2/15:0) | 0.51 | 0.5 | 1.4 |
| sphingomyelin (d18:1/25:0, d19:0/24:1, d20:1/23:0, d19:1/24:0) | 0.23 | 0.42 | 1.36 |
| 1-(1-enyl-palmitoyl)-2-linoleoyl-gpe (p-16:0/18:2) | 0.53 | 0.46 | 1.35 |
| sphingomyelin (d17:1/14:0, d16:1/15:0) | 0.47 | 0.57 | 1.35 |
| 1-(1-enyl-stearoyl)-2-arachidonoyl-gpe (p-18:0/20:4) | 0.37 | 0.37 | 1.34 |
| 1-(1-enyl-stearoyl)-gpe (p-18:0) | 0.46 | 0.49 | 1.33 |
| trans-4-hydroxyproline | 0.45 | 0.61 | 1.3 |
| 1-(1-enyl-palmitoyl)-2-arachidonoyl-gpe (p-16:0/20:4) | 0.42 | 0.4 | 1.27 |
| sphingomyelin (d17:1/16:0, d18:1/15:0, d16:1/17:0) | 0.57 | 0.53 | 1.25 |
| sphingomyelin (d18:1/21:0, d17:1/22:0, d16:1/23:0) | 0.62 | 0.67 | 1.24 |
| sphingomyelin (d18:2/18:1) | 0.76 | 0.5 | 1.24 |
| 1-(1-enyl-palmitoyl)-gpc (p-16:0) | 0.74 | 0.62 | 1.23 |
| n-acetyl-beta-alanine | 0.77 | 0.78 | 1.21 |
| sphingomyelin (d18:2/23:1) | 0.69 | 0.58 | 1.21 |
| sphingomyelin (d18:1/17:0, d17:1/18:0, d19:1/16:0) | 0.6 | 0.57 | 1.2 |
| sphingomyelin (d18:1/14:0, d16:1/16:0) | 0.65 | 0.67 | 1.19 |
| 1-(1-enyl-palmitoyl)-2-arachidonoyl-gpc (p-16:0/20:4) | 0.59 | 0.39 | 1.18 |
| sphingomyelin (d18:1/19:0, d19:1/18:0) | 0.58 | 0.6 | 1.18 |
| palmitoyl-sphingosine-phosphoethanolamine (d18:1/16:0) | 1.31 | 0.73 | 1.12 |
| sphingomyelin (d18:2/24:1, d18:1/24:2) | 1.32 | 0.75 | 1.08 |

| **Supplementary Table 15**. Metabolite subclasses associated with vegan relative to non-vegetarian diet in n=139 AHS-2 participants^1^ | | | | | |  |
| --- | --- | --- | --- | --- | --- | --- |
| **Subclass Labels** | **Fold Change (95% CI)** | **n total metabolites** | **n significant metabolites** | **n significant >1** | **n significant < 1** | **FDR** |
| Vitamin A Metabolism | 1.76 (1.37, 2.27) | 5 | 3 | 3 | 0 | <0.0001 |
| Acetylated Peptides | 1.63 (1.26, 2.11) | 3 | 5 | 4 | 1 | 0.004208 |
| Ceramides | 0.77 (0.66, 0.90) | 9 | 11 | 1 | 10 | 0.003341 |
| Dihydroceramides | 0.73 (0.63, 0.85) | 2 | 5 | 0 | 5 | 0.000275 |
| Xanthine Metabolism | 0.73 (0.58, 0.93) | 13 | 6 | 0 | 6 | 0.019089 |
| Fatty Acid, Branched | 0.71 (0.59, 0.86) | 3 | 2 | 0 | 2 | 0.001845 |
| Lysoplasmalogen | 0.70 (0.52, 0.94) | 4 | 1 | 0 | 1 | 0.031187 |
| Lysine Metabolism | 0.68 (0.57, 0.82) | 16 | 6 | 0 | 6 | 0.000283 |
| Plasmalogen | 0.68 (0.56, 0.82) | 11 | 3 | 0 | 3 | 0.000248 |
| Histidine Metabolism | 0.67 (0.54, 0.83) | 15 | 4 | 0 | 4 | 0.001139 |
| Fatty Acid Metabolism (Acyl Carnitine, Long Chain Saturated) | 0.67 (0.52, 0.86) | 8 | 2 | 0 | 2 | 0.004036 |
| Phenylalanine Metabolism | 0.56 (0.36, 0.84) | 6 | 10 | 0 | 10 | 0.012244 |
| Leucine, Isoleucine and Valine Metabolism | 0.53 (0.42, 0.68) | 28 | 7 | 0 | 7 | 0.000124 |
| Fatty Acid Metabolism (also BCAA Metabolism) | 0.52 (0.39, 0.70) | 4 | 1 | 0 | 1 | 0.000165 |
| Fatty Acid Metabolism (Acyl Glycine) | 0.49 (0.41, 0.58) | 7 | 7 | 1 | 6 | 0.000495 |
| Drug - Analgesics, Anesthetics | 0.33 (0.21, 0.52) | 6 | 3 | 0 | 3 | 9.90E-05 |
| Monoacylglycerol | 0.23 (0.16, 0.33) | 16 | 3 | 0 | 3 | 0.000248 |
| Chemical | 0.22 (0.08, 0.62) | 20 | 4 | 0 | 4 | 0.00983 |
| Fatty Acid Metabolism (Acyl Carnitine, Medium Chain) | 0.12 (0.05, 0.25) | 5 | 12 | 0 | 12 | 0.000165 |

^1^Adjustment for race, sex, age, BMI, batch, study, and batch*study

| **Supplementary Table 16**. Metabolite subclasses associated with pesco-vegetarian relative to non-vegetarian diet in n=139 AHS-2 participants^1^ | | | | | | |
| --- | --- | --- | --- | --- | --- | --- |
| **Subclass Labels** | **Fold Change (95% CI)** | **n total metabolites** | **n significant metabolites** | **n significant >1** | **n significant < 1** | **FDR** |
| Lysoplasmalogen | 0.60 (0.50, 0.74) | 4 | 4 | 0 | 4 | 1.8E-04 |
| Xanthine Metabolism | 0.17 (0.08, 0.39) | 13 | 9 | 0 | 9 | 2.7E-04 |
| Plasmalogen | 0.53 (0.43, 0.64) | 11 | 11 | 0 | 11 | 2.7E-04 |
| Histidine Metabolism | 0.54 (0.46, 0.65) | 15 | 6 | 0 | 6 | 5.4E-04 |
| Sphingomyelins | 0.65 (0.53, 0.80) | 29 | 24 | 0 | 24 | 5.4E-04 |
| Phospholipid Metabolism | 0.67 (0.54, 0.83) | 6 | 2 | 0 | 2 | 1.6E-03 |
| Fatty Acid, Branched | 0.51 (0.36, 0.74) | 3 | 2 | 0 | 2 | 1.7E-03 |
| Purine Metabolism, Guanine containing | 0.74 (0.62, 0.89) | 3 | 1 | 0 | 1 | 7.1E-03 |
| Lysine Metabolism | 0.78 (0.67, 0.92) | 16 | 4 | 0 | 4 | 1.3E-02 |
| Dihydrosphingomyelins | 0.69 (0.53, 0.91) | 5 | 2 | 0 | 2 | 2.5E-02 |
| Acetylated Peptides | 0.56 (0.34, 0.90) | 3 | 2 | 0 | 2 | 4.3E-02 |
| Pentose Metabolism | 0.67 (0.49, 0.93) | 6 | 2 | 0 | 2 | 4.4E-02 |
| Aminosugar Metabolism | 0.80 (0.66, 0.96) | 5 | 2 | 0 | 2 | 4.5E-02 |
| Dihydroceramides | 0.69 (0.51, 0.95) | 2 | 1 | 0 | 1 | 4.6E-02 |
| Pyrimidine Metabolism, Orotate containing | 0.58 (0.36, 0.92) | 3 | 1 | 0 | 1 | 4.8E-02 |

^1^Adjustment for race, sex, age, BMI, batch, study, and batch*study

| **Supplementary Table 17**. Metabolite subclasses associated with vegan relative to pesco-vegetarian diet in n=139 AHS-2 participants^1^ | | | | | |  |
| --- | --- | --- | --- | --- | --- | --- |
| **Subclass Labels** | **Fold Change (95% CI)** | **n total metabolites** | **n significant metabolites** | **n significant >1** | **n significant < 1** | **FDR** |
| Diacylglycerol | 2.01 (1.36, 2.99) | 11 | 8 | 8 | 0 | 8.4E-03 |
| Vitamin A Metabolism | 1.88 (1.42, 2.50) | 5 | 5 | 5 | 0 | 4.8E-04 |
| Phosphatidylethanolamine (PE) | 1.59 (1.17, 2.15) | 12 | 8 | 8 | 0 | 1.3E-02 |
| Ascorbate and Aldarate Metabolism | 1.55 (1.20, 2.00) | 6 | 2 | 2 | 0 | 8.2E-03 |
| Phosphatidylcholine (PC) | 1.42 (1.16, 1.74) | 19 | 14 | 13 | 1 | 7.0E-03 |
| Lactosylceramides (LCER) | 1.40 (1.14, 1.72) | 3 | 2 | 2 | 0 | 6.9E-03 |
| Pyrimidine Metabolism, Cytidine containing | 1.36 (1.12, 1.66) | 5 | 1 | 1 | 0 | 8.8E-03 |
| Phospholipid Metabolism | 1.33 (1.06, 1.68) | 6 | 2 | 2 | 0 | 4.1E-02 |
| Plasmalogen | 1.30 (1.06, 1.59) | 11 | 5 | 5 | 0 | 3.9E-02 |
| Aminosugar Metabolism | 1.29 (1.06, 1.57) | 5 | 1 | 1 | 0 | 3.4E-02 |
| Glycine, Serine and Threonine Metabolism | 1.21 (1.07, 1.38) | 10 | 3 | 2 | 1 | 1.3E-02 |
| Tryptophan Metabolism | 0.79 (0.64, 0.97) | 20 | 8 | 1 | 7 | 4.1E-02 |
| Gamma-glutamyl Amino Acid | 0.78 (0.66, 0.93) | 15 | 10 | 1 | 9 | 1.7E-02 |
| Creatine Metabolism | 0.77 (0.66, 0.91) | 3 | 1 | 0 | 1 | 1.1E-02 |
| Glycolysis, Gluconeogenesis, and Pyruvate Metabolism | 0.77 (0.62, 0.97) | 5 | 2 | 0 | 2 | 4.3E-02 |
| Leucine, Isoleucine and Valine Metabolism | 0.75 (0.63, 0.90) | 28 | 13 | 0 | 13 | 1.1E-02 |
| Phenylalanine Metabolism | 0.74 (0.60, 0.92) | 6 | 2 | 0 | 2 | 1.7E-02 |
| Tyrosine Metabolism | 0.74 (0.60, 0.92) | 21 | 9 | 1 | 8 | 2.0E-02 |
| Chemical | 0.73 (0.56, 0.96) | 20 | 4 | 1 | 3 | 4.0E-02 |
| Glutathione Metabolism | 0.72 (0.57, 0.91) | 6 | 2 | 0 | 2 | 1.8E-02 |
| Fatty Acid Metabolism (Acyl Carnitine, Monounsaturated) | 0.71 (0.54, 0.93) | 9 | 3 | 0 | 3 | 2.9E-02 |
| Long Chain Saturated Fatty Acid | 0.69 (0.51, 0.94) | 7 | 4 | 0 | 4 | 4.2E-02 |
| Ceramides | 0.66 (0.50, 0.87) | 9 | 5 | 0 | 5 | 1.6E-02 |
| Fatty Acid, Dicarboxylate | 0.64 (0.51, 0.79) | 33 | 11 | 2 | 9 | 1.2E-03 |
| Fatty Acid Metabolism (Acyl Carnitine, Long Chain Saturated) | 0.60 (0.47, 0.76) | 8 | 6 | 0 | 6 | 1.4E-03 |
| Fatty Acid Metabolism (Acyl Carnitine, Polyunsaturated) | 0.60 (0.40, 0.88) | 8 | 3 | 0 | 3 | 2.3E-02 |
| Monoacylglycerol | 0.48 (0.30, 0.76) | 16 | 11 | 1 | 10 | 1.2E-02 |
| Fatty Acid, Branched | 0.45 (0.31, 0.67) | 3 | 2 | 0 | 2 | 9.6E-04 |
| ^1^Adjustment for race, sex, age, BMI, batch, study, and batch*study | | | | | | |

| **Supplementary Table 18**. Metabolite subclasses associated with both creatinine and vegan (relative to pesco-vegetarian) dietary pattern at FDR < 0.05^1^ | | | | |
| --- | --- | --- | --- | --- |
| **Subclass Labels** | **Vegan, Fold Change (95% CI)** | **FDR** | **Creatinine, Fold Change (95% CI)** | **FDR** |
| Fatty Acid Metabolism (Acyl Carnitine, Long Chain Saturated) | 0.60 (0.47, 0.76) | 1.4E-03 | 1.20 (1.04, 1.38) | 1.1E-02 |
| Fatty Acid, Dicarboxylate | 0.64 (0.51, 0.79) | 1.2E-03 | 1.14 (1.01, 1.29) | 1.9E-02 |
| Ceramides | 0.66 (0.50, 0.87) | 1.6E-02 | 1.25 (1.06, 1.47) | 6.4E-03 |
| Fatty Acid Metabolism (Acyl Carnitine, Monounsaturated) | 0.71 (0.54, 0.93) | 2.9E-02 | 1.17 (1.02, 1.34) | 1.5E-02 |
| Chemical | 0.73 (0.56, 0.96) | 4.0E-02 | 1.33 (1.16, 1.51) | 4.0E-04 |
| Phenylalanine Metabolism | 0.74 (0.60, 0.92) | 1.7E-02 | 1.24 (1.11, 1.39) | 8.0E-04 |
| Tyrosine Metabolism | 0.74 (0.60, 0.92) | 2.0E-02 | 1.21 (1.11, 1.33) | 4.4E-04 |
| Leucine, Isoleucine and Valine Metabolism | 0.75 (0.63, 0.90) | 1.1E-02 | 1.21 (1.11, 1.31) | 3.7E-04 |
| Creatine Metabolism | 0.77 (0.66, 0.91) | 1.1E-02 | 1.06 (0.98, 1.14) | 4.8E-02 |
| Tryptophan Metabolism | 0.79 (0.64, 0.97) | 4.1E-02 | 1.22 (1.11, 1.34) | 4.7E-04 |
| Aminosugar Metabolism | 1.29 (1.06, 1.57) | 3.4E-02 | 1.32 (1.20, 1.44) | <3.7E-04 |
| Plasmalogen | 1.30 (1.06, 1.59) | 3.9E-02 | 1.21 (1.09, 1.34) | 1.1E-03 |
| Phospholipid Metabolism | 1.33 (1.06, 1.68) | 4.1E-02 | 1.26 (1.15, 1.37) | 9.8E-05 |
| Pyrimidine Metabolism, Cytidine containing | 1.36 (1.12, 1.66) | 8.8E-03 | 1.16 (1.03, 1.31) | 1.1E-02 |
| Ascorbate and Aldarate Metabolism | 1.55 (1.20, 2.00) | 8.2E-03 | 1.24 (1.10, 1.40) | 1.6E-03 |
| Phosphatidylethanolamine (PE) | 1.59 (1.17, 2.15) | 1.3E-02 | 1.20 (1.01, 1.41) | 1.8E-02 |
| Vitamin A Metabolism | 1.88 (1.42, 2.50) | 4.8E-04 | 1.14 (0.96, 1.35) | 4.7E-02 |
| ^1^Includes adjustment for race, BMI, sex, age, batch, study, and batch*study | | | | |

| **Supplementary Table 19.** Metabolite subclasses associated with creatinine (90th vs 10th percentile), vegan and pesco-vegetarian (relative to non-vegetarian) dietary pattern at FDR < 0.05^1,2^ | | | | | | |
| --- | --- | --- | --- | --- | --- | --- |
| **Subclass** | **Vegan, Fold Difference (95% CI)** | **FDR** | **Pesco-vegetarian, Fold Difference (95% CI)** | **FDR** | **Creatinine, Fold Difference (95% CI)** | **FDR** |
| Acetylated Peptides | 0.33 (0.21, 0.52) | 9.9E-05 | 0.56 (0.34, 0.90) | 4.3E-02 | 1.42 (1.02, 1.98) | 1.9E-02 |
| Histidine Metabolism | 0.49 (0.41, 0.58) | 5.0E-04 | 0.54 (0.46, 0.65) | 5.4E-04 | 1.55 (1.37, 1.75) | <0.0001 |
| Plasmalogen | 0.68 (0.57, 0.82) | 2.8E-04 | 0.53 (0.43, 0.64) | 2.7E-04 | 1.21 (1.09, 1.34) | 1.1E-03 |
| Lysoplasmalogen | 0.68 (0.56, 0.82) | 2.5E-04 | 0.60 (0.50, 0.74) | 1.8E-04 | 1.18 (1.05, 1.33) | 5.8E-03 |
| Lysine Metabolism | 0.73 (0.63, 0.85) | 2.8E-04 | 0.78 (0.67, 0.92) | 1.3E-02 | 1.35 (1.22, 1.50) | <0.0001 |
| ^1^Individual component metabolite values averaged across their respective parent subclass to obtain a composite value; fold difference calculated from ratio of adjusted geometric means corresponding to dietary pattern or medians of extreme quintiles for creatinine | | | | | | |
| ^2^Adjustment for race, BMI, sex, age, batch, study, batch*study interaction | | | | | | |
