# Supplementary material for: Vegetarian Dietary Patterns and Diet-Related Metabolites Are Associated With Kidney Function in the Adventist Health Study-2 Cohort
Source: J Ren Nutr. Author manuscript; Available in PMC 2026 Jul 17. (PMC13379062; doi:10.1053/j.jrn.2025.11.001)

**Figure Legends**

Supplementary Figure 1. Venn diagrams of numbers of significant metabolites in analyses of dietary pattern associations or creatinine (90^th^ vs 10^th^ percentile) with metabolites, and number of overlapping metabolites. Metabolite level comparisons are shown in A, and metabolite subclass level comparisons shown in B. Pesco=pesco-vegetarian; NV=nonvegetarian.

Supplementary Figure 2. Fold change of metabolites showing differential abundance in analyses comparing vegan and pesco-vegetarian with non-vegetarian dietary patterns and 90^th^ vs 10^th^ percentiles of creatinine. The vast majority of compounds associated with diet group and creatinine in opposite directions, i.e. inversely with vegan and pesco-vegetarian relative to non-vegetarian dietary pattern and positively with creatinine concentration.


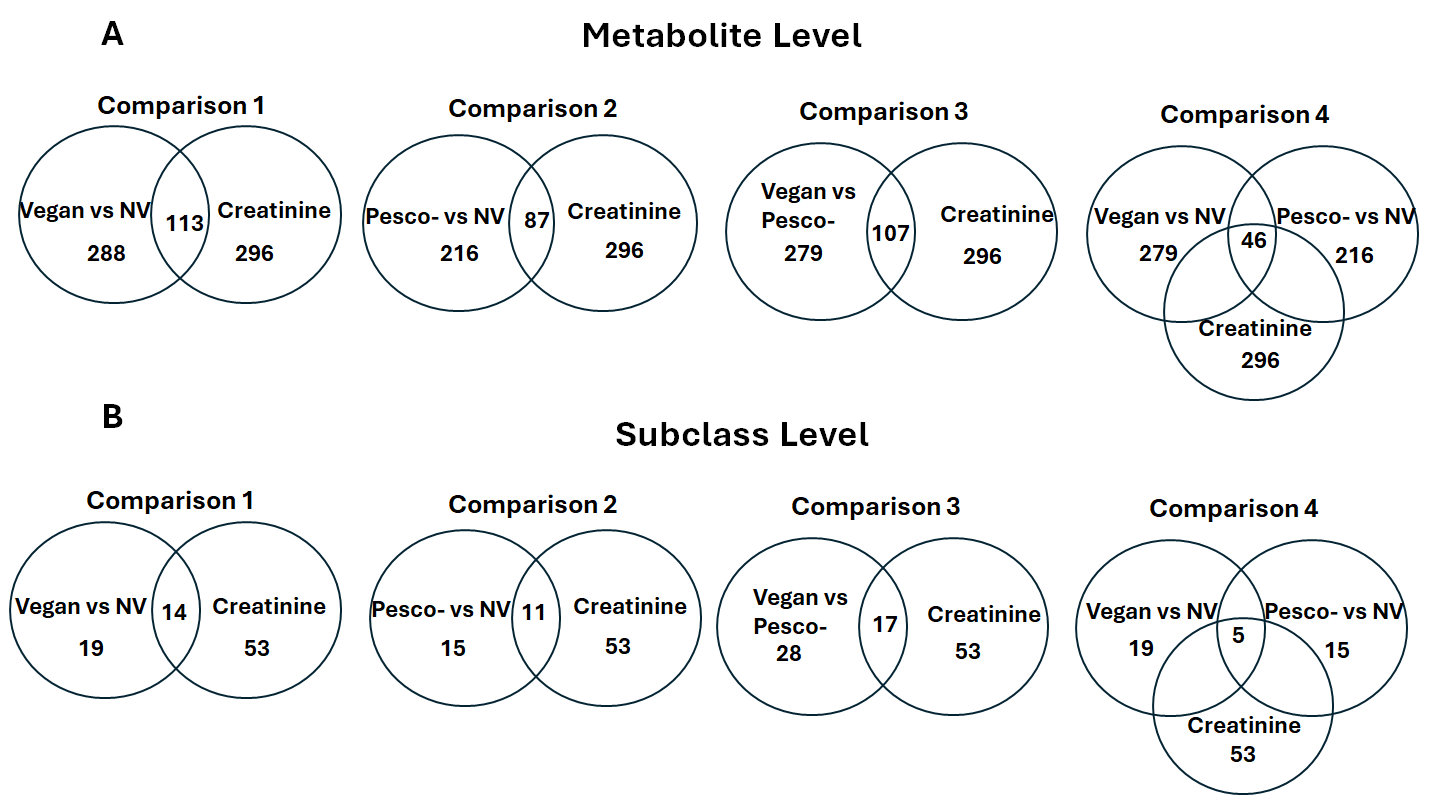


Supplementary Figure 1

Supplementary Figure 2


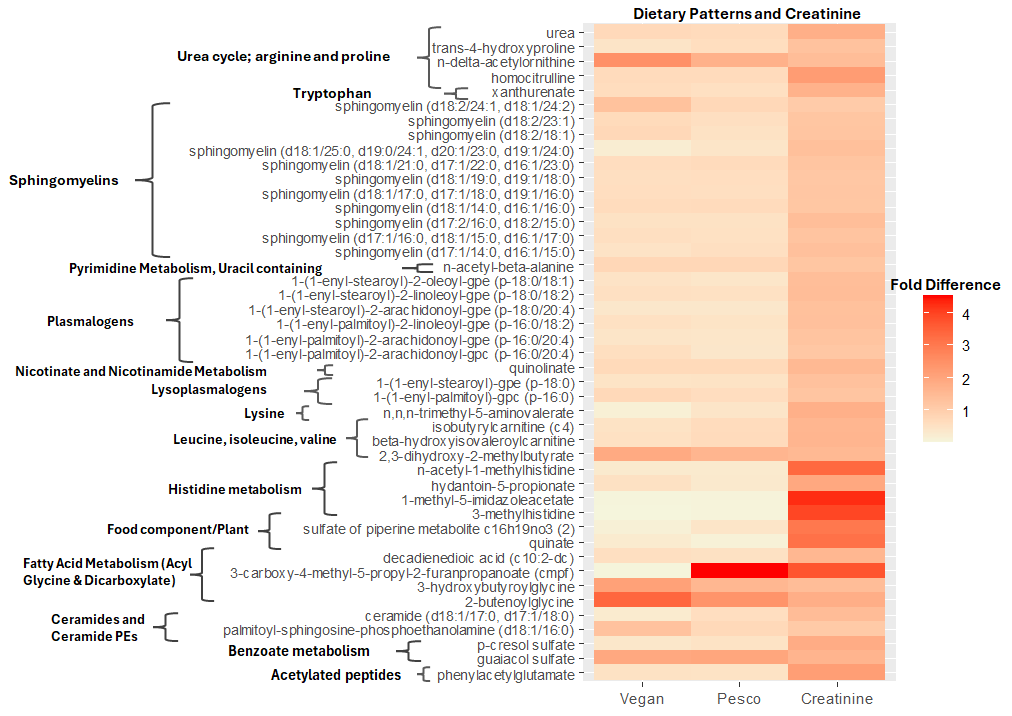

Supplement: 2 [file NIHMS2188991-supplement-2.docx]
